# Colossal barocaloric effects with ultralow hysteresis in two-dimensional metal–halide perovskites

**DOI:** 10.1038/s41467-022-29800-9

**Published:** 2022-05-09

**Authors:** Jinyoung Seo, Ryan D. McGillicuddy, Adam H. Slavney, Selena Zhang, Rahil Ukani, Andrey A. Yakovenko, Shao-Liang Zheng, Jarad A. Mason

**Affiliations:** 1 Department of Chemistry and Chemical Biology, Harvard University, Cambridge, MA 02138 USA; 2 X-ray Science Division, Advanced Photon Source, Argonne National Laboratory, Argonne, IL 60439 USA

**Keywords:** Materials chemistry, Energy, Materials for energy and catalysis

## Abstract

Pressure-induced thermal changes in solids—barocaloric effects—can be used to drive cooling cycles that offer a promising alternative to traditional vapor-compression technologies. Efficient barocaloric cooling requires materials that undergo reversible phase transitions with large entropy changes, high sensitivity to hydrostatic pressure, and minimal hysteresis, the combination of which has been challenging to achieve in existing barocaloric materials. Here, we report a new mechanism for achieving colossal barocaloric effects that leverages the large volume and conformational entropy changes of hydrocarbon order–disorder transitions within the organic bilayers of select two-dimensional metal–halide perovskites. Significantly, we show how the confined nature of these order–disorder phase transitions and the synthetic tunability of layered perovskites can be leveraged to reduce phase transition hysteresis through careful control over the inorganic–organic interface. The combination of ultralow hysteresis and high pressure sensitivity leads to colossal reversible isothermal entropy changes (>200 J kg^−1^ K^−1^) at record-low pressures (<300 bar).

## Abstract (web summary)

Barocaloric materials, undergoing thermal changes in response to applied pressure, may provide energy efficient and zero-emission solid-state cooling. Here the authors report a mechanism for achieving large reversible barocaloric effects near ambient temperature, leveraging volume and conformational entropy changes within the organic bilayers of two-dimensional metal–halide perovskites.

## Introduction

Nearly 4400 TWh of electricity—20% of the total consumed in the world—is used each year by refrigerators, air conditioners, and heat pumps for cooling^1^. In addition to the 2.3 Gt of carbon dioxide emitted during the generation of this electricity, the vapor-compression-based devices that provided the bulk of this cooling emitted fluorocarbon refrigerants with a global warming potential equivalent to 1.5 Gt of carbon dioxide into the atmosphere^2^. With population and economic growth expected to dramatically increase over the next several decades, the development of alternative cooling technologies with improved efficiency and reduced emissions will be critical to meeting global cooling needs in a more sustainable fashion^3^.

Caloric materials, which undergo thermal changes in response to an applied magnetic, electric, or stress field, offer the potential for solid-state cooling with high energy efficiency and zero direct emissions, as well as faster start-up times, quieter operation, greater amenability to miniaturization, and better recyclability than conventional vapor-compression systems^4–6^. Caloric effects are particularly strong in ferroic materials near first-order phase transitions, where small changes to the magnitude of an applied field can induce the large isothermal entropy and adiabatic temperature changes required for commercially viable cooling cycles.

Although magnetocaloric and electrocaloric effects have received the most attention for solid-state cooling, transitions between states with different magnetic ordering or electric polarization typically involve much smaller entropy changes than those that occur during the liquid–vapor transition of a hydrofluorocarbon refrigerant and can be complicated by the need to generate large magnetic fields or to avoid dielectric breakdown^7,8^. Alternatively, the sensitivity of structural phase transitions to hydrostatic pressure and uniaxial strain can be leveraged to realize even larger entropy changes in barocaloric and elastocaloric materials, respectively^9,10^. In particular, reversible structural transitions that are accompanied by a substantial change in volume can produce barocaloric effects with large entropy changes and high sensitivity to pressure.

An ideal material for barocaloric cooling would feature a phase transition with (i) a large isothermal entropy change (Δ*S*_it_) and adiabatic temperature change (Δ*T*_ad_), (ii) a large barocaloric coefficient (d*T*_tr_/d*P*) that reflects a high sensitivity of the transition temperature, *T*_tr_, to pressure, *P*, and (iii) a low reversible pressure, *P*_rev_, which represents the minimum pressure required to induce a reversible entropy change and is proportional, in part, to the thermal hysteresis of the phase transition^11^. Among the limited range of compounds that have been investigated as candidate barocaloric materials, certain organic plastic crystals, such as neopentylglycol, have recently been shown to exhibit order–disorder phase transitions in the solid state that yield colossal barocaloric effects with entropy changes approaching those of commercial hydrofluorocarbon refrigerants^11–13^. These transitions, however, often have large thermal hysteresis, occur away from ambient temperature, or have only moderate sensitivity to applied pressure. This highlights a longstanding challenge across all classes of caloric materials: field-induced phase transitions that lead to large isothermal entropy and adiabatic temperature changes are often accompanied by substantial hysteresis, which increases the magnitude of the applied field required to capture the full entropy of the transition and reduces the efficiency of each cooling cycle^14–17^.

In pursuit of a new strategy to target barocaloric effects with low hysteresis, high sensitivity to pressure, and large entropy changes near ambient temperature, we sought a phase transition mechanism that would offer access to large volume changes and disordered states with high entropy but could take place within a microenvironment tailored to promote reversibility. To this end, we recognized that long-chain hydrocarbons undergo order–disorder transitions with far higher entropy changes than have ever been realized in a caloric effect. For instance, the entropy of *n*-decane (C_10_H_22_) increases by 829 J kg^–1^ K^–1^ (118 J mol^–1^ K^–1^) upon melting^18^, which is even larger than the 520 J kg^–1^ K^–1^ (53 J mol^–1^ K^–1^) increase in entropy of the commercial refrigerant HFC-134a (CH_2_FCF_3_) upon vaporization^19^. These high entropy changes arise from increases in orientational, conformational, and positional entropy as molecules restricted in the crystalline state gain access to more rotational, vibrational, and translational degrees of freedom in the disordered—and expanded—molten state^20^.

**Fig. 1:**
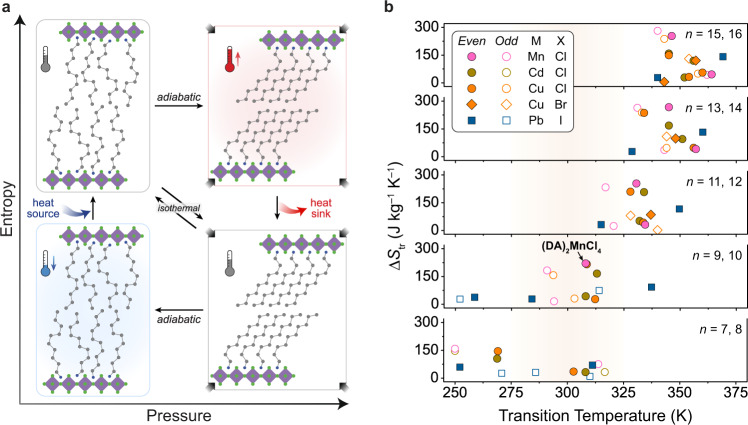
Barocaloric cooling with two-dimensional (2-D) metal–halide perovskites. **a** Illustration of how the pressure dependence of hydrocarbon order–disorder transitions in the organic bilayers of hybrid 2-D perovskites can be leveraged to drive a barocaloric cooling cycle. Each cooling cycle begins with an adiabatic (Brayton-like cycle) or isothermal (Stirling-like cycle) increase in pressure that induces a first-order phase transition from an expanded, high-entropy phase of the 2-D perovskite to a contracted, low-entropy phase. Heat released during this exothermic transition is dissipated to a heat sink, returning the material to its original temperature but now at a lower entropy. The pressure is then adiabatically or isothermally decreased to reverse the phase transition and cool a heat source. **b** Comparison of phase transition entropies, Δ*S*_tr_, and transition temperatures, *T*_tr_, for order–disorder transitions in select 2-D perovskites of the form (C_*n*_H_2*n*+1_NH_3_)_2_MX_4_ (*n* = 7–16; M = Mn, Cd, Cu, or Pb; X = Cl, Br, or I). Thermally induced phase transitions in 2-D perovskites are often accompanied by large changes in entropy that are sensitive to the length of the hydrocarbon chain and the identity of the metal and halide in the inorganic layer. Many of these order–disorder transitions involve one or more minor transitions that occur at lower or higher temperatures than the major transition, but (DA)_2_MnCl_4_ (DA = decylammonium) features a single order–disorder transition with a large entropy change near ambient temperature.

The entropy and latent heat changes associated with hydrocarbon melting transitions are already exploited for thermal energy storage in paraffin-based solid–liquid phase-change materials^21^. Similar types of phase transitions are also known to occur in the solid state in several classes of layered materials, including two-dimensional (2-D) metal–halide perovskites of the form (R-NH_3_)_2_MX_4_ (R = C_*n*_H_2*n*+1_; M = Mn, Fe, Cu, Cd, Pb; X = Cl, Br, or I)^22,23^. In these compounds, sheets of corner-sharing MX_6_ octahedra create anionic pockets—defined by the axial halides of four adjacent metal centers—that template the arrangement of bilayers of alkylammonium cations through charge-assisted hydrogen bonds. When long-chain hydrocarbon molecules (*n* > 3) are incorporated, many layered perovskites undergo thermally induced, first-order phase transitions between low- and high-entropy states driven by a partial disordering transition of the hydrocarbon bilayers (Supplementary Tables 1–3). These transitions are often referred to as “chain-melting” transitions because of the liquid-like conformational degrees of freedom associated with the hydrocarbon chains in the disordered phase, even though the transition occurs entirely in the solid state^23,24^. As such, we anticipated that 2-D perovskites would serve as a highly tunable solid-state platform to leverage the large changes in entropy and enthalpy that accompany hydrocarbon order–disorder transitions for barocaloric cooling (Fig. 1a). Moreover, since the inorganic layers and organic bilayers of 2-D perovskites can be independently manipulated, we further expected that phase transition hysteresis could be minimized through confinement effects and careful control of the organic–inorganic interfaces. We note that 2-D perovskites were also independently identified as promising barocaloric materials by Lloveras and coworkers while our work was under peer review^25^.

Here, we report a comprehensive study of barocaloric effects in two representative 2-D perovskites (DA)_2_MnCl_4_ (DA = decylammonium) and (NA)_2_CuBr_4_ (NA = nonylammonium). Through a combination of high-pressure calorimetry and X-ray diffraction experiments, we show that these materials feature phase transitions with large entropy changes, high sensitivity to pressure, and minimal hysteresis, which collectively leads to outstanding barocaloric performance. Moreover, we demonstrate how the chemical tunability of 2-D perovskites and the decoupling of extrinsic and intrinsic factors that influence phase-change kinetics can be leveraged to drive reversible barocaloric effects with pressures approaching those used in vapor-compression cooling systems. These results are further supported by detailed crystallographic and spectroscopic characterization, which provide molecular-level insights into barocaloric effects in 2-D perovskites, including compensation effects between entropy changes, hysteresis, and pressure sensitivity. Our work establishes hydrocarbon phase transitions in 2-D perovskites as a generalizable mechanism for achieving large and reversible barocaloric effects at low driving pressures.

## Results

### Pressure dependence of phase transitions in (DA)_2_MnCl_4_

**Fig. 2:**
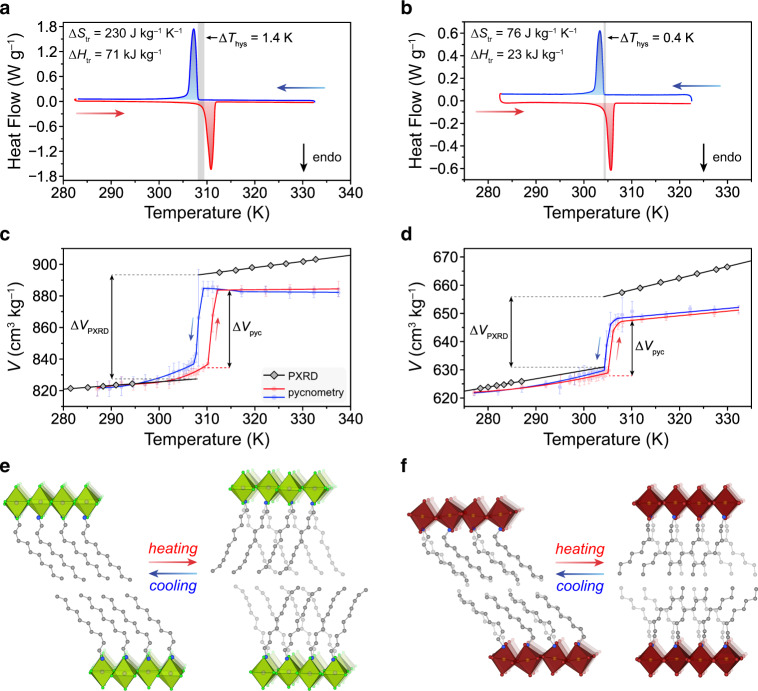
Thermally induced hydrocarbon order–disorder transitions in (DA)_2_MnCl_4_ and (NA)_2_CuBr_4_ at ambient pressure. Differential scanning calorimetry (DSC) traces for powder samples of (**a**) (DA)_2_MnCl_4_ and (**b**) (NA)_2_CuBr_4_ at 1 bar with heating (red) and cooling (blue) rates of 2 K min^−1^. Thermal hysteresis (Δ*T*_hys_) is indicated by the vertical gray bars. Note that Δ*T*_hys_ is calculated as the difference between heating and cooling transition onset temperatures, with Δ*T*_hys_ = *T*_tr,heating_ – *T*_tr,cooling_. Specific volumes obtained from variable-temperature powder X-ray diffraction and He pycnometry measurements are used to determine the volume changes, Δ*V*, that accompany the order–disorder transition for (**c**) (DA)_2_MnCl_4_ and (**d**) (NA)_2_CuBr_4_. Variable-temperature crystal structures of the low-temperature (LT) and high-temperature (HT) phases of (**e**) (DA)_2_MnCl_4_ and (**f**) (NA)_2_CuBr_4_. Note that the LT crystal structures were both obtained at 270 K, while the HT crystal structures were obtained at 330 K and 335 K for (DA)_2_MnCl_4_ and (NA)_2_CuBr_4_, respectively. Purple, orange, green, brown, gray, and blue spheres represent Mn, Cu, Cl, Br, C, and N atoms, respectively. H atoms are omitted for clarity. Note that DA chains are disordered over a special position in both the LT and HT phases, while NA chains are modeled with two-part disorder in the LT phase and disordered over a special position in the HT phase. Geometric parameters obtained from single-crystal X-ray diffraction experiments are summarized in Supplementary Tables 14–22.

The 2-D perovskite (DA)_2_MnCl_4_ was selected as a potential barocaloric material because of its large phase transition entropy (Δ*S*_tr_ = 230 J kg^−1^ K^−1^) and enthalpy (Δ*H*_tr_ = 71 kJ kg^−1^), near-ambient phase transition temperature (*T*_tr_ = 310 K), and lightweight, nontoxic elemental composition (Fig. 1b). At ambient temperature and pressure, (DA)_2_MnCl_4_ adopts an ordered monoclinic structure (low-temperature, LT, phase) with bilayers of hydrocarbon chains—each of which contains a single gauche C–C bond (C2–C3) and seven trans C–C bonds—aligned parallel to one another and tilted 48.3(1)° with respect to the Mn–Cl_eq_ (equatorial chloride) plane (Fig. 2e)^26^. Upon heating above 310 K, the compound undergoes a first-order phase transition to an expanded orthorhombic lattice (high-temperature, HT, phase) with disordered hydrocarbon chains that have increased conformational and rotational degrees of freedom^27^. The large increase in entropy during the transition can be attributed to flipping of the alkylammonium cations between two favorable orientations within the Mn–Cl pockets, internal rotations of C–C bonds that create dynamically disordered conformational defects within the hydrocarbon chains, and a substantial increase in volume (Supplementary Notes 1–3)^24^.

Differential scanning calorimetry (DSC) measurements at ambient pressure showed that the hydrocarbon order–disorder transition is sharp and fully reversible with a thermal hysteresis, Δ*T*_hys_, of just 1.4 K for a microcrystalline powder sample at a scan rate of 2 K min^−1^ (Fig. 2a). Variable-temperature powder X-ray diffraction (PXRD) experiments at ambient pressure revealed that the phase transition is accompanied by an increase in interlayer distance of 1.88 Å (7.0%) as the alkylammonium cations tilt further away from the Mn–Cl_eq_ plane to create additional space between disordered hydrocarbon chains in the HT phase (Supplementary Fig. 1). This expansion leads to an 8.0% overall increase in the volume of the compound (Δ*V*_tr_ = 65.9 cm^3^ kg^−1^; Fig. 2c). Based on the measured volume and entropy changes, the Clausius–Clapeyron relation, d*T*/d*P* = Δ*V*_tr_/Δ*S*_tr_, can be used to predict a barocaloric coefficient for (DA)_2_MnCl_4_ of 28.7 K kbar^−1^, which would represent one of the highest values reported for a barocaloric material (Supplementary Table 5). We note that select 3-D hybrid perovskites also exhibit similarly high barocaloric coefficients^28,29^.

**Fig. 3:**
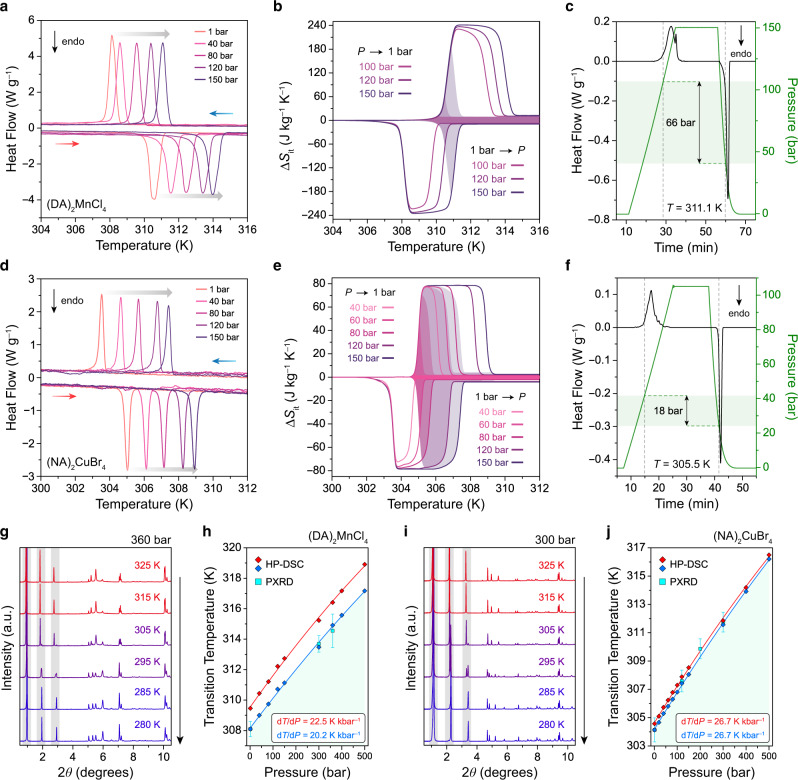
Barocaloric effects in 2-D metal–halide perovskites. DSC measurements under applied hydrostatic pressure for single-crystal samples of (**a**) (DA)_2_MnCl_4_ and (**d**) (NA)_2_CuBr_4_ with heating and cooling rates of 2 K min^−1^. Isothermal entropy changes, Δ*S*_it_, are calculated by the quasi-direct method for (**b**) (DA)_2_MnCl_4_ and (**e**) (NA)_2_CuBr_4_ for compression from ambient pressure and for decompression to ambient pressure. The shaded area indicates the reversible Δ*S*_it_ within this pressure range. Isobaric entropy curves are shown in Supplementary Figs. 10 and 11. Direct evaluation of pressure hysteresis, Δ*P*_hys_, through quasi-isothermal DSC experiments for (**c**) (DA)_2_MnCl_4_ and (**f**) (NA)_2_CuBr_4_ at 311 K and 306 K, with pressure cycling from 1 to 150 bar and to 105 bar, respectively. Δ*P*_hys_ is calculated as the difference between the onset pressure for the compression-induced exotherm and the decompression-induced endotherm and is indicated by the horizontal green bar. Variable-temperature powder X-ray diffraction (PXRD) patterns for (**g**) (DA)_2_MnCl_4_ and (**i**) (NA)_2_CuBr_4_ at 360 bar and 300 bar of He, respectively, while cooling from 325 K to 280 K, with an X-ray wavelength of 0.45237 Å. The pressure dependence of the order–disorder transition temperature as determined by HP-DSC (diamonds) and PXRD (squares) is used to calculate the barocaloric coefficient, d*T*/d*P*, for (**h**) (DA)_2_MnCl_4_ and (**j**) (NA)_2_CuBr_4_. Red and blue symbols indicate the phase transition temperatures during heating and cooling, respectively. Barocaloric coefficients are summarized in Supplementary Table 8.

To directly evaluate the pressure dependence of the phase transition temperature, isobaric DSC experiments were performed under applied hydrostatic pressures of up to 150 bar using He as the pressure-transmitting medium (Fig. 3a). As expected, the phase transition shifts to higher temperatures as the pressure is increased, with a measured d*T*/d*P* of 22.5 ± 0.3 K kbar^–1^ during heating and 20.2 ± 1.1 K kbar^–﻿1^ during cooling (Fig. 3h, Supplementary Fig. 6). Importantly, the application of pressure does not lead to any significant changes to the phase transition width, and Δ*S*_tr_ remains within 96% of its ambient pressure value at 150 bar (Supplementary Fig. 10). Variable temperature and pressure PXRD experiments (performed at beamline 17-BM of the Advanced Photon Source at Argonne National Laboratory) confirm that similar order–disorder transitions still occur at pressures up to at least 360 bar (Fig. 3g, Supplementary Figs. 23–25), with small decreases in overall volume changes at higher pressures that are consistent with a higher compressibility for the HT phase relative to the LT phase (Supplementary Table 9).

Interestingly, the d*T*/d*P* values for (DA)_2_MnCl_4_ determined by PXRD and HP-DSC are lower than those predicted using the Clausius–Clapeyron equation. One possible explanation for this is that the effective volume change in the presence of a pressure-transmitting medium is less than the crystallographic volume change^30^. This could occur if He was excluded from the dense, crystalline organic bilayer of the LT phase but could permeate into the disordered organic bilayer of the HT phase—owing to its increased free volume—which would reduce the amount of additional volume that was occupied by the expanded phase. Indeed, the volume change that is directly measured by He pycnometry is 17 cm^3^ kg^–1^ smaller than that determined by crystallography (Fig. 2c), and using this lower effective volume change in the Clausius–Clapeyron equation yields a predicted d*T*/d*P* of 21.4 ± 1.5 K kbar^–1^ that matches the HP-DSC and PXRD values (Supplementary Table 10). Although effects of the pressure-transmitting medium are not typically considered when evaluating barocaloric materials, this result provides a pathway to realizing a higher d*T*/d*P* by preventing the pressure-transmitting medium from entering the disordered phase through encapsulation or the use of a more sterically bulky fluid. Regardless, the d*T*/d*P* that can be achieved using He to transmit hydrostatic pressure is higher than for many barocaloric materials, which, along with the large Δ*S*_tr_ and small hysteresis, presents considerable advantages for barocaloric cooling.

### Reversible barocaloric effects in (DA)_2_MnCl_4_

**Fig. 4:**
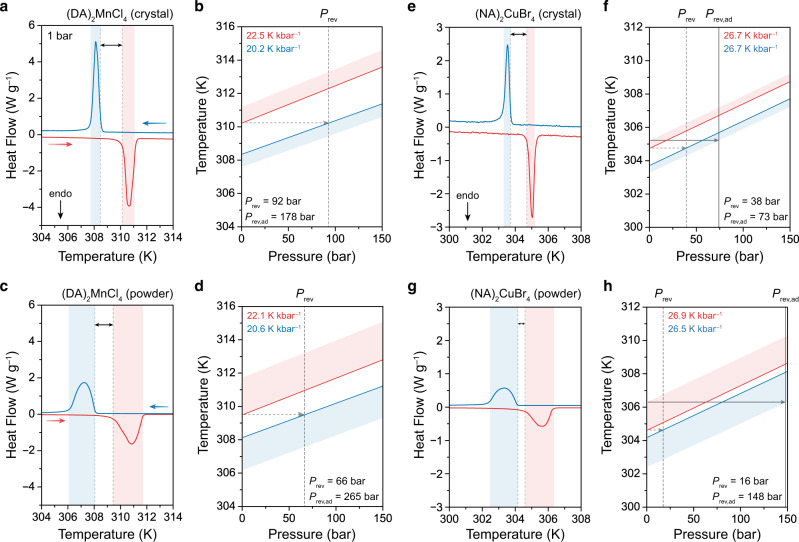
Dependence of low-pressure reversibility on thermal contact. The phase boundary determined from HP-DSC experiments for (**a**–**d**) (DA)_2_MnCl_4_ and (**e**–**h**) (NA)_2_CuBr_4_, using (**a**, **b**, **e**, **f**) single-crystal samples with improved thermal contact and (**c**, **d**, **g**, **h**) powder samples. Scan rates of 2 K min^–1^ were used for all experiments, and He was used as the pressure-transmitting medium. DSC traces at ambient pressure are show in the left panel, with transition peak width highlighted in red and blue shades for heating and cooling, respectively. Onset temperatures are highlighted in dashed gray lines, with thermal hysteresis marked using black arrows. The pressure dependence of the onset and completion transition temperatures is shown in the right panels, and these phase boundaries illustrate the impact of the transition width on the minimum pressure required to drive a reversible isothermal entropy change (*P*_rev_) and a reversible adiabatic temperature change (*P*_rev,ad_). Note that the isobaric HP-DSC data for the powder samples is shown in Supplementary Fig. 16. Reversible barocaloric effects for powder and single-crystal samples are summarized in Supplementary Table 7.

Under the cyclic operating conditions of a barocaloric cooling system, the lowest possible operating pressure will be set by the pressure, *P*_rev_, that must be applied to induce a reversible isothermal entropy change, which can be used to drive a Stirling-like cooling cycle^31^, when cycling to and from ambient pressure. For a normal barocaloric effect, *P*_rev_ corresponds to the pressure at which the onset temperature of the exothermic phase transition at applied pressure is equal to the onset temperature of the endothermic phase transition at 1 bar (ref. ^11^). As such, *P*_rev_ is proportional to the thermal hysteresis at 1 bar and inversely proportional to the barocaloric coefficient for the exothermic transition, with *P*_rev_ = Δ*T*_hys_/(d*T*/d*P*)_cooling_. In addition, the minimum pressure required to induce a reversible adiabatic temperature change, *P*_rev,ad_, corresponds to the pressure at which the completion temperature of the exothermic phase transition at applied pressure is equal to the completion temperature of the endothermic phase transition at 1 bar (Supplementary Note 4). As such, *P*_rev,ad_ is also affected by the transition peak width in addition to the thermal hysteresis and barocaloric coefficient, with smaller peak widths leading to lower *P*_rev,ad_. During our isobaric DSC experiments, we found that the transition peak widths of powder samples of (DA)_2_MnCl_4_ were much broader than those of single-crystal samples (Fig. 4a, c). We attribute the narrower peak widths of the single-crystal samples to improved thermal contact between single crystals and the DSC sample pan, as poor thermal contact is known to lead to thermal gradients that cause increased peak widths^32^. Accordingly, single-crystal samples of (DA)_2_MnCl_4_ with high-quality thermal contact have a lower *P*_rev,ad_ than powder samples (178 bar and 265 bar, respectively), even though powder samples have a slightly lower *P*_rev_ because of their smaller thermal hysteresis (Supplementary Table 7, Supplementary Note 5). This demonstrates the importance of considering the influence of extrinsic factors, such as the quality of thermal contact between the sample and sample holder, when evaluating the intrinsic potential of new barocaloric materials.

Owing to its low Δ*T*_hys_ and high d*T*/d*P*, a single-crystal sample of (DA)_2_MnCl_4_ has a *P*_rev_ of just 92 bar, which ranks among the lowest values reported for barocaloric materials (Supplementary Table 5). This low *P*_rev_ was further confirmed by calculating reversible isothermal entropy changes (Δ*S*_it_) as a function of pressure from the difference between isobaric entropy changes, Δ*S*_ib_, at ambient pressure and elevated pressures. Here, Δ*S*_ib_ values obtained from heating data correspond to the disordering transition induced by a decrease in pressure (Δ*S*_it_ > 0), while Δ*S*_ib_ values from cooling data correspond to the ordering transition induced by an increase in pressure (Δ*S*_it_ < 0) (Supplementary Fig. 10). Excitingly, these Δ*S*_it_ curves confirm that a non-zero reversible entropy change of 13 J kg^−1^ K^−1^ can be induced at 100 bar (Fig. 3b). Moreover, a reversible entropy change of 190 J kg^−1^ K^−1^ can be accessed at a driving pressure of 150 bar (Fig. 3b, Supplementary Fig. 17b, e). To the best of our knowledge, inducing a reversible entropy change of this magnitude through a pressure shift of only 150 bar is unprecedented in barocaloric materials (Supplementary Table 5). As a result, (DA)_2_MnCl_4_ displays a record-high barocaloric strength—defined as the reversible isothermal entropy change normalized by the driving pressure^33^—of 1267 J kg ^−1^ K^−1^ kbar^−1^ at 150 bar (Supplementary Table 6). Based on variable-temperature heat capacity, *c*_p_, measurements at ambient pressure (Supplementary Fig. 31), a maximum adiabatic temperature change, Δ*T*_ad,max_, of 45 K can be estimated from the indirect calculation Δ*T*_ad_(*T*) = –*T*Δ*S*_it_/*c*_p_, which also ranks among the highest values yet reported for barocaloric materials (Supplementary Table 6).

**Fig. 5:**
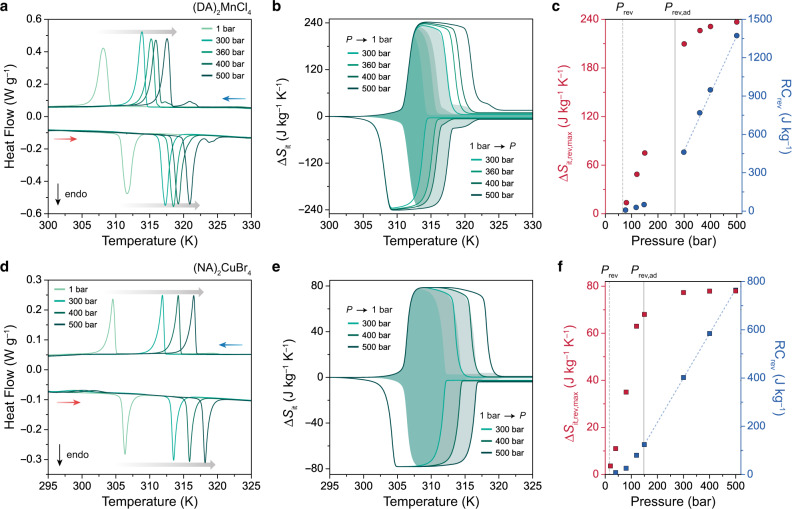
Isobaric DSC experiments from 300–500 bar. DSC measurements under applied hydrostatic pressure for powder samples of (**a**) (DA)_2_MnCl_4_ and (**d**) (NA)_2_CuBr_4_ up to 500 bar, with heating and cooling rates of 0.5 K min^–1^. Note that He was used as the pressure-transmitting medium. Isothermal entropy changes (Δ*S*_it_) calculated by the quasi-direct method for (**b**) (DA)_2_MnCl_4_ and (**e**) (NA)_2_CuBr_4_. The shaded area indicates the reversible Δ*S*_it_ within this pressure range. Maximum reversible isothermal entropy change (Δ*S*_it,rev,max_) and reversible refrigeration capacity (RC_rev_) for (**c**) (DA)_2_MnCl_4_ and (**f**) (NA)_2_CuBr_4_ as a function of operating pressure, with the minimum pressures required to induce a reversible isothermal entropy change (*P*_rev_) and a reversible adiabatic temperature change (*P*_rev,ad_) marked using vertical lines. Note that Δ*S*_it,rev,max_ is equivalent to the peak value of each reversible isothermal entropy curve, and RC_rev_ is calculated as Δ*S*_it,rev,max_ × Δ*T*_FWHM_. The dependence of RC_rev_ on operating pressure above *P*_rev,ad_ is highlighted using blue dashed lines. The pressure dependence of RC_rev_ is 4524 J kbar^–1^ kg^–1^ and 1830 J kbar^–1^ kg^–1^ for (DA)_2_MnCl_4_ and (NA)_2_CuBr_4_, respectively.

**Fig. 6:**
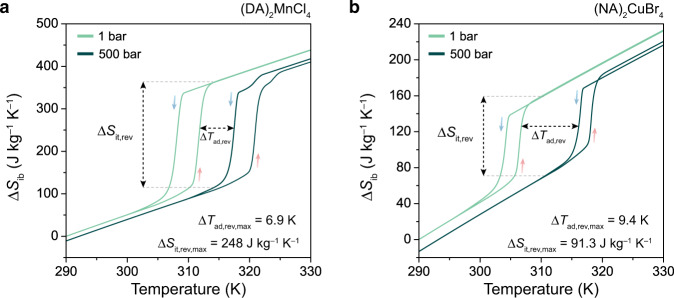
Reversible barocaloric effects at 500 bar. Isobaric entropy changes (Δ*S*_ib_) during heating and cooling at ambient pressure and 500 bar are shown for (**a**) (DA)_2_MnCl_4_ and (**b**) (NA)_2_CuBr_4_. Note that the Δ*S*_ib_ curves include contributions from the heat capacity. The area between Δ*S*_ib_(*T*, 1 bar)_heating_ and Δ*S*_ib_(*T*, 500 bar)_cooling_ curves denotes the temperature range over which both reversible isothermal entropy changes (Δ*S*_it,rev_) and reversible adiabatic temperature changes (Δ*T*_ad,rev_) are accessible.

In order to evaluate reversible adiabatic temperature changes, we performed additional HP-DSC experiments at a higher pressure range of 300–500 bar for a powder sample of (DA)_2_MnCl_4_ (Fig. 5a–c). This data shows that the order–disorder phase transition persists to at least 500 bar of hydrostatic pressure while maintaining 94% of the ambient pressure entropy change (Supplementary Fig. 20). At a 500-bar operating pressure, (DA)_2_MnCl_4_ has a maximum reversible isothermal entropy change, Δ*S*_it,rev,max_, of 248 kg^–1^ K^–1^ and a maximum reversible adiabatic temperature change, Δ*T*_ad,rev,max_, of 7 K (Fig. 6a, Supplementary Fig. 22). We note that the single order–disorder phase transition splits into a major transition (93% of the total entropy change) and a minor phase transition (7% of the total entropy change) at pressures above 400 bar, with the minor transition occurring at a slightly higher temperature than the major transition (Fig. 5a, Supplementary Fig. 8a). This is consistent with similar splitting—albeit at much higher pressures—and entropy changes that were recently reported in an independent study of the barocaloric properties of (DA)_2_MnCl_4_ at pressures above 300 bar while this manuscript was under peer review^25^.

Although quasi-direct methods of calculating isothermal changes—and adiabatic changes—from isobaric experiments are commonly used to evaluate barocaloric materials due to the challenge of maintaining isothermality—or adiabaticity—during direct variable-pressure measurements^33^, we also performed quasi-isothermal HP-DSC experiments to more directly evaluate *P*_rev_ by measuring pressure hysteresis. Specifically, we measured heat flow signals over three cycles of increasing and decreasing hydrostatic pressure between 1 bar and 150 bar at 311 K. By comparing the onset pressures for compression-induced exotherms and decompression-induced endotherms, we were able to directly measure a pressure hysteresis of 62 ± 4 bar for (DA)_2_MnCl_4_, which is in excellent agreement with the predicted value of 70 bar at 311 K (Fig. 3c, Supplementary Fig. 18, Supplementary Table 11).

### Reversible barocaloric effects in (NA)_2_CuBr_4_

In an effort to target barocaloric materials with large reversible entropy and adiabatic temperature changes at even lower pressures, we searched for a 2-D perovskite that undergoes a hydrocarbon order–disorder transition near ambient temperature with a thermal hysteresis of less than 1 K. Unlike (DA)_2_MnCl_4_, however, the total entropy of the order–disorder transition in most 2-D perovskites is partitioned across one or more minor—lower entropy—phase transitions in addition to the principal transition (Fig. 1b, Supplementary Tables 1–3). Although not necessarily detrimental to barocaloric cooling performance^34^, the presence of multiple successive transitions at different temperatures complicates the evaluation of barocaloric properties because each minor and major transition has an independent hysteresis loop—often greater than 1 K—with different pressure dependences (Supplementary Table 4). With a lack of suitable existing compounds, we endeavored to synthesize a new 2-D perovskite that features a sharp order–disorder transition near ambient temperature with ultralow hysteresis and a high sensitivity to pressure.

After screening different combinations of metal cations, halide anions, and alkylammonium chain lengths, we discovered a 2-D perovskite (NA)_2_CuBr_4_ (NA = nonylammonium) that undergoes an order–disorder transition at 305 K with a high Δ*S*_tr_ (76 J kg^–1^ K^–1^) and hysteresis of only 0.4 K for a powder sample (Fig. 2b) and 1.0 K for a single-crystal sample (Fig. 4e). Variable-temperature PXRD experiments, combined with single-crystal structures at 270 K and 335 K, revealed that the phase transition involves a 4.0% increase in volume (Δ*V*_tr_ = 25.3 cm^3^ kg^−1^) and a 2.9% (0.67 Å) increase in interlayer distance (Fig. 2d, f; Supplementary Figs. 1 and 4). The crystallographic volume change yields a predicted barocaloric coefficient of 33.3 K kbar^−1^, while the volume change determined by He pycnometry (Δ*V*_tr_ = 20.0 cm^3^ kg^–1^)—which accounts for He permeation—yields a predicted barocaloric coefficient of 26.3 ± 2.5 K kbar^–1^ (Supplementary Table 10).

**Fig. 7:**
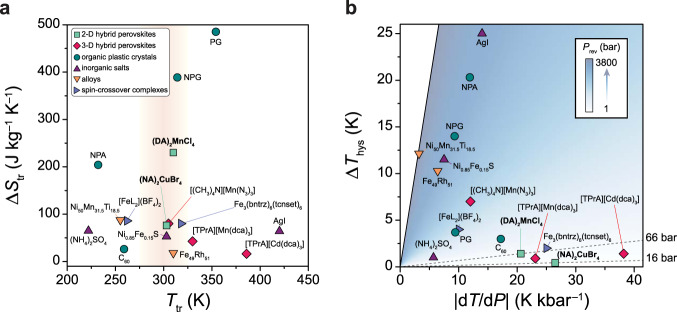
Properties of representative barocaloric materials. **a** Comparison of the phase-change entropy, Δ*S*_tr_, and temperature, *T*_tr_, for different classes of barocaloric materials. Note that Δ*S*_tr_ and *T*_tr_ are shown for endothermic transitions, and Δ*S*_tr_ represents the maximum isothermal entropy change that could be driven by the pressure-induced phase transition. **b** Comparison of thermal hysteresis, Δ*T*_hys_, and barocaloric coefficient, d*T*/d*P*, for leading barocaloric materials, with d*T*/d*P* values corresponding to exothermic transitions for materials that exhibit conventional barocaloric effects and endothermic transitions for materials that exhibit inverse barocaloric effects. The minimum pressure required to achieve a reversible entropy change, *P*_rev_, is calculated as *P*_rev_ = Δ*T*_hys_/|d*T*/d*P*| and indicated by shading from blue (high *P*_rev_) to white (low *P*_rev_). A comprehensive tabulation of barocaloric properties, including reversible and irreversible Δ*S*_it_ values, is provided in Supplementary Tables 5 and 6.

Isobaric HP-DSC experiments confirmed that (NA)_2_CuBr_4_ features high barocaloric coefficients of 26.7 ± 0.4 K kbar^–1^ during both heating and cooling (Fig. 3d, j; Supplementary Fig. 11, Supplementary Table 8). In fact, these values represent one of the highest sets of barocaloric coefficients ever measured (Supplementary Table 5). As a result of its high barocaloric coefficient, ultralow hysteresis, and extremely sharp transition, the powder sample of (NA)_2_CuBr_4_ has, to the best of our knowledge, the lowest reported *P*_rev_ for a barocaloric material of 16 bar (Fig. 7b), which is within the pressure range already accessed during commercial vapor-compression refrigeration cycles^19^. The low value of *P*_rev_ was further confirmed through quasi-isothermal pressure cycling experiments at 306 K, where we directly measured a pressure hysteresis of 18 bar (Fig. 3f, Supplementary Fig. 19). Moreover, a large reversible entropy change, with a maximum value of 68 J kg^−1^ K^−1^ (90% of Δ*S*_tr_ at 1 bar), is accessible over the temperature range of 2 K at an operation pressure of 150 bar for the powder sample (Supplementary Fig. 16c, d). Through additional HP-DSC experiments conducted at higher pressure ranges up to 500 bar, we demonstrated that a large reversible entropy change of 78 J kg^−1^ K^−1^—equivalent to the full transition entropy—is accessible over a wider temperature range of 5 K and 10 K at 300 bar and 500 bar, respectively (Figs. 5d–f, 6b; Supplementary Figs. 21 and 22). Note that the order–disorder transition over this extended pressure range is associated with a similar volume change (21–25 cm^3^ kg^–1^) as revealed by PXRD experiments (Supplementary Figs. 4, 26–29; Supplementary Table 12), and thus maintains a high sensitivity to pressure of 24 K kbar^−1^ (Fig. 3i, j; Supplementary Fig. 7; Supplementary Table 8).

### Impact of thermal contact on low-pressure reversibility

Our results for single-crystal samples of (DA)_2_MnCl_4_ and (NA)_2_CuBr_4_ show how improved thermal contact can lead to even larger reversible barocaloric effects at lower pressures (Fig. 4, Supplementary Fig. 17). For instance, at a 150-bar driving pressure, the maximum value of Δ*S*_it,rev_ increases from 75 J kg^−1^ K^−1^ (powder with poor thermal contact) to 190 J kg^−1^ K^−1^ (single crystal with improved thermal contact) for (DA)_2_MnCl_4_ and from 68 J kg^−1^ K^−1^ (powder) to 78 J kg^−1^ K^−1^ (single crystal) for (NA)_2_CuBr_4_ (Supplementary Table 7). At even lower pressures, (NA)_2_CuBr_4_ features a reversible isothermal entropy change of 4 J kg^−1^ K^−1^ and 11 J kg^−1^ K^−1^ at 20 bar and 40 bar, respectively, for a powder sample (Fig. 5f, Supplementary Fig. 16d) and of 5 J kg^−1^ K^−1^ and 49 J kg^−1^ K^−1^ at 40 and 60 bar, respectively, for a single-crystal sample (Fig. 3e, Supplementary Fig. 17f). Notably, the single-crystal sample of (NA)_2_CuBr_4_ exhibits a reversible adiabatic temperature change (Δ*T*_ad,rev_) of 2.3 K at a 150-bar driving pressure (Supplementary Fig. 17g), which represents, to the best of our knowledge, the first demonstration of Δ*T*_ad,rev_ of a sizable magnitude—sufficient to create much wider temperature spans through regeneration^35^—near room temperature at a more practically accessible pressure for a barocaloric material.

### Relationships between entropy changes and reversibility

**Fig. 8:**
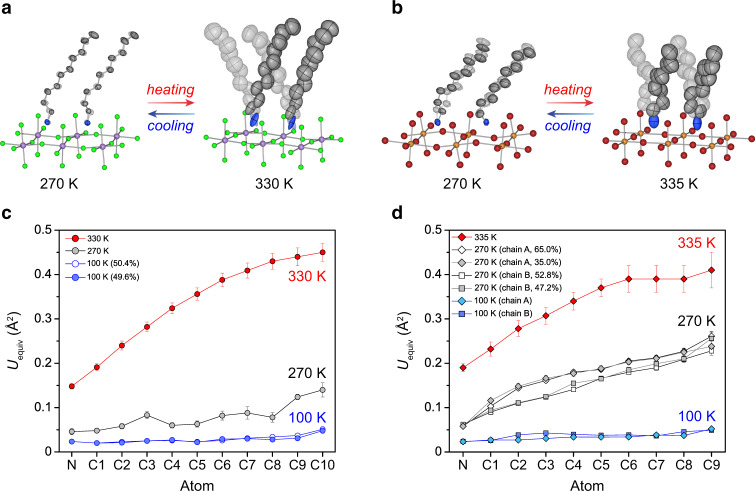
Variable-temperature single-crystal structures. Conformations of the alkylammonium chains in the LT and HT phases of (**a**) (DA)_2_MnCl_4_ and (**b**) (NA)_2_CuBr_4_, with atomic displacement parameters shown at 50% probability for the C and N atoms of the alkylammonium chains. In the LT phase, decylammonium (DA) chains in (DA)_2_MnCl_4_ display one conformation, with a single gauche C–C bond (C2–C3), while nonylammonium (NA) chains in (NA)_2_CuBr_4_ adopt two conformations, alternating between chains with a gauche C1–C2 bond (chain A) and those with a gauche C2–C3 bond (chain B). Purple, orange, green, brown, gray, and blue spheres represent Mn, Cu, Cl, Br, C, and N atoms, respectively. H atoms are omitted for clarity. Note that DA chains are disordered over a special position in both the LT and HT phases, while NA chains are modeled with two-part disorder in the LT phase and disordered over a special position in the HT phase. Temperature dependence of *U*_equiv_ (equivalent isotropic displacement parameters) is shown for alkylammonium cations in (**c**) (DA)_2_MnCl_4_ and (**d**) (NA)_2_CuBr_4_, at 100 K, 270 K (LT phase), and 330/335 K (HT phase). Error bars represent standard uncertainties.

To provide additional insight into the structural and chemical factors that influence barocaloric effects in 2-D perovskites, we used X-ray crystallography and infrared (IR) spectroscopy to compare the nature of the hydrocarbon order–disorder transition in (NA)_2_CuBr_4_ and (DA)_2_MnCl_4_. In particular, we hypothesized that the increased size of the halide pocket in (NA)_2_CuBr_4_ (30.5 Å^2^) relative to (DA)_2_MnCl_4_ (26.3 Å^2^)—coupled with weaker N−H⋅⋅⋅Br hydrogen bonds at the organic–inorganic interface—would lead to increased free volume in the organic bilayer of the Cu compound (Supplementary Figs. 34 and 35) and, as a result, greater disorder in the LT phase. As anticipated, the atoms in the NA chains of (NA)_2_CuBr_4_ have much larger atomic displacement parameters in the LT phase than those in the DA chains of the LT phase of (DA)_2_MnCl_4_ (Fig. 8). This is consistent with the LT phase of (NA)_2_CuBr_4_ having increased vibrational and conformational degrees of freedom, which would reduce the entropy difference between the LT and HT phases (Supplementary Notes 1 and 2).

The increased entropy of the alkylammonium chains of (NA)_2_CuBr_4_ prior to the phase transition is further corroborated by a broad feature in the heat capacity from 220–250 K that is consistent with a gradual activation of conformational degrees of freedom from the fully ordered organic bilayer that is present at 100 K (Supplementary Figs. 32 and 36). Variable-temperature IR spectra are also consistent with increased conformational entropy for the hydrocarbon chains in the LT phase of (NA)_2_CuBr_4_. Specifically, IR spectra show a band near 1360 cm^–1^, which can be assigned to CH_2_ wagging from *gt*_2*n*+1_*g*′-type kinks, that is present below the phase transition temperature for (NA)_2_CuBr_4_ but only above the phase transition temperature for (DA)_2_MnCl_4_ (Supplementary Fig. 37). As discussed in greater detail in Supplementary Note 6, the IR spectra also suggest that the local environment around the chain ends (CH_3_) and headgroups (NH_3_^+^) is more similar in the LT and HT phases of (NA)_2_CuBr_4_ than in those of (DA)_2_MnCl_4_ (Supplementary Table 13).

Although increasing the halide pocket size through Br substitution leads to a decreased entropy change (Fig. 7a), it also likely contributes to the enhanced reversibility of the (NA)_2_CuBr_4_ phase transition through two primary effects. First, the softer nature of Br anions—along with the higher degree of disorder in the alkylammonium chains in the LT phase—makes the (NA)_2_CuBr_4_ lattice more compressible than the (DA)_2_MnCl_4_ lattice (Supplementary Table 9). Since the barocaloric coefficient d*T*/d*P* of a solid tends to increase with increasing compressibility^12,36^, this causes the phase transition in (NA)_2_CuBr_4_ to be more sensitive to pressure, which reduces the minimum pressure required to overcome hysteresis and induce a reversible phase transition. Indeed, d*T*/d*P* for the ordering transition in (NA)_2_CuBr_4_ is 29% higher than for (DA)_2_MnCl_4_ (Supplementary Table 7). Second, the increased free volume and presence of additional vibrational and conformational degrees of freedom in both the LT and HT phases should render the two phases more compatible, reducing the activation energy barrier for nucleation of a more ordered bilayer phase during cooling—or compression—and lowering both the isobaric and isothermal hysteresis^37^.

### Impact of hysteresis on thermodynamic efficiency

In addition to its influence on operating pressure, hysteresis adversely impacts the second-law efficiency and coefficient of performance (COP) of any caloric cooling cycle because of dissipative heat losses^16,17^. The impact of hysteresis on efficiency can be quantified by calculating an idealized thermodynamic efficiency, *η*—relative to the Carnot efficiency—with a simple material model that accounts for dissipative losses due to hysteresis in a Carnot-like cycle^16^:

1 $$\eta =\frac{{{{{{\rm{COP}}}}}}}{{{{{{{\rm{COP}}}}}}}_{{{{{{\rm{Carnot}}}}}}}}=\frac{1}{1+4\frac{\Delta {T}_{{{{{{\rm{hys}}}}}}}}{\Delta {T}_{{{{{{\rm{ad}}}}}},{{{{{\rm{max }}}}}}}}}$$

Based on this model, caloric materials with Δ*T*_hys_/Δ*T*_ad,max_ of less than 10% will have idealized second-law efficiencies competitive with those of conventional vapor compression-based systems (~85%)^38^. Notably, (DA)_2_MnCl_4_ and (NA)_2_CuBr_4_ have the potential to reach the second-law efficiencies of 82 and 79%, respectively, based on their intrinsic barocaloric properties (Supplementary Table 6). Further, both compounds display very large reversible refrigeration capacities (RC_rev_) under easily accessible pressures (Fig. 5c, f).

## Discussion

**Fig. 9:**
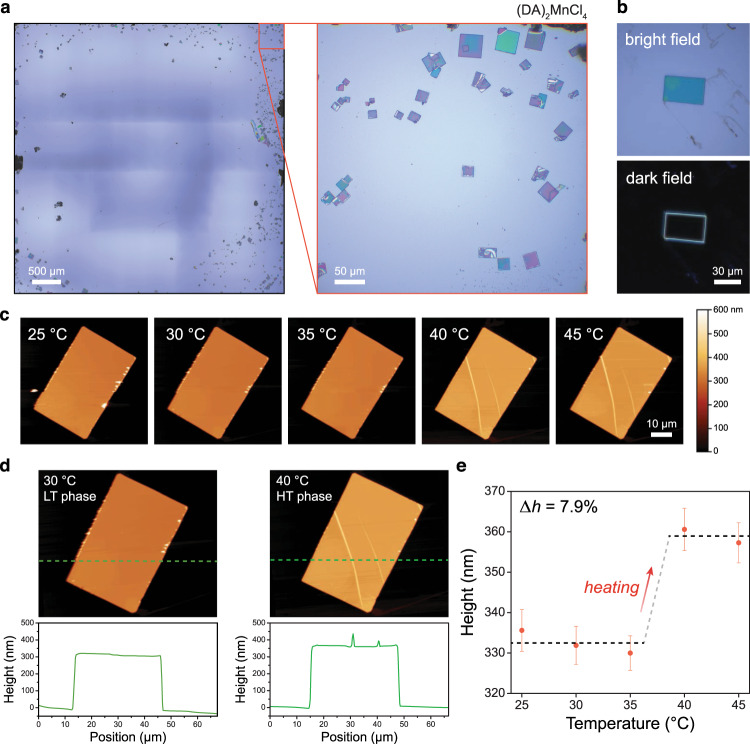
Phase transition of single crystals on a substrate. **a** Optical images of (DA)_2_MnCl_4_ single crystals directly grown on Si substrates through an anti-solvent vapor-assisted capping crystallization method. **b** Bright field (top) and dark field (bottom) images of a sub-micron thick single crystal. **c** Variable-temperature atomic force microscope (AFM) imaging experiments for a sub-micron thick (DA)_2_MnCl_4_ single crystal. **d** Height profiles of a sub-micron thick (DA)_2_MnCl_4_ single crystal in the low-temperature (LT, left) and high-temperature (HT, right) phases. **e** Thickness of a single crystal of (DA)_2_MnCl_4_ as a function of temperature. The heating-induced transition from the LT phase to HT phase gives rise to an 7.9% change in height, which agrees well with the 7.4% predicted from crystallographic data obtained from variable-temperature PXRD experiments at 30 °C and 45 °C. Error bars were obtained from the mean square difference between all data points in the scan region and the fitted step height. Thermal cycling AFM experiments are described in Supplementary Fig. 43.

Overall, these results highlight exciting opportunities to exploit the tunability of 2-D perovskites to independently manipulate the hysteresis, sharpness, entropy, and sensitivity to pressure of hydrocarbon order–disorder phase transitions for improved barocaloric performance. For instance, it should be possible to realize phase transitions with even higher entropy changes through functionalization of the organic bilayers—such as by introducing hydrogen bond donor–acceptor pairs^39,40^—and even lower hysteresis through modification of the organic–inorganic interface—such as by incorporating mixtures of different halide anions or introducing defects. Further, the modular nature and high tunability of 2-D perovskites can be leveraged to optimize, in addition to intrinsic barocaloric properties, a host of factors that are critical to realizing scalable and efficient solid-state cooling technologies, including safety, flammability, and thermal conductivity (Supplementary Figs. 39–42). In addition, the anisotropic nature of the order–disorder transition in (DA)_2_MnCl_4_ and (NA)_2_CuBr_4_—as highlighted by variable-temperature atomic force microscopy (AFM) experiments on single crystals directly grown on a Si wafer (Fig. 9, Supplementary Fig. 43)—suggests that uniaxial stress, which can be readily applied through mechanical actuation, may be able to drive large elastocaloric effects in 2-D perovskites and that barocaloric effects should be accessible in 2-D perovskites with sub-micron thickness. More broadly, solid-state hydrocarbon order–disorder transitions are not unique to 2-D perovskites, and many other classes of layered materials—including di-*n*-alkylammonium salts^41^, alkylammonium-modified layered silicates^42^, and metal-alkylphosphonate salts^43^—undergo reversible phase transitions near ambient temperature that should produce colossal barocaloric effects and have yet to be explored.

## Methods

### Materials

All compounds were synthesized and handled in air unless otherwise noted. Anhydrous diethyl ether was obtained from a Pure Process Technology anhydrous solvent system. Anhydrous methanol and ethanol were purchased from a commercial vendor and used as received. All other reagents were purchased from commercial vendors and used as received.

### Synthesis of (DA)_2_MnCl_4_ (DA = decylammonium, C_10_H_21_NH_3_)

Decylamine (≥99.0% purity) and hydrochloric acid (HCl, 37 wt %) were purchased from Sigma Aldrich and used without further purification. Decylammonium chloride (C_10_H_21_NH_3_Cl, (DA)Cl) was first synthesized by adding HCl (550 μL, 6.6 mmol) into a solution of decylamine (1.1 mL, 5.5 mmol) in ca. 5 mL ethanol in an ice water bath with stirring. After evaporating the solvent under reduced pressure, the resulting white powder of (DA)Cl was washed with diethyl ether and dried under vacuum at room temperature for 1 day. Crystalline powders of (DA)_2_MnCl_4_ were prepared in a similar manner to previously reported syntheses^26,44^. Specifically, (DA)Cl (96.9 mg, 0.5 mmol) was first dissolved in 4.0 mL of ethanol. After several minutes of stirring, MnCl_2_·4H_2_O (49.5 mg, 0.25 mmol) was added to the solution, and the solution was heated to 65 °C. Pale-pink crystals were obtained upon cooling the resulting solution to room temperature at a rate of 4 K h^−1^. The crystals were filtered, washed with diethyl ether (5 × 10 mL), and dried under vacuum for 6 h to afford 45.2 mg (35.2% yield) of product. Single crystals suitable for X-ray structure determination were obtained by cooling at a rate of 2 K h^−1^.

### Synthesis of (NA)_2_CuBr_4_ (NA = decylammonium, C_9_H_19_NH_3_)

Nonylamine (≥99.5% purity) and hydrobromic acid (HBr, 48 wt %) were purchased from Sigma Aldrich and used without further purification. Nonylammonium bromide (C_9_H_19_NH_3_Br, (NA)Br) was first synthesized by adding HBr (545 μL, 4.8 mmol) into a solution of nonylamine (733 μL, 4.0 mmol) in ca. 5 mL ethanol in an ice water bath with stirring. After evaporating the solvent under reduced pressure, the resulting white powder of (NA)Br was washed with diethyl ether and dried under vacuum at room temperature for 1 day. Crystalline powders of (NA)_2_CuBr_4_ were prepared by dissolving of CuBr_2_ (402 mg, 1.8 mmol) and (NA)Br (807 mg, 3.6 mol) in 2 mL of ethanol at 65 °C. The solution was slowly cooled to room temperature at a rate of 4 K h^−1^ and then further cooled to 5 °C for 1 h. The resulting dark purple precipitate was filtered, washed with diethyl ether (5 × 10 mL), and dried under vacuum for 12 h to afford 705 mg (58.3% yield) of product. Single crystals suitable for X-ray structure determination were obtained by slow evaporation of a 1-mL solution of (NA)_2_CuBr_4_ (202 mg, 0.3 mmol) in methanol. Anal. Calcd. for (C_9_H_19_NH_3_)_2_CuBr_4_: C: 32.19%, H: 6.60%, N: 4.17%, Br: 47.58%. Found: C: 31.84%, H: 6.69%, N: 4.43%, Br: 47.76%.

### Growth and characterization of isolated single crystals on substrates

Single crystals of (DA)_2_MnCl_4_ were grown onto p-type <100> Si substrates based on an anti-solvent vapor-assisted capping crystallization (AVCC) method^45^. Specifically, crystals of (DA)_2_MnCl_4_ were dissolved at a concentration of 100 mM in a 1:1 solution of ethanol and methanol, which was then pipetted onto plasma-cleaned Si substrates and immediately capped with polished *c*-plane sapphire discs. The capped substrates were placed alongside vials of diethyl ether in a closed jar and allowed to dry over several days. Optical images of crystals were taken using a Motic Panthera TEC microscope. Thickness and roughness of crystals grown by the AVCC method were measured using an atomic force microscope (Jupiter XR, Asylum Research, Santa Barbara, CA, USA) in tapping mode. The step height was calculated in the Gwyddion software by fitting the scanned region with a piecewise function over the crystal surface and the substrate. Variable temperature measurements were performed using the PolyHeater accessory.

### Thermogravimetric analysis

Samples were loaded into a TGA 550 from TA Instruments in open aluminum pans with a stainless-steel bail under air and heated at a rate of 4 K min^–1^ from ambient to 500 °C under a 10 mL min^–1^ N_2_ flow with an empty aluminum pan/stainless-steel bail used as a reference counterweight. The TGA mass was calibrated using a series of 3 reference masses, while the TGA temperature was calibrated to the Curie temperature of nickel. The thermogravimetric analyses of (DA)_2_MnCl_4_ and (NA)_2_CuBr_4_ are shown in Supplementary Figs. 39 and 40.

### High-pressure differential scanning calorimetry (HP-DSC)

DSC measurements over the pressure range from 1 to 150 bar were carried out in a Netzsch high-pressure DSC (DSC 204 HP Phoenix) equipped with a liquid nitrogen cooling system. The DSC sample cell is surrounded by an autoclave, where the internal pressure and gas flow are regulated by an electronic pressure control device. The autoclave is connected to a circulating water bath that provides an additional source of external temperature control during experiments. Temperature and heat flow signals were calibrated at each measured pressure using an indium standard^46^. Helium gas (ultra-high purity, 99.999%) was used as a pressure-transmitting medium. All DSC samples were prepared in air. For powder samples, 5–10 mg of loosely packed ground single crystals were sealed in an aluminum pan with a pierced lid. For single-crystal samples, a large single-crystal flake was lightly pressed onto the bottom of an aluminum pan, which was then sealed with a pierced lid. An empty, aluminum pan with a pierced lid was used as a reference. All measurements were carried out under flowing gas with a He flow rate of 50 mL min^–1^. Unless otherwise noted, heating and cooling rates of 2 K min^−1^ were used for isobaric measurements.

DSC measurements over the pressure range from 300 to 500 bar were conducted in a Setaram high-pressure DSC (Microcalvet) using microcrystalline powder samples. The calorimeter was calibrated based on the Joule effect, which does not require reference materials. For each set of measurements, 7 mg of sample was encapsulated in the high-pressure vessel of the calorimeter, and the pressure was generated by a compressing piston using Helium gas as a pressure-transmitting medium.

### Determination of *T*_tr_, Δ*H*_tr_, and Δ*S*_tr_

Phase transition temperatures, *T*_tr_, and enthalpies, Δ*H*_tr_, were determined using the TRIOS software from TA Instruments. Peaks were selected for analysis by defining a temperature range containing the peak of interest. The lower and upper bounds of the temperature range were chosen to encompass the phase transition, which starts with a deviation from the baseline and ends with a return to baseline. Prior to determination of *T*_tr_ or Δ*H*_tr_, a baseline, which models the heat flow in the absence of a phase transition, must be generated to approximate the baseline in the transition region. Baselines were generated within the defined temperature range to determine the slope of the lower and higher temperature limits and shape of the baseline. Baselines were generated using mutual tangent slopes before and after the transition peak with a sigmoidal baseline, which we found to produce the most physically reasonable baselines. The extrapolated onset temperature was reported as the transition temperature, as is standard in DSC data analysis, because the onset temperature—unlike the peak temperature—is relatively independent of experimental parameters like heating rate or sample mass. The onset temperature was determined by identifying the region of the onset transition peak that has the steepest slope, defining a tangent to that region, and then extending the tangent to the generated baseline. The intersection between the baseline and the tangent is reported as *T*_tr_. Transition peaks were integrated between the upper and lower temperature limits with the baseline subtracted to provide ∆*H*_tr_, and phase transition entropies, ∆*S*_tr_, were calculated as ∆*S*_tr_ = ∆*H*_tr_/*T*_tr_. If physically reasonable limits were chosen, *T*_tr_ and ∆*H*_tr_ did not depend strongly on the choice of the temperature limits, and such variations were within the error of the measurements, which is estimated to be ± 0.04 °C for *T*_tr_ and <3% for ∆*H*_tr_ based on repeated measurements of the melting transition of an indium standard.

### Construction of isobaric entropy curves

Isobaric entropy changes, Δ*S*_ib_, were calculated as a function of temperature and pressure by integrating the HP-DSC heat flow signal *Q*, obtained at a scan rate of $\dot{T}$ near the transition peak over the temperature range from *T*_i_ to *T*_f_ after baseline subtraction^33,47^:

2 $$\Delta {S}_{{{{{{\rm{ib}}}}}}}=\int _{{T}_{{{\mathrm{i}}}}}^{{T}_{{{\mathrm{f}}}}}\frac{1}{T}\frac{Q(T,P)}{\dot{T}}{{{{{\rm{d}}}}T}}$$

In a typical isobaric measurement with a scan rate of 2 K min^−1^ at pressures up to 150 bar, the temperature range for peak integration, Δ*T*_int_ = *T*_f_ – *T*_i_, was set to 16 K for microcrystalline powder samples, for consistency, with *T*_i_ = *T*_tr_ − 8 K and *T*_f_ = *T*_tr_ + 8 K. For single-crystal samples, due to their sharper transition peak width, a narrower temperature range was chosen, with Δ*T*_int_ of 5 K and 2 K for (DA)_2_MnCl_4_ and (NA)_2_CuBr_4_, respectively. For isobaric measurements with a scan rate of 0.5 K min^−1^ over the pressure range of 300 to 500 bar, Δ*T*_int_ of 12 K and 10 K were chosen for (DA)_2_MnCl_4_ and (NA)_2_CuBr_4_, respectively. Baselines for peak integration were generated using mutual tangent slopes, and the tangents before and after the transition peak were created from *T*_i_ – 4 K to *T*_i_ and from *T*_f_ to *T*_f_ + 4 K, respectively. Tangent slopes were created over this extended temperature range to minimize any uncertainty in integration, particularly at higher gas pressures which lead to more noisy baselines. Since the integration was carried out near the transition without taking into account the heat capacity of each phase to emphasize the contributions from the order–disorder transition, the isobaric entropy curves are flat before and after the phase transition region (Supplementary Figs. 10, 11, 14, 15, 20, 21).

A positive slope in the isobaric entropy curves was introduced by adding the entropy contribution from heat capacity [*S*_c_(*T*)], calculated through Eq. (3), to the isobaric entropy changes associated with the phase transition.

3 $${S}_{c}(T)=\left\{\begin{array}{c}{S}_{c}({T}_{0})+\int _{{T}_{0}}^{T}\frac{{c}_{P}^{{{{{{\rm{LT}}}}}}}}{T^{\prime}}{{{{\mathrm{d}}}}T^{\prime}} \kern1pc {{T}}_{0}\le T\le {T}_{{{\mathrm{i}}}}\\ {S}_{c}({T}_{{{\mathrm{i}}}})+\int _{{T}_{{{\mathrm{i}}}}}^{T}\frac{{c}_{P}}{{T}^{\prime}}{{{{\mathrm{d}}}}T^{\prime}}\kern1.4pc{{T}}_{{{{{{\rm{i}}}}}}}\le T\le \,{T}_{{{\mathrm{f}}}}\\ \kern-1.7pc {S}_{c}({T}_{{{\mathrm{f}}}})+\int _{{T}_{{{\mathrm{f}}}}}^{T}\frac{{c}_{P}^{{{{{{\rm{HT}}}}}}}}{T^{\prime}}{{{{\mathrm{d}}}}T^{\prime}}\kern1pc {T}_{{{{{{\rm{f}}}}}}}\le T\end{array}\right.$$

For both compounds, the reference temperature *T*_0_ was set to 290 K, with *S*_c_(*T*_0_) = 0. The heat capacity values during the phase transition (between *T*_i_ and *T*_f_) were estimated through ${c}_{p}\,=\,(1-x){c}_{P}^{{{{{{\rm{LT}}}}}}}\,+\,x{c}_{P}^{{{{{{\rm{HT}}}}}}}$ where *x* is the phase fraction of the material in the HT phase at the temperature *T*.

The dependence of the heat capacity contribution *S*_c_(*T*) on applied pressure can be estimated by calculating the pressure-induced changes in isothermal entropy that arises outside of the phase transition region. This entropy contribution, Δ*S*_+_, is often referred to as the elastic heating contribution^4,48^ and can be calculated using the following formula derived from the Maxwell relation (∂*V*/∂*T*)_*P*_ = −(∂*S*/∂*P*)_*T*_:

4 $$\Delta {S}_{+}({P}_{{{\mathrm{f}}}}-{P}_{{{\mathrm{i}}}})=-\int _{{P}_{{{\mathrm{i}}}}}^{{P}_{{{\mathrm{f}}}}}\left(\frac{\partial V}{\partial T}\right){{{\mathrm{d}}}}P$$

For normal barocaloric materials with (∂*V*/∂*T*)_*P*_ > 0, this entropy contribution originates from the applied pressure counteracting entropy increases due to thermal fluctuations. By assuming that the thermal expansion coefficient is independent of pressure, Δ*S*_+_ can be estimated as

5 $$\Delta {S}_{+}=-[{(\partial V/\partial T)}_{P=0}]\Delta P=-(V\cdot \alpha )\cdot \Delta P$$

where *V*, Δ*P*, and *α* denote the specific volume, driving pressure Δ*P* = *P*_f_ − *P*_i_, and thermal expansion coefficient at ambient pressure, respectively, with *α* = *V*^−1^(∂*V*/∂*T*)_*P*=0_. Equation (5) is commonly used to estimate Δ*S*_+_ since high-pressure heat capacity measurements are difficult to perform accurately. At a given driving pressure Δ*P*, isobaric entropy curves that include the contributions from heat capacity were obtained by adding *S*_c_(*T*) to Δ*S*_ib_ and shifting the curves downward by Δ*S*_+_ of the LT phase.

Although both (DA)_2_MnCl_4_ and (NA)_2_CuBr_4_ display large thermal expansion coefficients on the order of 10^−4^ K^−1^, their estimated Δ*S*_+_ values are small relative to their transition entropies at pressures up to 150 bar. For example, for a driving pressure of 150 bar, Δ*S*_+_ in the LT phases of (DA)_2_MnCl_4_ and (NA)_2_CuBr_4_ is predicted to be just −3 J kg^−1^ K^−1^ and −4 J kg^−1^ K^−1^, respectively. We note, however, that Δ*S*_+_ can become significant at higher driving pressures and can further enhance the barocaloric effect. The calculated Δ*S*_+_ values are summarized in Supplementary Table 9.

### Evaluation of isothermal entropy changes

Pressure-induced isothermal entropy changes, Δ*S*_it_, were calculated by the quasi-direct method^33^ as the difference between Δ*S*_ib_ at ambient pressure *P*_0_ of 1 bar, Δ*S*_ib_(*T*, *P*_0_), and Δ*S*_ib_ at elevated pressure, Δ*S*_ib_(*T*, *P*). For Δ*S*_it_ corresponding to the endothermic, disordering transition induced by decompression (*P* → *P*_0_), Δ*S*_ib_ values were obtained from heating data:

6 $$\Delta {S}_{{{{{{\rm{it}}}}}}}(P\to {P}_{0})=\Delta {S}_{{{{{{\rm{ib}}}}}},{{{{{\rm{heating}}}}}}}(T,{P}_{0}){{\mbox{ - }}}\Delta {S}_{{{{{{\rm{ib}}}}}},{{{{{\rm{heating}}}}}}}(T,P)$$

For Δ*S*_it_ corresponding to the exothermic, ordering transition induced by compression (*P*_0_ → *P*), Δ*S*_ib_ values were obtained from cooling data:

7 $$\Delta {S}_{{{{{{\rm{it}}}}}}}({P}_{0}\to P)=\Delta {S}_{{{{{{\rm{ib}}}}}},{{{{{\rm{cooling}}}}}}}(T,P){{\mbox{ - }}}\Delta {S}_{{{{{{\rm{ib}}}}}},{{{{{\rm{cooling}}}}}}}(T,{P}_{0})$$

For operating pressures above *P*_rev_, the reversible values of Δ*S*_it_ were estimated from the overlap between compression-induced and decompression-induced Δ*S*_it_ curves reflected across the temperature axis. From the reversible isothermal entropy curves, the reversible refrigeration capacity values, RC_rev_, were calculated as Δ*S*_it,rev,max_ × Δ*T*_FWHM_, where Δ*S*_it,rev,max_ is the maximum reversible isothermal entropy change for a given pressure and Δ*T*_FWHM_ is the full width at half maximum of the reversible isothermal entropy peak at a given pressure (Fig. 5c, f).

### Evaluation of adiabatic temperature changes

For evaluations of adiabatic temperature changes, two different methods were used: (*i*) the indirect method and (*ii*) the quasi-direct method. The indirect method is based on the simple relationship Δ*T*_ad,indirect_ = –*T*Δ*S*_it_/*c*_p_ (for specific values used for the calculation, see Supplementary Table 6). Although the indirect method is a useful way of evaluating an upper bound for the magnitude of an adiabatic temperature that could be achieved with a given material, this approach is not appropriate for calculating the dependence of Δ*T*_ad_ on operating pressure or the magnitude of Δ*T*_ad_ that can be accessed under reversible conditions. Moreover, the indirect method is accurate only when *c*_p_ is relatively independent of pressure and Δ*T*_ad,indirect_ is sufficiently smaller than *T* (ref. ^33^.).

The magnitude of the adiabatic temperature change that can be accessed for a given pressure shift, however, can be evaluated through the so-called quasi-direct method by constructing two isobaric curves on a temperature (*T*) vs. entropy (*S*) plot at two different pressures (typically ambient pressure, *P*_0_, and applied pressure, *P*) (refs. ^11,33^). In such a plot, the width of horizontal—and adiabatic—lines between two isobaric entropy curves corresponds to the magnitude of the adiabatic temperature change that will be accessible for a given change in pressure. For the adiabatic temperature change that is induced by the first pressure change (decompression-induced cooling, for instance), the irreversible Δ*T*_ad,q-d_ is calculated as Δ*T*_ad,q-d_ = *T*(*S*, *P*_0_)_heating_ − *T*(*S*, *P*)_heating_ (Supplementary Fig. 33). For compression-induced heating, the irreversible Δ*T*_ad,q-d_ is similarly calculated as Δ*T*_ad,q-d_ = *T*(*S*, *P*)_cooling_ − *T*(*S*, *P*_0_)_cooling_. For reversible adiabatic temperature changes upon cyclic pressure changes to above *P*_rev,ad_ (the pressure that must be exceeded in order to fully overcome the hysteresis loop), the reversible temperature change is evaluated by calculating the difference between the temperature curves for cooling at applied pressure and heating at ambient pressure, with Δ*T*_ad,rev_ = |*T*(*S*, *P*)_cooling_ − *T*(*S*, *P*_0_)_heating_| (see Supplementary Note 4 for details). We note that direct measurements under adiabatic conditions—although still challenging for barocaloric materials—will be important for the more accurate and reliable evaluation of adiabatic temperature changes.

### Quasi-isothermal HP-DSC

During quasi-isothermal pressure cycling experiments, heat flow signals were measured as a function of time while applying or removing a hydrostatic pressure of 150 bar and 105 bar for (DA)_2_MnCl_4_ and (NA)_2_CuBr_4_, respectively. The temperature was held at 311.1 K for (DA)_2_MnCl_4_ and 305.5 K for (NA)_2_CuBr_4_, respectively. The pressure was increased linearly at a rate of 6 bar min^–1^ and decreased asymptotically at an average rate of 13 bar min^–1^. To distinguish heat flow signals associated with the pressure-induced phase transitions of each compound from those associated with compression and decompression of He gas, 5 mg of (C_12_H_25_NH_3_)_2_MnCl_4_, which was prepared according to a previously reported procedure^49^, was used as a control sample because it does not undergo any phase transitions until above 330 K, well above the transition temperatures for (DA)_2_MnCl_4_ and (NA)_2_CuBr_4_. The heat flow signals measured during pressure cycling (C_12_H_25_NH_3_)_2_MnCl_4_ at 311 K and 306 K were modeled as a baseline and subtracted from the raw sample data for (DA)_2_MnCl_4_ and (NA)_2_CuBr_4_, respectively, to yield pressure-induced heat flow signals as a function of time (Supplementary Figs. 18d, 19d). Similar to the determination of the transition temperature, *T*_tr_, the extrapolated onset pressures were defined as the transition pressure for pressure-induced endotherms and exotherms. Specifically, the onset pressure was determined by first generating baselines using mutual tangent slopes before and after the pressure-induced transition peak, with the intersection between the baseline and the tangent of the peak representing the onset point. The corresponding pressure at the onset time is reported as the phase transition pressure, and the pressure hysteresis is calculated as the difference between the phase transition pressures during compression and decompression.

During pressure cycling, quasi-isothermal conditions were maintained by adjusting the set temperature of the circulating water bath surrounding the autoclave to compensate for small temperature fluctuations induced by gas compression and decompression. Note that the external thermal control measures were identical for all three samples (control, (DA)_2_MnCl_4_, and (NA)_2_CuBr_4_). Since the rate at which the pressure was decreased was faster than the rate it was increased, decompression resulted in a larger change in temperature (1 K) than compression (<0.3 K) (Supplementary Figs. 18c, 19c). However, these small temperature changes were quickly recovered, such that the measured sample temperatures near the phase transition onsets were in close proximity during both compression and decompression (<0.2 K) (Supplementary Figs. 18e, 19e).

### Heat capacity measurements

A Discovery 2500 DSC with a RCS 90 cooling system (TA Instruments) was used to measure the heat capacity of (DA)_2_MnCl_4_ and (NA)_2_CuBr_4_ using the ASTM Standard E1269. All runs used to calculate heat capacity were performed under the standard manufacturer calibrations. This includes the Tzero calibration, which achieves flat (<5 µW variation) baselines by calibrating the cell to a heat flow model containing thermal resistance and capacitance terms for the sample and reference sides of the cell. In addition, an indium standard was used to calibrate the temperature and cell constant.

The ASTM E1269 method calculates heat capacity from three separate DSC runs, which are performed sequentially. All three runs were comprised of a temperature ramp (10 K min^–1^) over the temperature range of interest with 10 min isothermal holds at the minimum and maximum temperatures to stabilize heat flows before and after the temperature ramps. The first run was conducted with empty pans in the sample and reference position of the DSC to establish a baseline in the absence of sample. Though the DSC baseline is calibrated using the Tzero method, that calibration returns a flat baseline of zero heat flow to an empty cell, while the ASTM baseline accounts for asymmetries that may be introduced due to the introduction of pans to the sample and reference position. The second run was conducted with an empty reference pan and a sample pan containing a sapphire disc to calibrate the heat capacity, and the third run was conducted by replacing the sample pan containing the sapphire with a sample pan containing either (DA)_2_MnCl_4_ or (NA)_2_CuBr_4_.

All samples of (DA)_2_MnCl_4_ and (NA)_2_CuBr_4_ for heat capacity measurements were prepared under an N_2_ atmosphere in Tzero aluminum pans and sealed using a Tzero aluminum press. To ensure adequate heat flows and heat capacity signal, 10 mg or more of sample were used for each heat capacity measurement. In addition, the samples were packed into the pan using the Tzero powder preparation kit to improve thermal contact. The ASTM method specifies that the masses of the empty sample and reference pans should be matched, with ASTM recommending ±0.01 mg variation and TA instruments recommending ±0.05 mg variation. The DSC 2500, however, accounts for pan mass differences and the resulting heat flows due to the heat capacity of aluminum using the T4P heat flow. Therefore, when using the T4P heat flow, the pan mass matching criterion is not as stringent. Nevertheless, pan masses were selected here to be within ±0.1 mg, with the total empty pan mass being near 50 mg.

Non-hermetic Tzero pans were used to allow for He gas to be present over the sample to be consistent with high-pressure DSC measurements and because the non-hermetic lids press directly on the sample, improving thermal contact. The DSC 2500 also features a robotic autosampler, which precisely and repeatably loads the sample and reference pans into the DSC cell. For all three runs, the reference pan remained in the cell and was not moved, with only the sample pan being exchanged by the autosampler. The cell purge gas was He at a flow rate of 50 mL min^–1^. Samples were held under He for at least 30 min prior to the temperature ramp.

To examine the variability in our measurement and obtain a representative average heat capacity curve, the measurements of (DA)_2_MnCl_4_ and (NA)_2_CuBr_4_ were each conducted 8 times. The same reference pan and sapphire pan were used for all measurements, while a new sample pan with new sample was used for each measurement. All (DA)_2_MnCl_4_ and (NA)_2_CuBr_4_ heat capacity measurements were conducted between at least 230 and 360 K, and the averaged data is shown in Supplementary Fig. 31. After noticing a low-temperature feature in the heat capacity of (NA)_2_CuBr_4_, several additional runs were conducted down to 190 K, and an additional run of (DA)_2_MnCl_4_ was also conducted down to 190 K (Supplementary Fig. 32). The ASTM heat capacity was calculated using a feature in the TRIOS software (TA Instruments), which calculates the heat capacity from a set of three ASTM runs (the baseline, sapphire, and sample runs) with each heat capacity calculation representing a unique set of three runs. The heat capacity data was interpolated onto a common temperature axis and averaged at each temperature point to arrive at an average heat capacity curve for (DA)_2_MnCl_4_ and (NA)_2_CuBr_4_. The error was also calculated at each temperature point and is based on the ASTM recommendation, which specifies reporting an error of 2.8 times the standard deviation.

### Powder X-ray diffraction (PXRD)

PXRD data for (DA)_2_MnCl_4_ and (NA)_2_CuBr_4_ were collected on beamline 17-BM-B at the Advanced Photon Source (APS) at Argonne National Laboratory, with an X-ray wavelength of 0.45237 Å. For variable temperature and pressure experiments, approximately 10 mg of sample was loaded into a sapphire capillary (1.52 mm × 1.07 mm × 50.8 mm, Saint-Gobain Crystals). Each capillary was attached to a custom-designed flow cell equipped with a gas valve, which was mounted onto the goniometer head. A syringe pump (Teledyne ISCO D65) was then connected via a 1/16″ gas line to the flow cell and used to control the hydrostatic pressure of He gas (ultra-high purity, 99.999%) from 80 to 360 bar. The internal sample temperature was monitored during PXRD experiments via a K-type thermocouple (0.1 K accuracy) that was in contact with the powder sample within the capillary. The sample temperature was controlled by an Oxford Cryostream (Oxford Cryostream 800+).

In a typical isobaric, variable-temperature PXRD experiment, the sapphire capillary was first equilibrated at 370 K for 10–20 min and then the Cryostream temperature was decreased from 370 to 250 K at a rate of 6 K min^−1^, which led to a change in sample temperature from 335 to 275 K at an average rate of ~3 K min^−1^. During cooling, powder patterns were collected every 10–20 seconds at a temperature interval of ~1 K. Isothermal, variable-pressure experiments were carried out to determine the compressibility of the LT and HT phases of each sample. Prior to each measurement, samples were equilibrated at the set temperature for 20 minutes. Powder patterns were then collected every 5 bar from 80 to 300 bar, equilibrating for 2 min at each pressure.

Diffraction patterns were analyzed using the software TOPAS-Academic^50^. Unit cell parameters of diffraction patterns were determined by using a standard peak search followed by indexing with a single value decomposition approach^51^. A structureless Le Bail refinement was then performed to refine the unit cell parameters. Variable-temperature PXRD data obtained under isobaric conditions are shown in Supplementary Figs. 23–25 for (DA)_2_MnCl_4_ and Supplementary Figs. 26–29 for (NA)_2_CuBr_4_. Comparisons with simulated powder patterns are provided in Supplementary Fig. 30. For both compounds, the temperature and pressure dependence of interlayer distances, unit cell parameters, and specific volumes are provided in Supplementary Figs. 1–5. The thermal expansion coefficients, isothermal compressibility, and volume change during transition each compound are tabulated in Supplementary Tables 9 and 12, and unit cell parameters are tabulated in Supplementary Tables 14 and 15.

### Determination of *T*_tr_ from PXRD

Isobaric, variable-temperature experiments were carried out from 80 to 360 bar to determine the phase transition temperature, *T*_tr_, for (DA)_2_MnCl_4_ and (NA)_2_CuBr_4_ (Supplementary Figs. 23–29). In particular, we evaluated the pressure dependence of the exothermic transition from the high-temperature (HT) to low-temperature (LT) phases during cooling, *T*_tr,cooling_, because temperature control during cooling was more reliable than during heating. To identify *T*_tr_, changes in the high-intensity (00*l*) peaks in the low-angle region (<4°) of the powder patterns were monitored, with a particular focus on the emergence of a higher-angle shoulder on the right side of each peak that indicates the emergence of the smaller unit cell of the LT. Note that the shoulder intensity as small as 2% of the parental peak intensity was discernible. Since the sample temperature was varied continuously—similar to DSC experiments—the onset temperature for the emergence of the LT phase is reported as *T*_tr_. More specifically, the transition onset temperature was estimated to be just above the temperature at which the first powder pattern with the shoulder peaks was. This approach enabled the identification of transition temperatures with an estimated accuracy of 1–2 K.

To further improve the accuracy of *T*_tr_ determination, we used data from the thermocouple embedded in the sample capillary that recorded the sample temperature every 2 s. During cooling, the thermocouple temperature trace features a peak, which is attributed to the latent heat of the exothermic transition. Despite the seemingly small signal, this feature can be clearly detected by monitoring the change in sample cooling rate over time. Specifically, the phase transition leads to a sudden spike in the first derivative of sample temperature with respect to time. The onset of the phase transition was assigned to the temperature at which the first local maximum of the cooling rate curve appears, as this feature—which corresponds to the tangent of the onset transition peak—can be reliably identified across all datasets. Additionally, the uncertainty associated with the determination of the transition onset temperature was defined as the full width at half maximum of the cooling rate peak, which ranges from 1 to 2 K. Note that the *T*_tr_ values were determined using the sample thermocouple temperature data. At ambient pressure, the onset transition temperatures, *T*_tr,cooling_, determined by these PXRD experiments were in excellent agreement with those determined by DSC, with a difference of less than 0.4 K. Since the goal of these experiments was to determine the pressure dependence of *T*_tr_, rather than the absolute values of *T*_tr_, the transition temperatures determined via PXRD were calibrated using the *T*_tr,cooling_ value determined by DSC at ambient pressure for consistency.

### Determination of Δ*V*_tr_ from PXRD

From the temperature dependence of unit cell volumes at ambient pressure, the specific volume change during the order–disorder transition, Δ*V*_tr_, was calculated as the difference between the LT and HT phase volume at the transition temperature (*T*_tr_), with Δ*V*_tr_ = *V*_HT,tr_ – *V*_LT,tr_ (Supplementary Fig. 4). The volume at *T*_tr_ for the LT and HT phases was extrapolated from unit cells determined below and above *T*_tr_, respectively, using thermal expansion coefficients for each phase determined at ambient pressure. Specific volume changes from 200 to 300 bar were determined using unit cell volumes measured as a function of pressure at constant temperature (273 K for both LT phases, 323 K for the HT phase of (DA)_2_MnCl_4_, and 314 K for the HT phase of (NA)_2_CuBr_4_). Thermal expansion coefficients were assumed to be independent of pressure over this pressure range, and the thermal expansion coefficients determined for each phase at 1 bar were used to extrapolate the isothermal specific volumes to *T*_tr_ at each pressure.

### Helium pycnometry

Skeletal densities of the samples were determined using an InstruQuest μ-ThermoPyc He pycnometer. In a typical measurement, ~150 mg of sample was transferred to the sample holder, and the sample mass was obtained. The holder was then placed in the instrument test chamber, and the headspace was evacuated and refilled five times with He. The sample was then cycled multiple times through the phase transition, with the chamber volume determined every 2–5 °C away from the transition and every 0.5–1.0 °C close to the transition. For a measurement of unknown volume in a sample holder, *V*_test_, in the sample chamber with a known volume of *V*_sc_, at a temperature *T*_sc_, the sample chamber was first pressurized to ~200 kPa and the initial pressure (*P*_*i*_) was measured. A reference chamber of known volume (*V*_rc_) was initially equilibrated with ambient pressure (*P*_a_) and temperature (*T*_rc_). The sample chamber was then connected to a reference chamber. After equilibration, the final pressure in the combined chamber, *P*_f_, was measured. Since the system is closed when the two chambers are connected, the ideal gas law and conservation of moles can be used to provide the following relationship:

8 $$\frac{{P}_{i}({V}_{{{{{{\rm{sc}}}}}}}-{V}_{{{{{{\rm{test}}}}}}})}{R{T}_{{{{{{\rm{sc}}}}}}}}\,+\,\frac{{P}_{a}{V}_{{{{{{\rm{rc}}}}}}}}{R{T}_{{{{{{\rm{rc}}}}}}}}\,=\,\frac{{P}_{f}({V}_{{{{{{\rm{sc}}}}}}}-{V}_{{{{{{\rm{test}}}}}}})}{R{T}_{{{{{{\rm{sc}}}}}}}}\,+\,\frac{{P}_{f}{V}_{{{{{{\rm{rc}}}}}}}}{R{T}_{{{{{{\rm{rc}}}}}}}}$$

where the left- and right-hand sides denote the moles of gas before and after connecting the sample and reference chamber, respectively.

Solving for *V*_test_ gives:

9 $${V}_{{{{{{\rm{test}}}}}}}\,=\,{V}_{{{{{{\rm{sc}}}}}}}-{V}_{r}\left(\frac{{T}_{s}}{{T}_{r}}\right)\frac{({P}_{f}-{P}_{a})}{({P}_{i}-{P}_{f})}$$

Since *V*_test_ includes the volume of the empty sample holder (*V*_h_) that had been measured previously, sample volume (*V*_sample_) was determined by subtracting *V*_h_ from *V*_test_, with *V*_sample_ = *V*_test_ − *V*_h_. For each volume measurement, the temperature was fully equilibrated until the temperature variation was less than 0.2 °C. At each temperature, the chamber volumes were measured five times to obtain good statistics. Prior to measurement of the sample, a calibration run of the empty sample holder was performed over the same temperature range. The sample mass was redetermined after the measurement and found to have decreased by no more than 0.5 mg. Uncertainties of the reported densities were determined by propagation of the standard deviations of the empty and filled chamber volumes and the sample mass. Δ*V*_tr_ values for both compounds are summarized in Supplementary Table 10.

### X-ray crystallography

X-ray diffraction analyses were performed on single crystals coated with Paratone-N oil and mounted on MiTeGen microloops using Dow Corning high vacuum grease. The temperature during data collection was controlled from 100 to 335 K using an Oxford Cryostreams nitrogen flow apparatus. Crystals were first mounted at 270 K, and 270 K datasets were collected. Crystals were then cooled to 100 K for 100 K data collection. After 100 K datasets, high-temperature datasets were collected: 330 K for (DA)_2_MnCl_4_ and 335 K for (NA)_2_CuBr_4_. The temperature was manipulated at a rate of 60 K h^**–**1^. The intensities of the reflections were collected by a Bruker D8 diffractometer with CMOS area detector (MoKα radiation, *λ* = 0.71073 Å). The collection method involved 0.5° scans in *ω* at 23° in 2*θ* with a detector distance of 9 cm for (DA)_2_MnCl_4_ and 8 cm for (NA)_2_CuBr_4_. Data integration down to 0.84 Å resolution was carried out using SAINT V8.37A with reflection spot size optimization^52^. Absorption corrections were made with the program SADABS^52^. All single-crystal structures were solved by the Intrinsic Phasing methods and refined by least-squares methods against *F*^2^ using SHELXT-2014^53^ and SHELXL-2018^54^ with the OLEX2 interface^55^. All non-H atoms, including all the disorder atoms, were located in difference-Fourier maps and then refined anisotropically. Hydrogen atoms were placed at idealized positions and refined using a riding model. The isotropic displacement parameters of all hydrogen atoms were constrained to be 1.2 times the parameters of the atoms they were linked to (1.5 times for methyl groups). Space groups were determined based on the systematic absences and the statistical indicators, such as the *E*-value and the CFOM values. The absence of higher symmetry was further confirmed by Platon/Addsym^56^. Unit cell information from ambient-pressure PXRD experiments was used to help select an appropriate data collection strategy. Atomic displacement parameter plots of alkylammonium cations are provided in and Fig. 8 and Supplementary Fig. 36, and hydrogen bonding geometry of (DA)_2_MnCl_4_ and (NA)_2_CuBr_4_ are shown in Supplementary Figs. 34 and 35, respectively. Selected geometric parameters are tabulated in Supplementary Tables 16–20, and details of data collection and refinement are summarized in Supplementary Tables 21 and 22. We acknowledge that there are limitations with using X-ray single crystal diffraction to determine the geometry of small organic cations in a heavy element inorganic framework, especially at high temperatures and when substantial disorder is expected to be present.

### Thermal conductivity measurements

The thermal conductivity of each sample was measured by the flash diffusivity method (ASTM E1461-13) using a Netzsch LFA 467 HyperFlash instrument (Supplementary Fig. 42). The samples were prepared in pellets of approximately 12.9 mm in diameter and 1 mm thickness. The measurements were carried out under a Helium atmosphere at 10 °C below and 15 °C above the transition temperature for the LT and HT phases, respectively, of (DA)_2_MnCl_4_, and at 10 °C below and above the transition temperature for the LT and HT phases, respectively, of (NA)_2_CuBr_4_. The measurement results were not corrected for thermal expansion due to phase changes.

## Supplementary information

Supplementary Information

## Data Availability

The main data supporting the findings of this study are available within the paper and its Supplementary Information. Other data are available from the corresponding author upon request. Crystallographic data have been made available free of charge from the Cambridge Crystallographic Data Centre under reference numbers CCDC 2075108–2075113.
