# Supplementary material for: Colossal barocaloric effects with ultralow hysteresis in two-dimensional metal–halide perovskites
Source: Nat Commun. 2022 May 9;13:2536. doi: 10.1038/s41467-022-29800-9 (PMC9085852; doi:10.1038/s41467-022-29800-9)
Supplement: Supplementary file 1 — Supplementary Information [file 41467_2022_29800_MOESM1_ESM.pdf]

Supplementary Information

Colossal Barocaloric Effects with Ultralow Hysteresis in Two-Dimensional Metal–Halide Perovskites

Jinyoung Seo, Ryan D. McGillicuddy, Adam H. Slavney, Selena Zhang, Rahil Ukani, Andrey A. Yakovenko, Shao-Liang Zheng, Jarad A. Mason*

*To whom correspondence should be addressed to: mason@chemistry.harvard.edu

This PDF file includes:

Supplementary Notes
Supplementary Figures 1 to 44
Tables 1 to 22
Supplementary References

Table of Contents

**Supplementary Note 1**	Entropy contribution of conformational disorder in two-dimensional perovskites	**S4**
**Supplementary Note 2**	Crystallographic characterization of disorder in alkylammonium chains	**S5**
**Supplementary Note 3**	Volumetric entropy changes (ΔS_v)	**S5**
**Supplementary Note 4**	Evaluation of reversible barocaloric effects	**S6**
**Supplementary Note 5**	Impacts of extrinsic factors on low-pressure reversibility	**S7**
**Supplementary Note 6**	Variable-temperature infrared spectroscopy	**S9**
**Supplementary Note 7**	Environmental impact of cooling	**S11**
**Supplementary Fig. 1**	Interlayer distances obtained from variable-temperature powder X-ray diffraction	**S12**
**Supplementary Fig. 2**	Unit cell parameters for $(DA)_2MnCl_4$ obtained from variable-temperature PXRD	**S13**
**Supplementary Fig. 3**	Unit cell parameters for $(NA)_2CuBr_4$ obtained from variable-temperature PXRD	**S14**
**Supplementary Fig. 4**	Specific volumes obtained from variable-temperature PXRD	**S15**
**Supplementary Fig. 5**	Pressure dependence of specific volumes obtained from variable-pressure PXRD	**S16**
**Supplementary Fig. 6**	Pressure dependence of transition temperatures (dT/dP) for $(DA)_2MnCl_4$	**S17**
**Supplementary Fig. 7**	Pressure dependence of transition temperatures (dT/dP) for $(NA)_2CuBr_4$	**S18**
**Supplementary Fig. 8**	Phase diagrams for $(DA)_2MnCl_4$ and $(NA)_2CuBr_4$	**S19**
**Supplementary Fig. 9**	Illustration of powder and single-crystal samples	**S20**
**Supplementary Fig. 10**	Isobaric and isothermal entropy curves [single crystal, $(DA)_2MnCl_4$, 1–150 bar]	**S21**
**Supplementary Fig. 11**	Isobaric and isothermal entropy curves [single crystal, $(NA)_2CuBr_4$, 1–150 bar]	**S22**
**Supplementary Fig. 12**	Differential scanning calorimetry traces for $(DA)_2MnCl_4$ (powder, 1–150 bar)	**S23**
**Supplementary Fig. 13**	Differential scanning calorimetry traces for $(NA)_2CuBr_4$ (powder, 1–150 bar)	**S24**
**Supplementary Fig. 14**	Isobaric and isothermal entropy curves [powder, $(DA)_2MnCl_4$, 1–150 bar]	**S25**
**Supplementary Fig. 15**	Isobaric and isothermal entropy curves [powder, $(NA)_2CuBr_4$, 1–150 bar]	**S26**
**Supplementary Fig. 16**	Reversible isothermal entropy changes for powder samples (1–150 bar)	**S27**
**Supplementary Fig. 17**	Reversible barocaloric effects at low pressures for single-crystal samples	**S28**
**Supplementary Fig. 18**	Evaluation of pressure hysteresis via quasi-isothermal HP-DSC for $(DA)_2MnCl_4$	**S29**
**Supplementary Fig. 19**	Evaluation of pressure hysteresis via quasi-isothermal HP-DSC for $(NA)_2CuBr_4$	**S30**
**Supplementary Fig. 20**	Isobaric and isothermal entropy curves [powder, $(DA)_2MnCl_4$, 300–500 bar]	**S31**
**Supplementary Fig. 21**	Isobaric and isothermal entropy curves [powder, $(NA)_2CuBr_4$, 300–500 bar]	**S32**
**Supplementary Fig. 22**	Reversible barocaloric effects at 300–500 bar for powder samples	**S33**
**Supplementary Fig. 23**	Variable-temperature PXRD for $(DA)_2MnCl_4$ at 1 bar	**S34**
**Supplementary Fig. 24**	Variable-temperature PXRD for $(DA)_2MnCl_4$ at 300 bar	**S35**
**Supplementary Fig. 25**	Variable-temperature PXRD for $(DA)_2MnCl_4$ at 360 bar	**S36**
**Supplementary Fig. 26**	Variable-temperature PXRD for $(NA)_2CuBr_4$ at 1 bar	**S37**
**Supplementary Fig. 27**	Variable-temperature PXRD for $(NA)_2CuBr_4$ at 120 bar	**S38**
**Supplementary Fig. 28**	Variable-temperature PXRD for $(NA)_2CuBr_4$ at 200 bar	**S39**
**Supplementary Fig. 29**	Variable-temperature PXRD for $(NA)_2CuBr_4$ at 300 bar	**S40**

**Supplementary Fig. 30**	Comparison of experimental PXRD patterns to calculated diffraction patterns	**S41**
**Supplementary Fig. 31**	Temperature dependence of specific heat capacity (230–360 K)	**S42**
**Supplementary Fig. 32**	Temperature dependence of specific heat capacity (190–360 K)	**S43**
**Supplementary Fig. 33**	Calculations of adiabatic temperature changes via the quasi-direct method	**S44**
**Supplementary Fig. 34**	Hydrogen bonding interactions of DA chains within the Mn–Cl pocket	**S45**
**Supplementary Fig. 35**	Hydrogen bonding interactions of NA chains within the Cu–Br pocket	**S46**
**Supplementary Fig. 36**	Conformations of alkylammonium chains at 100 K	**S47**
**Supplementary Fig. 37**	Variable-temperature infrared spectra	**S48**
**Supplementary Fig. 38**	DSC traces at ambient pressure for $(NA)_2CuBr_4$ (190–360 K)	**S49**
**Supplementary Fig. 39**	Thermogravimetric analysis	**S50**
**Supplementary Fig. 40**	Thermal stability	**S51**
**Supplementary Fig. 41**	Chemical stability	**S52**
**Supplementary Fig. 42**	Thermal conductivity	**S53**
**Supplementary Fig. 43**	Variable-temperature atomic force microscope imaging during thermal cycling	**S54**
**Supplementary Fig. 44**	Isobaric HP-DSC measurements for single crystals directly grown on a substrate	**S55**

**Supplementary Table 1**	Phase-change thermodynamics of $(C_nH_{2n+1}NH_3)_2MnCl_4$	**S56**
**Supplementary Table 2**	Phase-change thermodynamics of $(C_nH_{2n+1}NH_3)_2CuX_4$ (X = Cl, Br)	**S57**
**Supplementary Table 3**	Phase-change thermodynamics of $(C_nH_{2n+1}NH_3)_2CdCl_4$ and $(C_nH_{2n+1}NH_3)_2PbI_4$	**S58**
**Supplementary Table 4**	Prediction of dT/dP values for $(C_nH_{2n+1}NH_3)_2MCl_4$ (M = Mn, Cu)	**S59**
**Supplementary Table 5**	Barocaloric effects of representative barocaloric materials	**S60**
**Supplementary Table 6**	Predicted thermodynamic efficiencies for selected barocaloric materials	**S61**
**Supplementary Table 7**	HP-DSC experiments for single-crystal and powder samples (1–150 bar)	**S62**
**Supplementary Table 8**	dT/dP values measured from HP-DSC and *in situ* PXRD experiments	**S63**
**Supplementary Table 9**	Isothermal compressibility, thermal expansion coefficient, ΔS_+, and ΔS_V	**S64**
**Supplementary Table 10**	Comparison of predicted and experimentally determined dT/dP values	**S65**
**Supplementary Table 11**	Thermodynamic data from quasi-isothermal HP-DSC experiments	**S66**
**Supplementary Table 12**	Volume changes determined by isobaric PXRD experiments	**S67**
**Supplementary Table 13**	Summary of characteristic infrared bands	**S68**
**Supplementary Table 14**	Unit cell parameters for $(DA)_2MnCl_4$ from Le Bail refinements	**S69**
**Supplementary Table 15**	Unit cell parameters for $(NA)_2CuBr_4$ from Le Bail refinements	**S70**
**Supplementary Table 16**	Selected geometric parameters from single crystal structures of $(DA)_2MnCl_4$	**S71**
**Supplementary Table 17**	Selected geometric parameters from single crystal structures of $(NA)_2CuBr_4$	**S72**
**Supplementary Table 18**	Dihedral angles of alkylammonium chains for $(DA)_2MnCl_4$	**S73**
**Supplementary Table 19**	Dihedral angles of alkylammonium chains for $(NA)_2CuBr_4$	**S74**
**Supplementary Table 20**	Donor–acceptor (N···X) distances and bond angles	**S75**
**Supplementary Table 21**	Crystallographic data for $(DA)_2MnCl_4$ collected at 100 K, 270 K, and 330 K	**S76**
**Supplementary Table 22**	Crystallographic data for $(NA)_2CuBr_4$ collected at 100 K, 270 K, and 335 K	**S77**

References **S78**

Supplementary Note 1. Estimating the entropy contribution of conformational disorder
For an alkylammonium chain $C_nH_{2n+1}NH_3^+$, there are n–2 rotatable C–C bonds that can contribute to the formation of different conformers. The conformation of alkylammonium chains can be described through a sequence of dihedral angles—often referred to as Hoffmann's notation—that represent energy minima with the terms g^+, g^-, and t denoting dihedral angles of +60 (gauche), −60 (gauche), and 180° (trans), respectively. The change in solid-state configurational entropy attributed to different rotameric states can be described as $\Delta S_{\mathrm{configuration}} = \mathrm{R} \ln W$, where W is the ratio between the number of configurations in the disordered and ordered chains ($W = N_{\mathrm{disorder}} / N_{\mathrm{order}}$). Thus, the maximum entropy change associated with the conformational disordering of an alkylammonium chain from an all-trans configuration can be estimated as $\mathrm{R} \ln 3^{n-2}$.

Depending on how neighboring chains influence one another in each phase, the average number of thermally accessible rotameric states for each C–C bond—defined here as the chain flexibility number, ϕ–can deviate from 3 (ref. 1). Note that, the flexibility number depends on the energetic difference between each conformer, with a flexibility number of 2.85 leading to reliable predictions for the melting thermodynamics of linear organic molecules[2]. In two-dimensional perovskites, the number of C–C bonds associated with the disordering processes can be reduced by steric restrictions (imposed by both the halide pockets and neighboring alkylammonium chains), intrachain correlations of C–C bond rotations[1] (such as the preferential formation of {gtg'} kinks[3,4]), and residual degrees of freedom present in the low-temperature phase. The number of accessible conformations in the disordered phase can then be approximated as $(\phi)^{n-2-\beta}$, where β corresponds to a restriction parameter that reflects a decrease in the conformational degrees of freedom that become accessible during the order-disorder transition. In addition to C–C bond rotation, the entire alkylammonium chain can reorient (or "flip") between two energetically equivalent orientations within the metal–halide pocket. This chain flipping can occur as an isolated minor transition or be coupled to the major order–disorder transition[3–5], and the entropy change associated with chain flipping can be accounted for by adding the term R ln 2.

Based on this, the conformational entropy changes for $(DA)_2MnCl_4$ and $(NA)_2CuBr_4$ can be estimated using

$$\Delta S_{\mathrm{tr}} = \Delta S_{\mathrm{flipping}} + \Delta S_{\mathrm{conformational}} = 2\mathrm{R} \ln 2 + 2\mathrm{R} \ln (\phi)^{n-2-\beta} \quad (1)$$

If we assume $\phi = 2.85$, then the full entropy of each transition, ΔS_{tr}, could be accounted for with a β of 1.88 for $(DA)_2MnCl_4$ and 4.73 for $(NA)_2CuBr_4$. Interestingly, for $(DA)_2MnCl_4$, the estimated value for β suggests that roughly six C–C bonds are rotatable ($10–2–\beta = 6.1$), which is corroborated by the 330 K crystal structure, where each alkylammonium chain is disordered over a special position and has a conformation with six C–C dihedral angles of 150–166° that deviate from the ideal trans dihedral angle of 180 (Supplementary Table 18). The lower transition entropies of $(NA)_2CuBr_4$ measured via HP-DSC could be explained by a smaller increase in the number of newly rotating C–C bonds ($9–2–\beta = 2.3$) that leads to a smaller change in solid-state conformational entropy during the order–disorder transition. Differences in chain flexibility and vibrational entropy may also contribute to the lower entropy change of $(NA)_2CuBr_4$. Here, the entropy contributions are estimated using the Boltzmann formula, with assumptions that the entropy changes originate mostly from order-disorder transitions. We note that, however, the exact origin of the entropy changes—as well as the dynamics and local structures of the chains—need further investigations.

Supplementary Note 2. Crystallographic characterization of disorder in alkylammonium chains

To obtain additional insight into any differences in conformational disorder between $(DA)_2MnCl_4$ and $(NA)_2CuBr_4$, we analyzed atomic displacement parameters obtained from the crystal structure refinement process, as these parameters report how much each atom deviates from its equilibrium position. Thermal displacement parameters contain information about residual motion (such as rotations and vibration) and static, configurational disorder. In Fig. 8, equivalent isotropic displacement parameters (U_{equiv}) of all N and C atoms are shown for the LT and HT phases, as well as for 100 K structures.

In $(DA)_2MnCl_4$, the transition from the LT to HT phase results in a large increase in the U_{equiv} values of all DA atoms, and the magnitude of the increase in U_{equiv} increases along the chain from the N atoms of the ammonium headgroup to the C atom of the terminal methyl group (Fig. 8c). This is reflective of the large increase in dynamic disorder that accompanies the order-disorder transition, with increasing disorder created further away from the ammonium headgroup that is pinned to the inorganic layer. In the LT structure of $(NA)_2CuBr_4$, NA chains adopt two conformations—alternating between chains with a gauche C1–C2 bond (chain A) and those with a gauche C2–C3 bond (chain B), each of which is modeled with two-part disorder (Fig. 8b). In addition, the NA chains are distorted from ideal trans configurations near the methyl ends with C7–C8 dihedral angles of +159°/–170° in chain A (Part 1/Part 2) and +164°/–164° in chain B (Part 1/Part 2), as well as the additional distortion in the C3–C4 bond (–159°/+169°) in chain A (Supplementary Table 19). Moreover, the NA chain atoms display U_{equiv} values that are nearly double those in the DA chain in the LT phase, while the U_{equiv} values in the HT phase are similar in both compounds (Fig. 8d). As a result, the difference in U_{equiv} values between the LT and HT phase is smaller for $(NA)_2CuBr_4$ than for $(DA)_2MnCl_4$, which is consistent with the smaller change in entropy for $(NA)_2CuBr_4$ during the order-disorder transition.

At 100 K, alkylammonium chains in both $(DA)_2MnCl_4$ and $(NA)_2CuBr_4$ are fully ordered with similar U_{equiv} values and all trans C–C bonds except for a single gauche C–C bond (Figs. 8c,d, Supplementary Fig. 36). This indicates that the disorder in the LT phase of $(NA)_2CuBr_4$ is introduced at a temperature between 100 K and 270 K, where the LT phase crystal structure was collected. Although no heat flow signals indicative of a minor phase transition are observed down to at least 190 K by DSC (Supplementary Fig. 38), a broad feature is observed from 220–250 K in the heat capacity of $(NA)_2CuBr_4$ that is consistent with the gradual activation of new conformational degrees of freedom for the NA chains (Supplementary Fig. 32).

Supplementary Note 3. Volumetric entropy changes (ΔS_V)

For order-disorder phase transitions of long-chain hydrocarbon molecules (such as melting transitions of *n*-alkanes and polyethylene), it has been discussed that the transition entropy, ΔS_{tr}, can be decomposed into a conformational entropy term (ΔS_c) and volumetric entropy term (ΔS_V)—at the level of phenomenological thermodynamics—with $\Delta S_{tr} = \Delta S_c + \Delta S_V$ (refs. 6,7). Here, ΔS_V is a purely phenomenological term that refers to the entropy increase associated with a volume expansion without any disordering processes (for heating-induced disordering transitions). Using the Maxwell relation $(\partial S/\partial V)_P = (\partial P/\partial T)_V$, ΔS_V can be approximated by $\Delta S_V = (\alpha/\kappa)\cdot\Delta V_{tr}$, where α, κ, and ΔV_{tr} denote isobaric thermal expansivity, isothermal compressibility, and the specific volume change of the transition at ambient pressure, respectively. Because the exact value of ΔS_V

depends on a specific thermodynamic path taken, the validity of this calculation has been challenged[8]; however, this approach can be applied to a variety of phase transitions—including order-disorder transitions in organic plastic crystals—as long as the ratio of α/κ does not change significantly across the phase transition[9].

To obtain additional insight into the entropy changes during order-disorder phase transitions of 2-D perovskites, we calculated the relative contribution of ΔS_V to the total entropy changes using isothermal compressibility and thermal expansion coefficients obtained from variable-temperature and variable-pressure PXRD experiments. Since the phase transition leads to a somewhat substantial change in the ratio of α/κ (with a 17% and 40% decrease from the LT to HT phases for $(DA)_2MnCl_4$ and $(NA)_2CuBr_4$, respectively), we have calculated ΔS_V through two methods: 1) using α and κ values from the HT phase only, and 2) using α and κ values averaged across the phase transition (Supplementary Table 9). The calculations show that the contribution of ΔS_v to the transition entropy is relatively large (40–50%). As might be expected, the magnitude of the relative contribution is similar to that in melting transitions of *n*-alkanes[6]. Overall, these calculations highlight that the volume change makes a substantial contribution to the total entropy change associated with the order-disorder transition.

Supplementary Note 4. Evaluation of reversible barocaloric effects

In addition to P_{rev}, which corresponds to the minimum pressure required to achieve a non-zero, reversible isothermal entropy change, $\Delta S_{it,rev}$ (ref. 10), the minimum pressure required to induce a non-zero reversible adiabatic temperature change, $P_{rev,ad}$, is an important metric in the evaluation barocaloric performance.

For normal barocaloric materials with a positive barocaloric coefficient ($dT/dP > 0$), P_{rev} corresponds to the pressure required to shift the onset temperature of the exothermic transition ($T^{exo}_{tr,onset}$) to match the onset of the endothermic transition ($T^{endo}_{tr,onset}$) at 1 bar, which can be determined from the barocaloric coefficient associated with the exothermic transition, $(dT/dP)_{cooling}$, and the thermal hysteresis at 1 bar, ΔT_{hys}:

$$P_{rev} = \Delta T_{hys} / (dT/dP)_{cooling} \quad (2)$$

In order to induce a non-zero reversible adiabatic temperature change ($\Delta T_{ad,rev}$), the exothermic peak needs to be shifted further, such that the end temperature of the exothermic transition ($T^{exo}_{tr,\,end}$) matches the end temperature of the endothermic transition ($T^{endo}_{tr,end}$). Note that the end temperature ($T_{tr,end}$) is determined as the intersection between the DSC baseline and the tangent to the region of the DSC peak with the steepest slope after the peak. The difference between $T_{tr,onset}$ and $T_{tr,end}$ corresponds to the transition peak width (ΔT_{width}). From this relationship, $P_{rev,ad}$ can be calculated as

$$P_{rev,ad} = (\Delta T_{hys} + \Delta T^{exo}_{width} + \Delta T^{endo}_{width}) / (dT/dP)_{cooling} \quad (3)$$

where T^{exo}_{width} and T^{endo}_{width} denote the width of the exothermic and endothermic transition peaks, respectively. P_{rev} and $P_{rev,ad}$ for $(DA)_2MnCl_4$ and $(NA)_2CuBr_4$ are summarized in Supplementary Table 7. Note that the full phase transition entropy is also captured at a pressure above $P_{rev,ad}$.

For the evaluation of reversible barocaloric effects, it is important to distinguish between barocaloric effects that are reversible only under isothermal cycling conditions versus those that are reversible under both isothermal and adiabatic cycling conditions.

For an operating pressure above $P_{rev,ad}$, the completion temperature for the ordering transition on a high-pressure cooling entropy curve, $S(T, P)_{cooling}$, is shifted past the completion temperature for the disordering transition on an ambient-pressure heating entropy curve, $S(T, P_0)_{heating}$, defining the region over which reversible barocaloric effects can be obtained under both isothermal and adiabatic cycling conditions (Fig. 6a,b, Supplementary Fig. 17c). When cycling between P and P_0, both isothermal pressure changes (i.e., vertical trajectories on the T–S plot) and adiabatic temperature changes (i.e., horizontal trajectories on the T–S plot) can be defined and evaluated. Specifically, reversible isothermal entropy changes, $\Delta S_{it,rev}$, are quantified by subtracting $S(T, P)_{cooling}$ from $S(T, P_0)_{heating}$ (referred to as the "subtraction method"), and reversible adiabatic temperature changes, $\Delta T_{ad,rev}$, are evaluated by calculating the difference between temperature curves, $T(S, P)$, for cooling at applied pressure and heating at ambient pressure, with $\Delta T_{ad,rev} = |T(S, P)_{cooling} - T(S, P_0)_{heating}|$. The calculated $\Delta T_{ad,rev}$ values are plotted as a function of starting temperature from heating curves at 1 bar and cooling curves at applied pressure for compression-induced temperature increase and decompression-induced temperature decrease, respectively (see Supplementary Fig. 17g, Supplementary Figs. 22e,f).

For an operating pressure between P_{rev} and $P_{rev,ad}$, a barocaloric material can be cycled isothermally to drive reversible isothermal entropy changes through partial phase transitions—that is, a cycle in which only a fraction of the material undergoes the phase transition reversibly[10–12]. The reversible isothermal entropy changes associated with partial phase transitions, which could be used to drive a Stirling-like cooling cycle[13], can be quantified by calculating the overlap between compression-induced and decompression-induced isothermal entropy curves (referred to as the "overlap method")[10]. Since the subtraction method deals with total entropy values and does not allow for the possibility of cycling through a partial phase transition or minor hysteresis loop, the subtraction method is not suitable for evaluating reversible isothermal entropy changes at this pressure range. Thus, for consistency, reversible isothermal entropy changes, as well as RC_{rev}, were quantified using the overlap method for all pressure ranges (Figs. 3 and 5; Supplementary Figs. 16 and 17). For the data obtained above $P_{rev,ad}$, both $\Delta T_{ad,rev}$ and $\Delta S_{it,rev}$ were also evaluated through the subtraction method using the entropy curves that include the contributions from heat capacity (Fig. 6, Supplementary Figs. 17 and 22).

Supplementary Note 5. Impacts of extrinsic factors on low-pressure reversibility

When calculating reversible entropy changes from isobaric high-pressure differential scanning calorimetry (HP-DSC) experiments—especially at the pressure range above P_{rev} and below $P_{rev,ad}$—both the width of the phase transition (ΔT_{width}) and the thermal hysteresis associated with the phase transition (ΔT_{hys}) contribute to the magnitude of the reversible isothermal entropy change that can be achieved for a particular shift in pressure. As also emphasized in Supplementary Note 4, the transition width becomes particularly important for low driving pressures.

Given its thermodynamic origin, the factors that determine the pressure sensitivity of order-disorder transitions in 2-D perovskites are mostly intrinsic to the microscopic structure and composition of a given compound and do not have a strong dependence on extrinsic factors or

nonequilibrium effects. However, phase transition width and hysteresis—which arise from nonequilibrium, kinetic effects—are typically sensitive to extrinsic factors associated with experimental conditions, such as sample size, particle size, and the quality of thermal contact between the sample and DSC pan. As such, optimizing these parameters should provide a way of enhancing reversible barocaloric effects at low pressures.

In isobaric DSC experiments, one of main factors that influences the transition peak width is the thermal gradient across the sample during a phase transition. It is well known that this thermal gradient (often called a thermal lag) can be reduced by using a more thermally conductive purge gas (such as He), reducing the sample size, or improving the quality of thermal contact between the sample and the sample pan that is in direct contact with the DSC sensor[14,15]. In our HP-DSC experiments, we first used microcrystalline powders of 2-D perovskites that were loosely packed in the sample pan. Since the majority of a loosely packed powder sample will not be in direct contact with the sample pan—particularly for layered compounds—heat transfer will be inefficient during DSC measurements, which can lead to relatively large thermal gradients and increase the temperature width of DSC peaks far beyond the amount that is intrinsic to the phase transition of a particular compound.

To investigate this and to minimize the influence of extrinsic factors on transition width, we carried out a series of isobaric HP-DSC experiments on single-crystal samples of $(DA)_2MnCl_4$ and $(NA)_2CuBr_4$ that featured much higher quality thermal contact with the sample pan (Supplementary Fig. 9). This high-quality thermal contact was achieved by pressing down on a large single crystal of each compound within the DSC pan. Importantly, we found that this improved thermal contact leads to a substantial decrease in transition peak width for both compounds (Fig. 4). Moreover, the lower transition peak widths lead to a substantial decrease in $P_{\mathrm{rev,ad}}$. Specifically, $P_{\mathrm{rev,ad}}$ for $(DA)_2MnCl_4$ decreases from 265 bar (powder sample) to 178 bar (single crystal sample) and from 148 bar (powder) to 73 bar (single crystal) for $(NA)_2CuBr_4$. As expected, the quality of thermal contact does not have any impact on the pressure sensitivity, $\mathrm{d}T/\mathrm{d}P$, of the phase transition. These results are summarized in Supplementary Table 7.

We note that the ΔT_{hys} values tend to be slightly larger for single-crystal samples than powder samples, with an increase of 0.3 K and 0.6 K for $(DA)_2MnCl_4$ and $(NA)_2CuBr_4$, respectively (Supplementary Table 7). Though the difference is small, we can tentatively attribute the difference to changes in DSC peak shapes, which are influenced by several extrinsic factors associated with experimental conditions. Since thermal hysteresis is calculated as the difference between the transition onset temperature during heating and the transition onset temperature during cooling—with the onset temperature defined as the intersection between a baseline determined away from the transition and a tangent line at the maximum slope of the DSC peak—the magnitude of the hysteresis is highly sensitive to peak shape. All other things being equal, broader peaks will lead to a lower transition onset temperature during heating and a higher transition onset temperature during and, consequently, a lower thermal hysteresis. As shown in Fig. 4, the single-crystal samples have noticeably sharper DSC peaks that are likely the result of (*i*) improved thermal contact (and reduced thermal lag during the temperature scan), (*ii*) smaller sample sizes, and (*iii*) a narrower distribution of crystallite sizes relative to powder samples (since there may be a small crystal-size dependence to the phase transition temperature). In addition, in the single-crystal samples, the density of lattice defects—which can serve as phase-transition

nucleation sites that contribute to peak broadening—is also likely to be smaller. We believe that these factors all collectively contribute to the slightly increased thermal hysteresis in single-crystal samples. We note, however, that the transition hysteresis width calculated based on the difference in peak temperatures (rather than the conventional thermal hysteresis calculated using onset temperatures) is lower by ~1 K for single crystal samples of both $(DA)_2MnCl_4$ and $(NA)_2CuBr_4$ (Supplementary Table 7).

Beyond the technological implications of realizing large, reversible barocaloric effects at low pressures, our results emphasize the importance of considering extrinsic factors when evaluating the intrinsic potential of new classes of barocaloric materials. We note that understanding contributions of extrinsic factors has been important to evaluating magnetocaloric[16,17], electrocaloric[18], and elastocaloric materials[19], but extrinsic factors have not yet received as much attention in the barocaloric field.

Supplementary Note 6. Variable-temperature infrared spectroscopy

To further investigate conformational degrees of freedom that are present in the LT phase of $(NA)_2CuBr_4$ but not in the LT phase of $(DA)_2MnCl_4$, IR spectra were collected during heating for each compound from 5 K below the phase transition temperature to 5 K above the phase transition temperature. IR spectra were collected at 2 cm^{-1} resolution and averaged over 64 scans. The temperature was equilibrated for 2 min prior to each data collection.

The following IR bands were monitored: C–H stretching bands (2920 cm^{-1}, anti-symmetric; 2850 cm^{-1}, symmetric), for which blue shifts are correlated with an increase in the number of gauche C–C bonds and a change in chain packing[20]; CH_2 rocking (720 cm^{-1}) and bending (1470 cm^{-1}) bands, for which the factor group splitting can be used to indicate the presence of conformational disordering of long hydrocarbon chains in the 2-D perovskites[3,21–23]; CH_2 wagging bands (1370–1310 cm^{-1}), which provide characteristic signals of specific conformational defects in alkylammonium chains[22,24]; NH_3 bending bands (1590–1570 cm^{-1}, anti-symmetric; 1500–1480 cm^{-1}, symmetric), for which the broadening of symmetric bending modes—accompanied by the red-shifting of anti-symmetric bending modes—is associated with the reorientational motion of the chains within the halide pocket[3,21,25]; and CH_3 symmetric bending mode (1380 cm^{-1}), which is correlated with the change in inter-lamellar interactions within the organic bilayers and blue shifts during the transition[26]. The IR bands used for conformational analysis for $(DA)_2MnCl_4$ and $(NA)_2CuBr_4$ are summarized in Supplementary Table 13.

IR spectra of $(DA)_2MnCl_4$ and $(NA)_2CuBr_4$ are shown in Supplementary Fig. 37. For $(DA)_2MnCl_4$, CH_2 rocking and bending bands near 720 and 1470 cm^{-1} appear as doublets in the LT phase due to the factor group splitting that arises from directional, inter-chain interaction in the monoclinic unit cell[23]. This splitting disappears in the orthorhombic HT phase as a result of chain disordering. In $(NA)_2CuBr_4$, although the CH_2 rocking and bending bands appear at similar frequencies as to those of $(DA)_2MnCl_4$, the factor group splitting is not observed in either phase, presumably because the chains are arranged in a triclinic unit cell in the LT phase. Thus, these signals do not provide useful information about differences in the disordering processes of the alkylammonium chains. Both compounds display small blue shifts (2 cm^{-1}) for symmetric C–H stretching peaks after the order–disorder transition, which is consistent with increased disorder of the alkylammonium chains. For symmetric CH_3 bending bands, both compounds display blue shifts.

However, the degree to which the peak shifts is smaller for $(NA)_2CuBr_4$ ($\Delta v < 2$ cm^{-1}) than for $(DA)_2MnCl_4$ ($\Delta v = 4$ cm^{-1}). This result indicates that the local environments around the methyl ends are more similar in the LT and HT phases of $(NA)_2CuBr_4$ than in those of $(DA)_2MnCl_4$.

Notably, both compounds show pronounced differences in the progression of CH_2 wagging bands, as shown in Supplementary Fig. 37. In $(DA)_2MnCl_4$, CH_2 wagging bands associated with $\{gt_{2n+1}g'\}$ kink formation appear near 1310 and 1367 cm^{-1} in the HT phase. Note that these IR changes during the order–disorder transition are similar to those observed for $(C_{14})_2MnCl_4$ (ref. 22). In contrast, the IR spectrum of the LT phase of $(NA)_2CuBr_4$ shows a shoulder peak at 1360 cm^{-1} (near the CH_3 symmetric bending peak at 1378 cm^{-1}) and a peak near 1340 cm^{-1}, which indicate the presence of $\{gt_{2n+1}g'\}$ kink and end-gauche conformations, respectively. The end-gauche conformer in $(NA)_2CuBr_4$ is further supported by the small shift in symmetric CH_3 bending peaks during the phase transition and the higher frequency of the LT phase peak (1378 cm^{-1}) compared to $v_s(CH_3)_{bending}$ of $(DA)_2MnCl_4$ at 1375 cm^{-1} in the LT phase. Overall, these data suggest that conformational disorder associated with a kink conformation is present in $(NA)_2CuBr_4$ even before the transition. In the HT phase, this shoulder peak disappears while another CH_2 wagging band, also associated with a new kink formation[22], emerges at 1312 cm^{-1}.

Although the spectral changes we observe for both compounds are consistent with those reported for other 2-D metal–halide perovskites[3,21,22,25], we note that accurate assignments and quantitative interpretation of IR spectra, particularly of CH_2 wagging bands, are challenging, because the signals are relatively weak, sensitive to defects, and coupled with the internal modes of chain ends and headgroups[22]. To more definitively assign specific chain conformations, particularly those for $(NA)_2CuBr_4$, further investigations using normal-mode calculations and other complementary techniques, such as sum frequency generation vibrational spectroscopy[27,28], or quasi-elastic neutron scattering[4], will be required.

Comparisons of IR spectra in the region associated with NH_3 bending modes provide insights into how charge-assisted H-bond interactions differ between the two compounds. The anti-symmetric NH_3 bending mode of $(NA)_2CuBr_4$ appears at 1570 cm^{-1} in the LT phase which is 15 cm^{-1} lower than that of $(DA)_2MnCl_4$ (1585 cm^{-1}) (Supplementary Fig. 37), as well as lower than that reported for other M–Cl perovskite analogs (Cu–Cl, 1583 cm^{-1}; Cd–Cl, 1589 cm^{-1})[3,21,22,25]. In $(NA)_2CuBr_4$, the position and shape of the $v_{as}(NH_3)_{bending}$ peak does not change after the transition, whereas that of $(DA)_2MnCl_4$ red shifts ($\Delta v = -6$ cm^{-1}) and broadens. This suggests that H bonds to the ammonium headgroups of the DA chains weaken after the transition to the HT phase—leading to increased rotational degrees of freedom for the headgroups—while the local environments around the headgroups of the NA chains remain nearly unchanged after the transition. Similar effects are observed for the NH_3 symmetric bending modes, with the $v_s(NH_3)_{bending}$ peak of $(NA)_2CuBr_4$ red shifted by ~16 cm^{-1} compared to that of $(DA)_2MnCl_4$ in the LT phase and transition-induced peak broadening only observed for $(DA)_2MnCl_4$.

Taken together, these IR spectra indicate that (*i*) a noticeable degree of conformational disorder is present in the LT phase of $(NA)_2CuBr_4$ and (*ii*) the conformations accessed by—and local environments around—the alkylammonium chains are more similar before and after the phase transition in $(NA)_2CuBr_4$ than in $(DA)_2MnCl_4$. This is consistent with the single-crystal structures of each compound, which show a higher degree of disorder present in the NA chains of the LT

phase of $(NA)_2CuBr_4$ than in the DA chains of $(DA)_2MnCl_4$ but similar levels of disorder present in both HT phases. In addition, we note that the lower entropy change associated with the order-disorder transition of $(NA)_2CuBr_4$ is also consistent with the previously reported thermal data of $(C_n)_2CuBr_4$ (n = 11–16)[29], which shows that the transition entropies of 2-D Cu–Br perovskites are only 40–60% of those reported in Cu–Cl and Mn–Cl analogs. Although the origin of entropy changes for order-disorder transitions in 2-D Cu–Br with longer alkylammonium chains has not yet been investigated, we suspect that similar factors as to those described here are responsible for the lower transition entropies of other 2-D Cu–Br perovskites.

Supplementary Note 7. Environmental impact of cooling

According to the International Energy Agency, the total global electricity consumption in 2018 was 22,315 TWh. The International Institute of Refrigeration (IIR) estimates that the cooling sector—which includes refrigeration, air-conditioning, and heat pumps—consumes ~20% of the electricity used worldwide[30]. From these values, we estimated that nearly 4,400 TWh of electricity is used to power refrigerators, air conditioners, and heat pumps annually. The IIR also estimated that 2.34 Gt of CO_2 was released in 2014 to produce the electricity used by the cooling sector[31]. Other greenhouse gases released during electricity generation also contribute to the total indirect emissions associated with cooling, with 0.24 $GtCO_2eq$ and 0.02 $GtCO_2eq$ of CH_4 and N_2O emissions, respectively. This leads to total indirect emissions of at least 2.6 $GtCO_2eq$. In addition to these indirect emissions, the IIR estimates that 1.53 $GtCO_2eq$ of fluorocarbon refrigerants—including chlorofluorocarbons, hydrochlorofluorocarbons, and hydrofluorocarbons—were directly emitted in 2014 by the cooling sector[31]. The cumulative direct and indirect emissions by the cooling sector are thus 4.14 $GtCO_2eq$, which accounted for 7.8% of global greenhouse gas emissions in 2014.

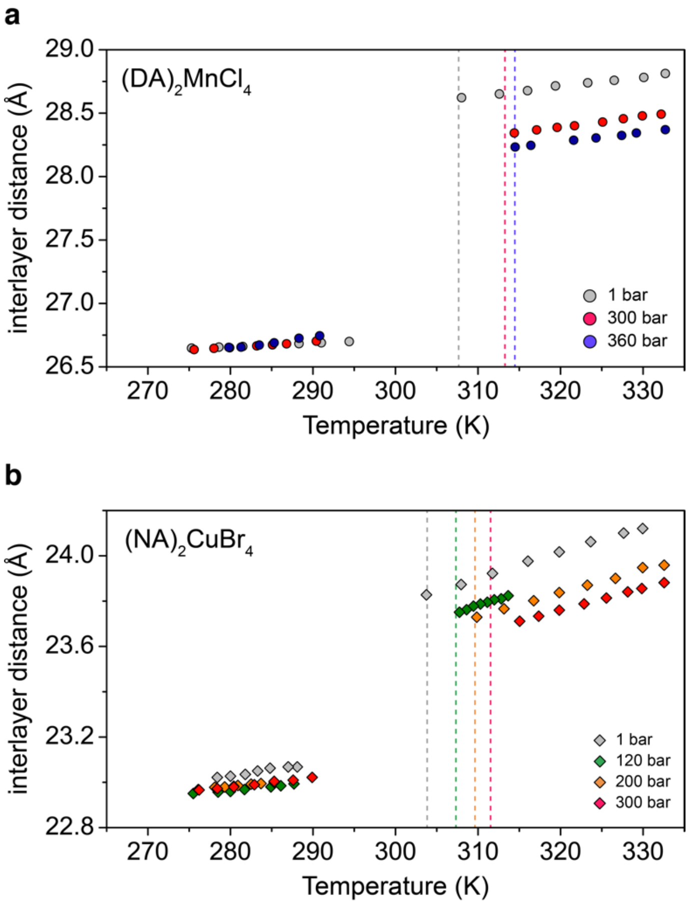

Supplementary Fig. 1 | a, **b**, Temperature dependence of interlayer distances between inorganic sheets for (**a**) $(DA)_2MnCl_4$ and (**b**) $(NA)_2CuBr_4$ obtained from isobaric PXRD experiments during cooling. The interlayer distance at T_{tr} for the LT and HT phases was extrapolated using the temperature dependence of the interlayer distance of each phase.

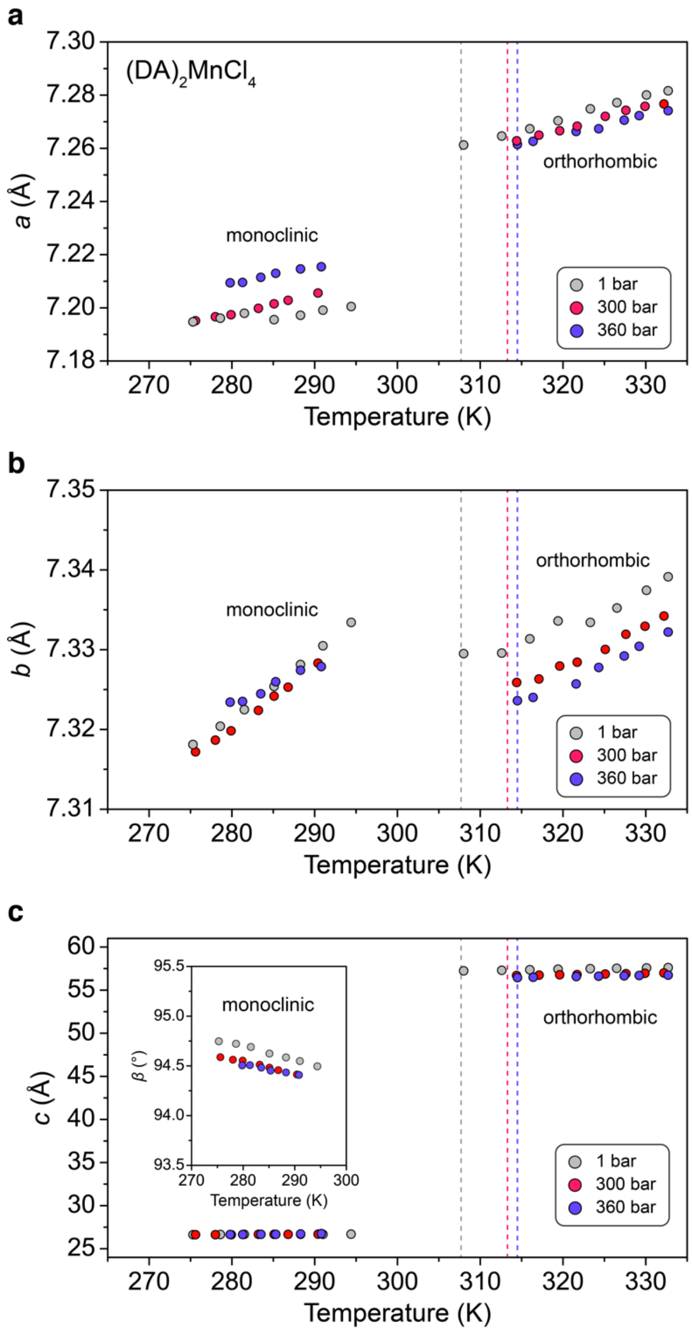

Supplementary Fig. 2 | Temperature dependence of unit cell parameters for $(DA)_2MnCl_4$ obtained from isobaric PXRD experiments during cooling.

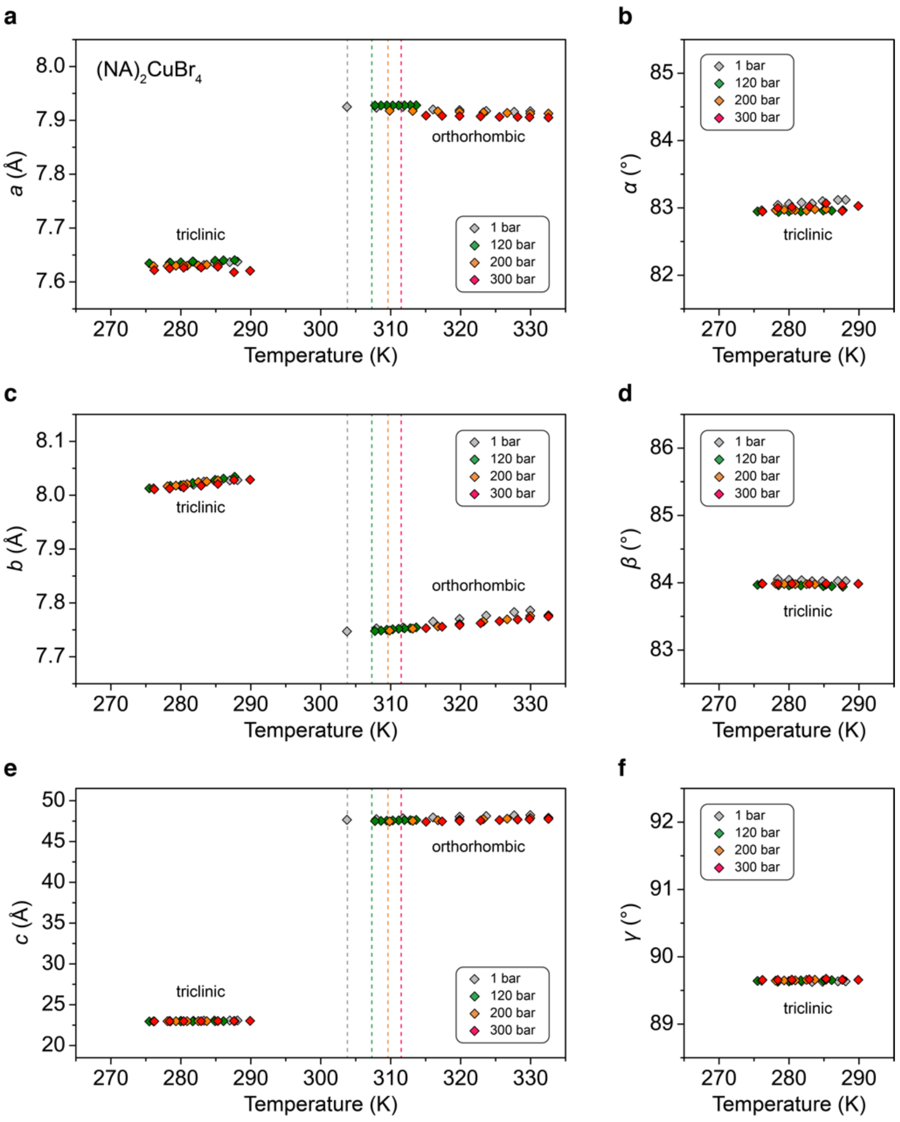

Supplementary Fig. 3 | Temperature dependence of unit cell parameters for $(NA)_2CuBr_4$ obtained from isobaric PXRD experiments during cooling.

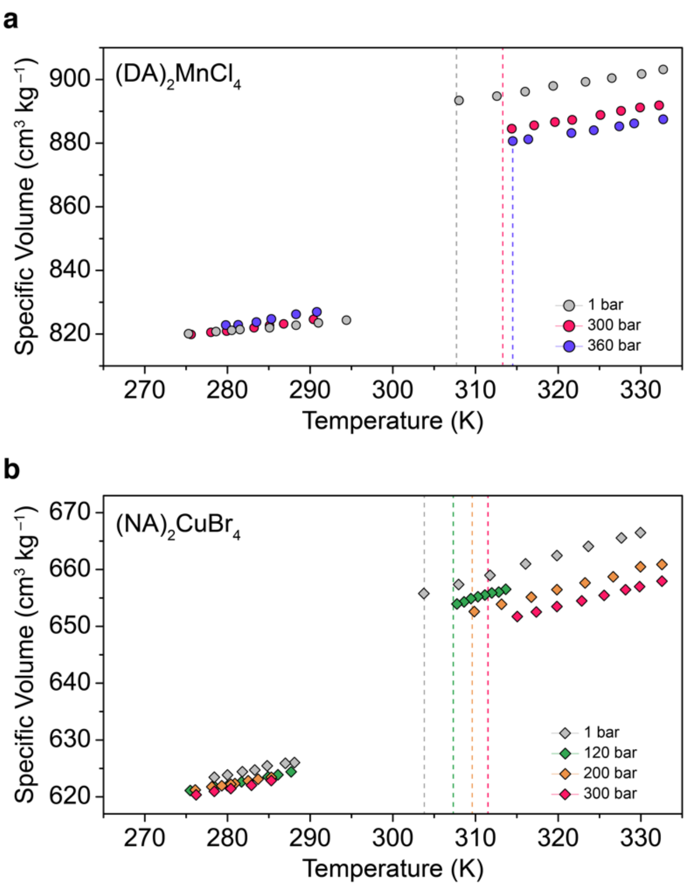

Supplementary Fig. 4 | a, **b**, Temperature dependence of specific volume across the order-disorder transition for (**a**) $(DA)_2MnCl_4$ and (**b**) $(NA)_2CuBr_4$ obtained from powder X-ray diffraction data collected during isobaric cooling. The transition temperature at each pressure is indicated by a vertical dotted line. Thermal expansion coefficients determined from this volume data are listed in Supplementary Table 9.

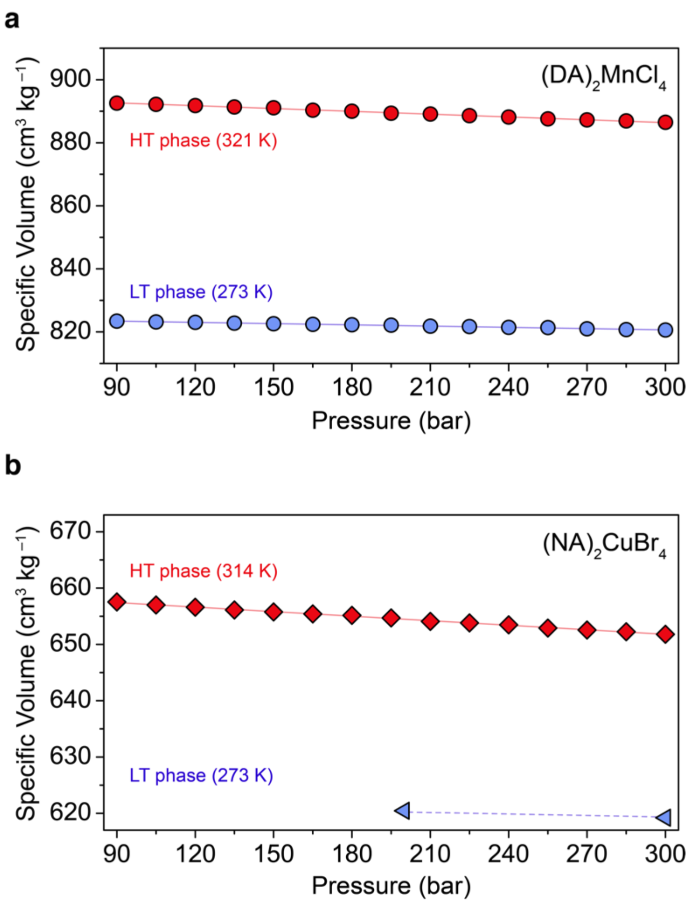

Supplementary Fig. 5 | a, **b**, Pressure dependence of specific volume above and below the phase transition temperature for (**a**) $(DA)_2MnCl_4$ and (**b**) $(NA)_2CuBr_4$ obtained from isothermal PXRD experiments. For $(DA)_2MnCl_4$, the LT and HT phase data was collected during compression and decompression, respectively. For $(NA)_2CuBr_4$, the HT phase data was collected during compression, and specific volumes for the LT phase (triangles) are shown using the isobaric data in Supplementary Fig. 4. Isothermal compressibility values calculated from this data are listed in Supplementary Table 9.

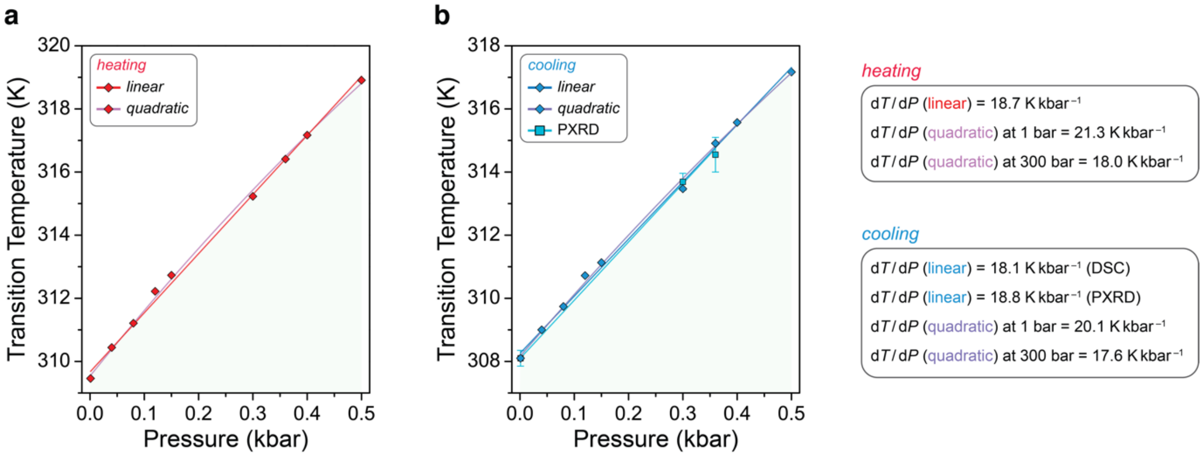

Supplementary Fig. 6 | a, **b**, Pressure dependence of the order-disorder transition temperature, T_{tr}, as determined by HP-DSC (diamonds) and PXRD (squares) for $(DA)_2MnCl_4$ during (**a**) heating and (**b**) cooling. Both linear and quadratic models provided a reasonably good fit to the data. Barocaloric coefficients (dT/dP) are summarized in Supplementary Table 8. Since the goal of these experiments was to determine the pressure dependence of T_{tr}, rather than the absolute values of T_{tr}, the transition temperatures determined via HP-DSC (Setaram DSC, 300–500 bar) and PXRD were normalized by measuring T_{tr} at ambient pressure and setting this value to be equal to T_{tr} determined by HP-DSC (Netzsch DSC, 1–150 bar) at ambient pressure. Offsets determined in this manner were less than 1.1 K.

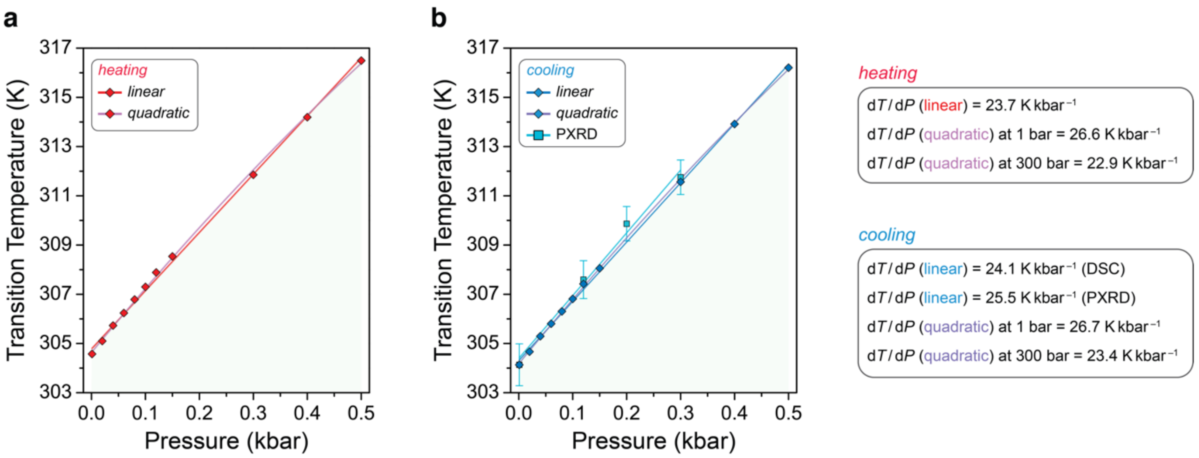

Supplementary Fig. 7 | a, **b**, Pressure dependence of T_{tr} as determined by HP-DSC (diamonds) and PXRD (squares) for $(NA)_2CuBr_4$ during (**a**) heating and (**b**) cooling. Both linear and quadratic models provided a reasonably good fit to the data. Barocaloric coefficients (dT/dP) are summarized in Supplementary Table 8. Since the goal of these experiments was to determine the pressure dependence of T_{tr}, rather than the absolute values of T_{tr}, the transition temperatures determined via HP-DSC (Setaram DSC, 300–500 bar) and PXRD were normalized by measuring T_{tr} at ambient pressure and setting this value to be equal to T_{tr} determined by HP-DSC (Netzch DSC, 1–150 bar) at ambient pressure. Offsets determined in this manner were less than 1.2 K.

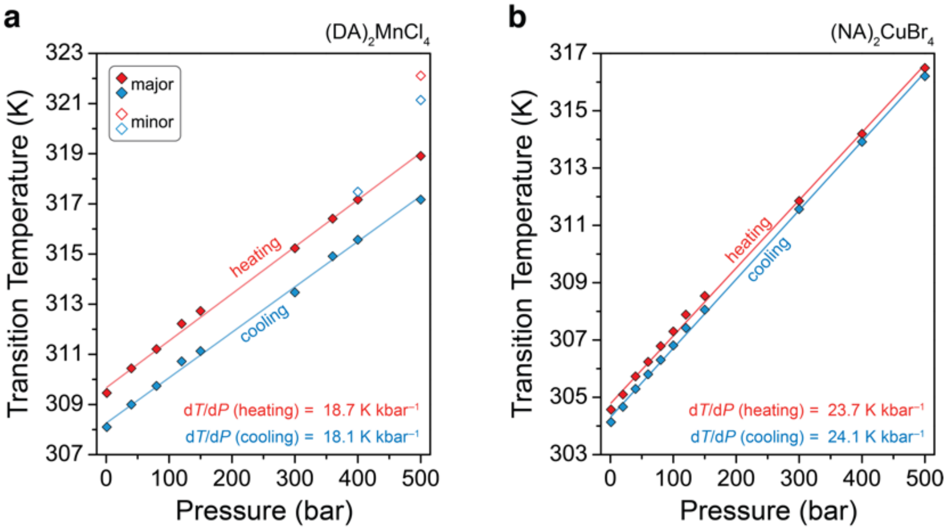

Supplementary Fig. 8 | a, **b**, Pressure dependence of T_{tr} as determined by HP-DSC for (**a**) $(DA)_2MnCl_4$ and (**b**) $(NA)_2CuBr_4$. Phase boundary and barocaloric coefficient were determined through a linear fit, only using the T_{tr} value for major transitions. Barocaloric coefficients are summarized in Supplementary Table 8.

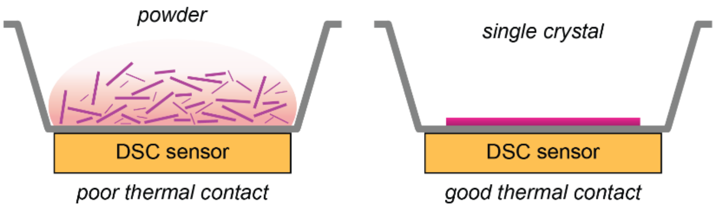

Supplementary Fig. 9 | Illustration of powder and single-crystal samples used for isobaric HP-DSC experiments. Owing to their layered nature, powder samples of 2-D perovskites contain highly anisotropic platelike particles, which tend—at least in their typical bulk form—to have relatively poor thermal contact in DSC pans. Large single-crystal samples, however, can be tightly placed on the bottom of the pan to maximize thermal contact.

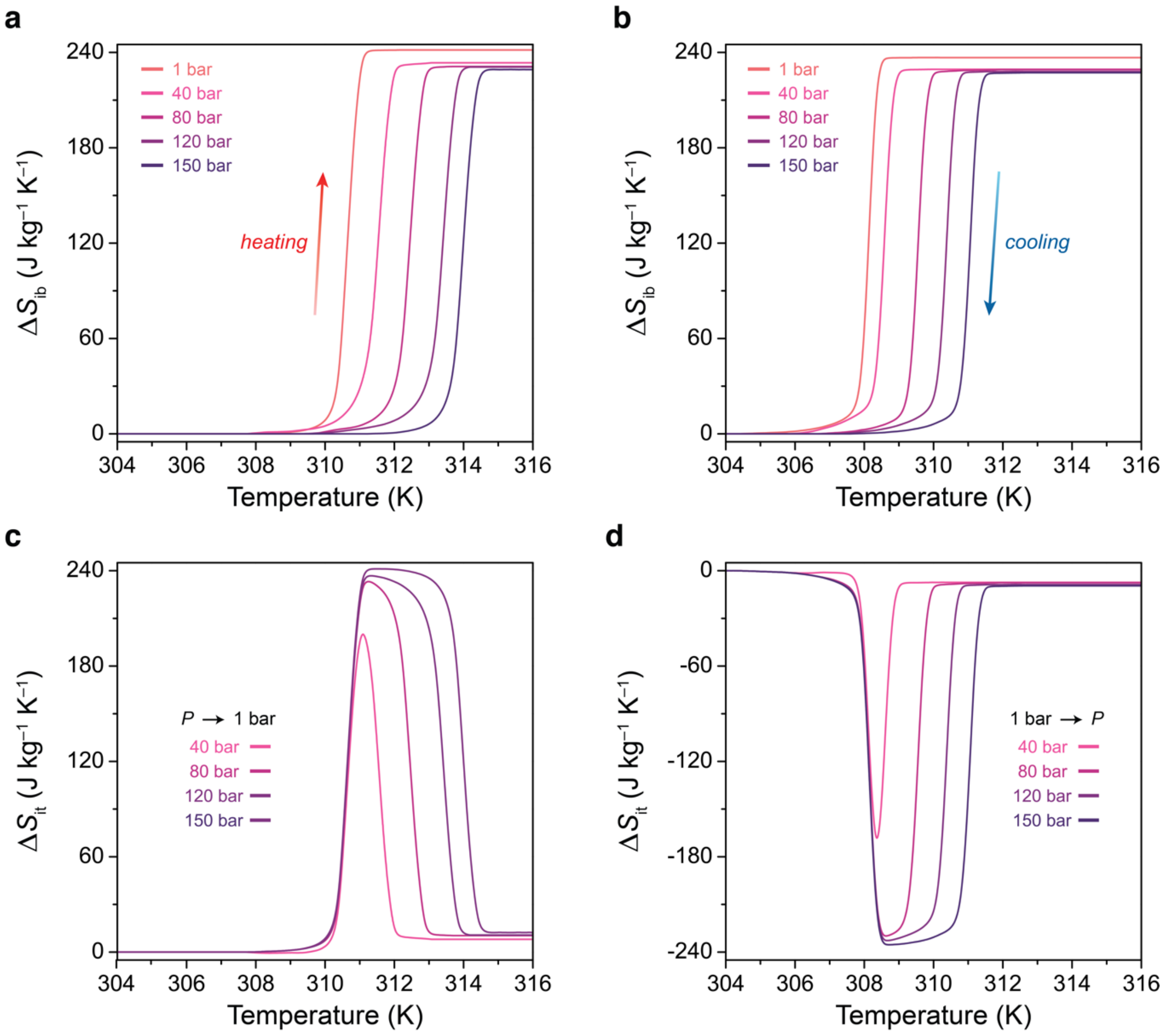

Supplementary Fig. 10 | a, **b**, Isobaric entropy changes (ΔS_{ib}) associated with the phase transition of a single-crystal sample of $(DA)_2MnCl_4$, as a function of temperature in the pressure range of 1 bar to 150 bar during (**a**) heating and (**b**) cooling. **c**, **d**, Isothermal entropy changes (ΔS_{it}), calculated by the quasi-direct method, for (**c**) decompression to ambient pressure and (**d**) compression from ambient pressure, obtained from heating and cooling data, respectively.

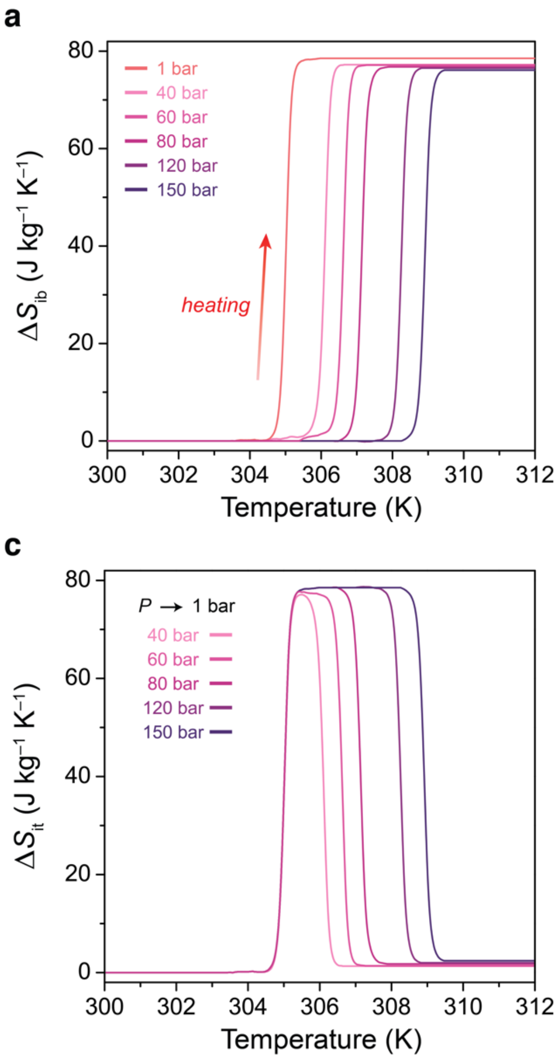

Supplementary Fig. 11 | a, **b**, Isobaric entropy changes (ΔS_{ib}) associated with the phase transition of a single-crystal sample of $(NA)_2CuBr_4$, as a function of temperature in the pressure range of 1 bar to 150 bar during (**a**) heating and (**b**) cooling. **c**, **d**, Isothermal entropy changes (ΔS_{it}), calculated by the quasi-direct method, for (**c**) decompression to ambient pressure and (**d**) compression from ambient pressure, obtained from heating and cooling data, respectively.

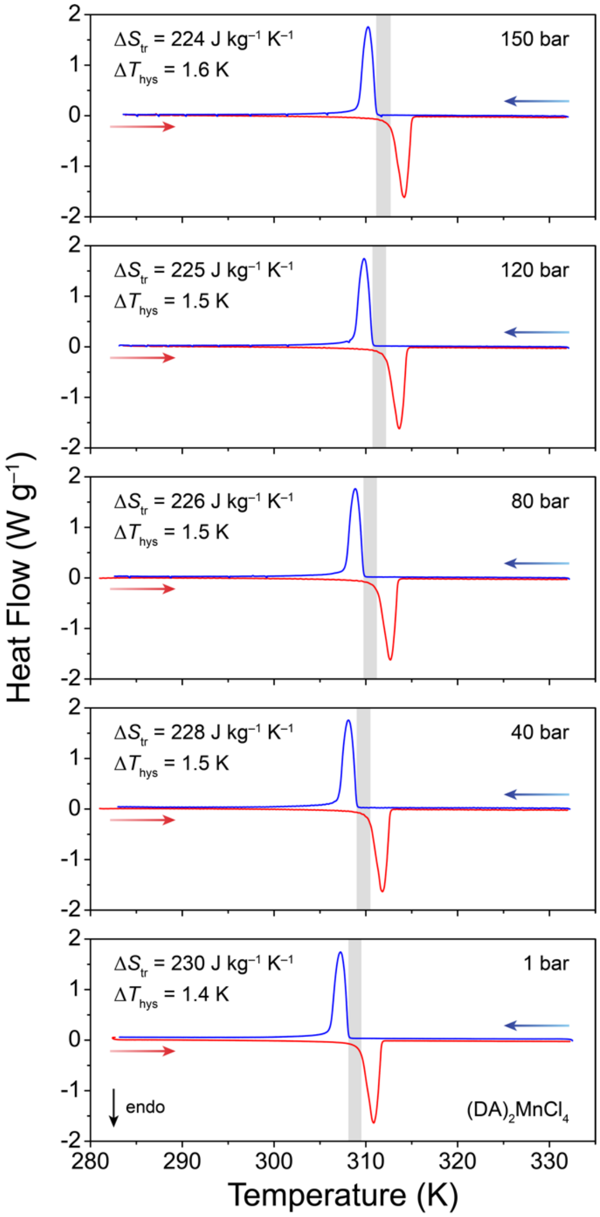

Supplementary Fig. 12 | Differential scanning calorimetry (DSC) measurements under applied hydrostatic pressure for a powder sample of $(DA)_2MnCl_4$ with heating and cooling rates of 2 K min^{-1}. Entropies of transition (ΔS_{tr}) are listed, along with thermal hysteresis (ΔT_{hys}), which is also indicated by the vertical grey bars. Note that ΔT_{hys} is calculated as the difference between heating and cooling transition onset temperatures, with $\Delta T_{hys} = T_{tr,heating} - T_{tr,cooling}$. The instrument was cooled using a low-temperature nitrogen gas, and this process introduced small, periodic blips in cooling traces of 80, 120, and 150 bar data. Note that, after a pressure change at 280 K, an additional heating segment of 1–2 min was introduced to stabilize the baseline and scan rate.

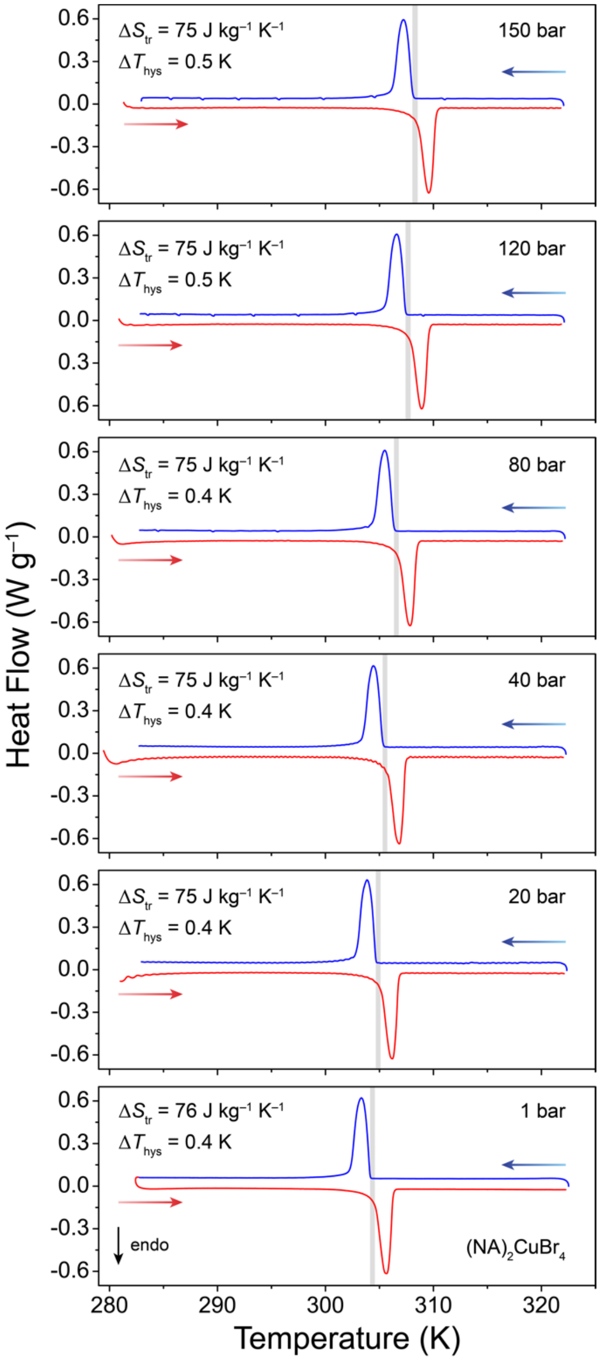

Supplementary Fig. 13 | DSC measurements under applied hydrostatic pressure for a powder sample of $(NA)_2CuBr_4$ with heating and cooling rates of 2 K min^{-1}. ΔS_{tr} values are shown, with ΔT_{hys} indicated by the vertical grey bars. Note that ΔT_{hys} is calculated as the difference between heating and cooling transition onset temperatures, with $\Delta T_{hys} = T_{tr,\ heating} - T_{tr,\ cooling}$. The instrument was cooled using a low-temperature nitrogen gas, and this process introduced small, periodic blips in cooling traces of 80, 120, and 150 bar data. Note that, after a pressure change at 280 K, an additional heating segment of 1–2 minute was introduced to stabilize the baseline and scan rate.

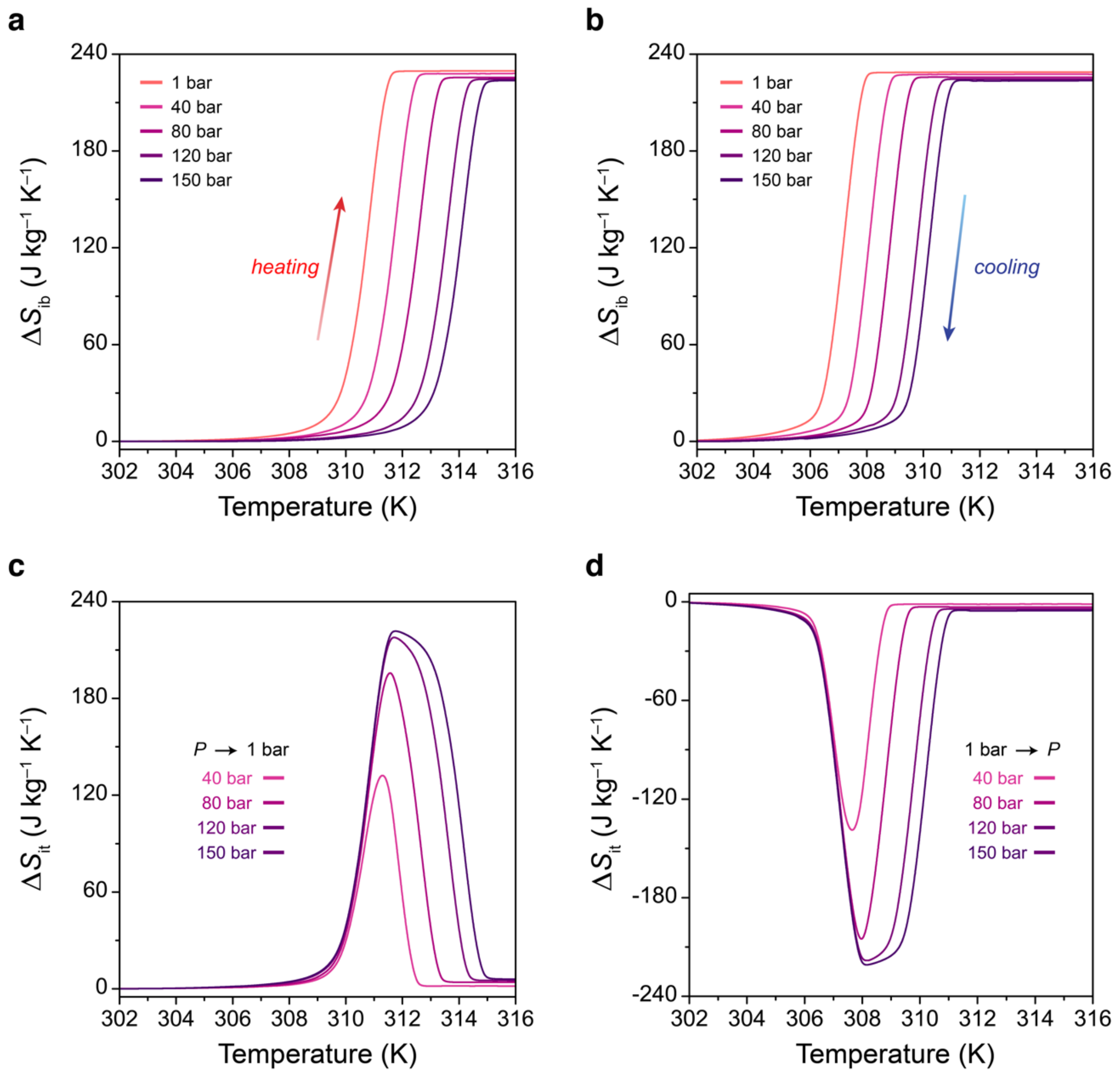

Supplementary Fig. 14 | a, **b**, Isobaric entropy changes (ΔS_{ib}) associated with the phase transition of a powder sample of $(DA)_2MnCl_4$, as a function of temperature in the pressure range of 1 bar to 150 bar during (**a**) heating and (**b**) cooling. **c**, **d**, Isothermal entropy changes (ΔS_{it}), calculated by the quasi-direct method, for (**c**) decompression to ambient pressure and (**d**) compression from ambient pressure, obtained from heating and cooling data, respectively.

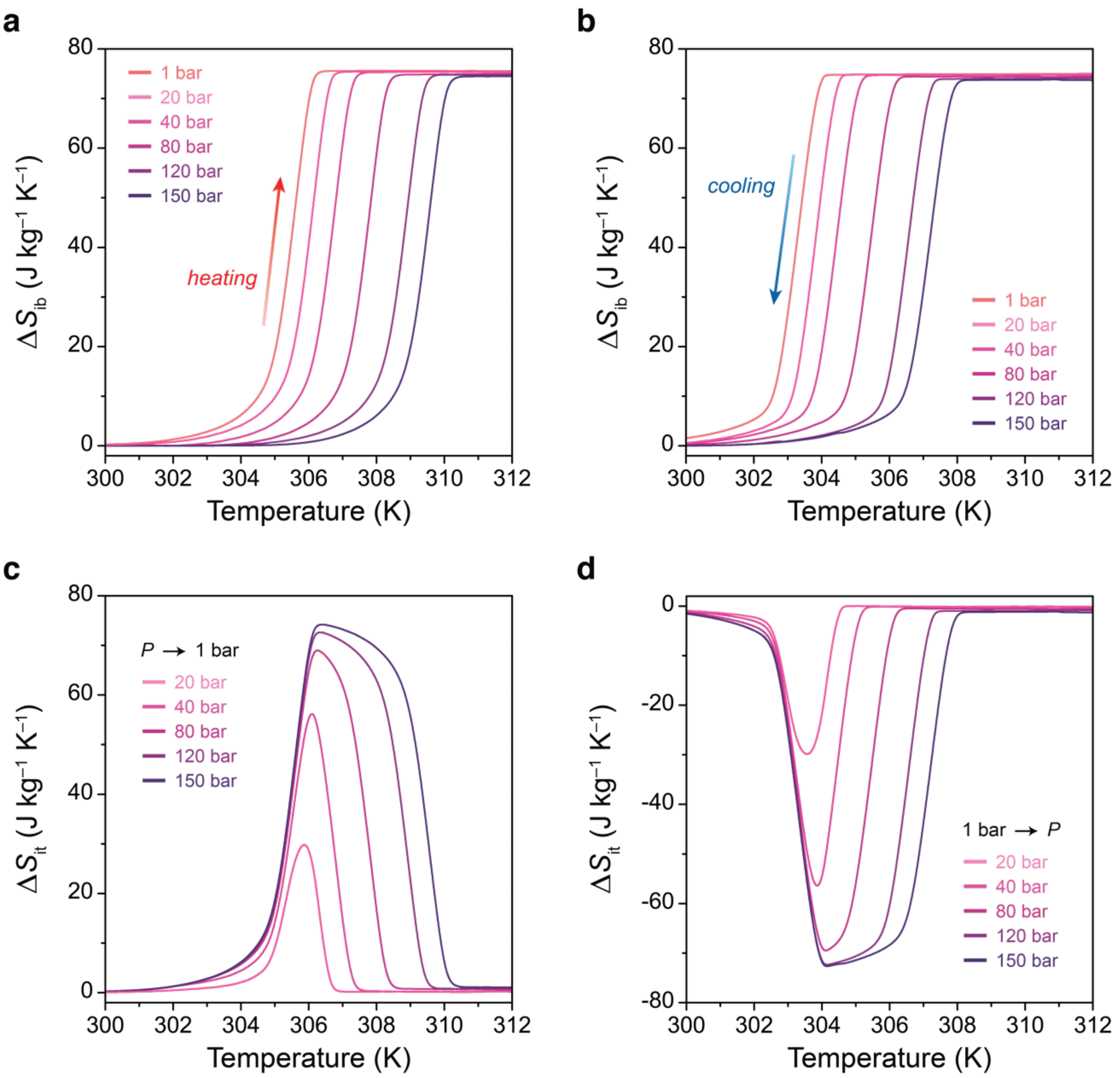

Supplementary Fig. 15 | a, **b**, Isobaric entropy change (ΔS_{ib}) associated with the phase transition of a powder sample of $(NA)_2CuBr_4$, as a function of temperature in the pressure range of 1 bar to 150 bar on (**a**) heating and (**b**) cooling. **c**, **d**, Isothermal entropy changes (ΔS_{it}), calculated by the quasi-direct method, for (**c**) decompression to ambient pressure and (**d**) compression from ambient pressure, obtained from heating and cooling data, respectively.

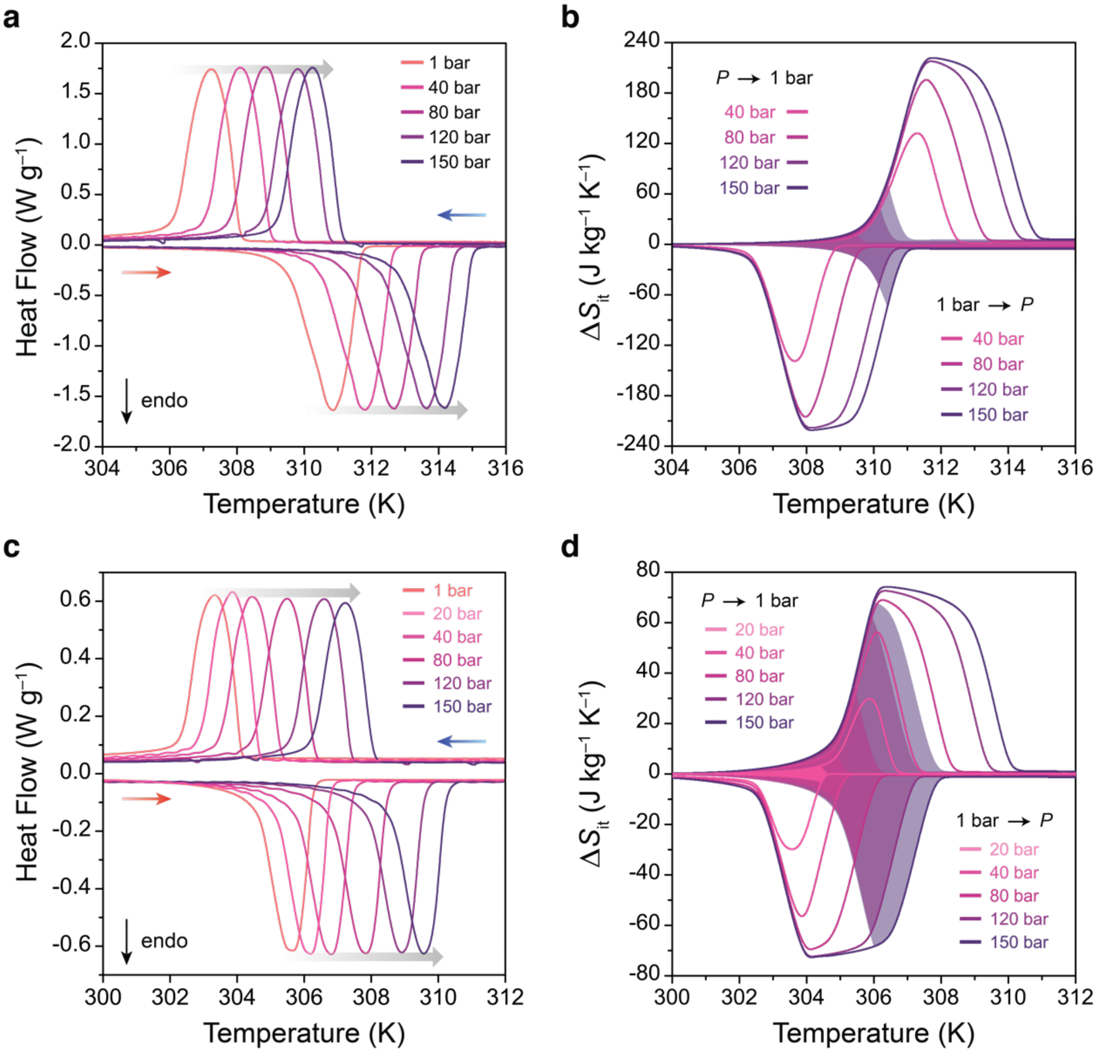

Supplementary Fig. 16 | a, **c**, DSC measurements under applied hydrostatic pressure for powder samples of (**a**) $(DA)_2MnCl_4$ and (**c**) $(NA)_2CuBr_4$ up to 150 bar, with heating and cooling rates of 2 K min^{-1}. Helium was used as the pressure transmitting medium. **b**, **d**, Isothermal entropy changes (ΔS_{it}) calculated by the quasi-direct method for (**b**) $(DA)_2MnCl_4$ and (**d**) $(NA)_2CuBr_4$. The shaded area indicates the reversible ΔS_{it} within this pressure range. Maximum reversible isothermal entropy changes plotted as a function of operating pressure are shown in Figs. 5c,f.

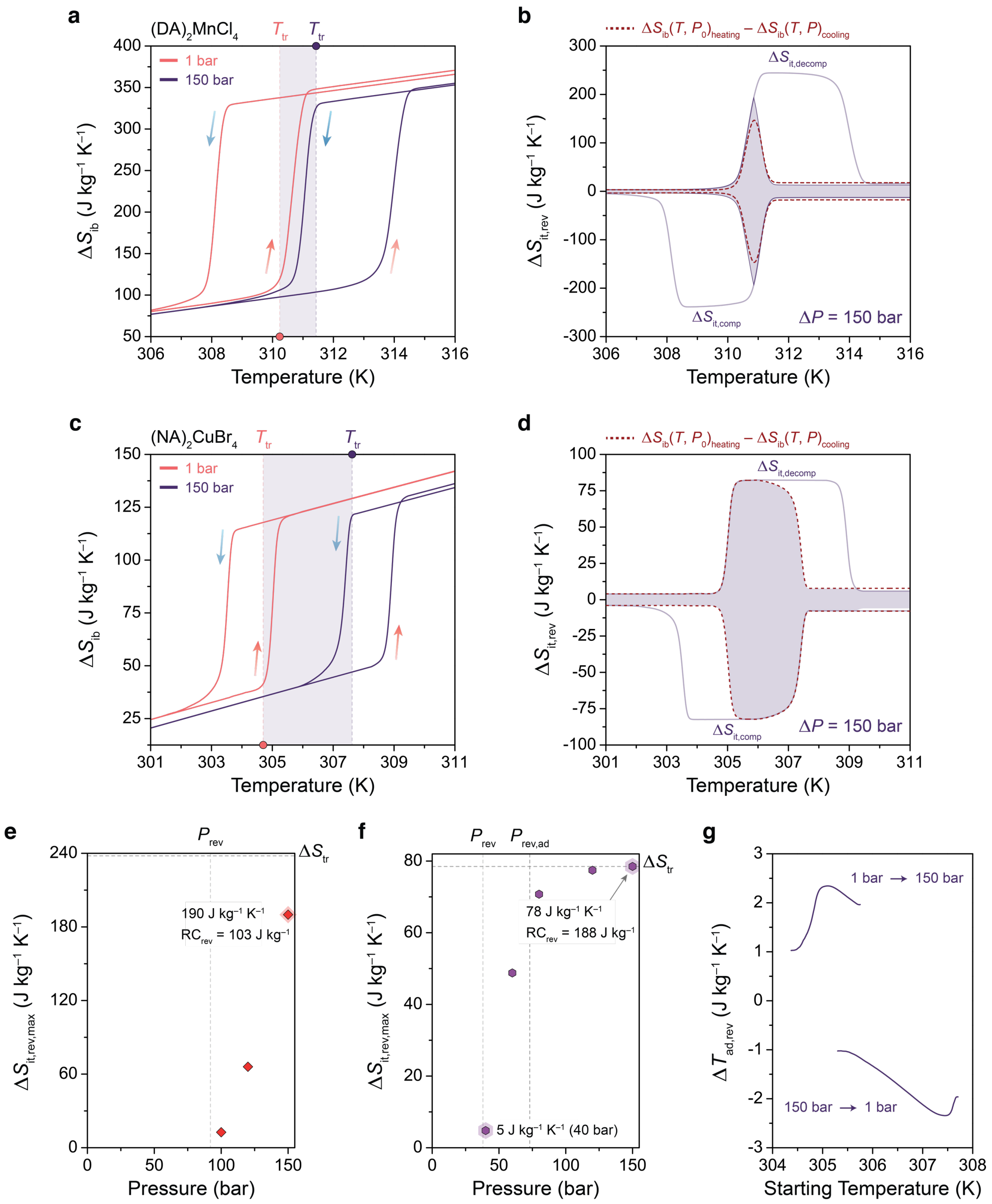

Supplementary Fig. 17 | Reversible barocaloric effects at low pressures. **a**, **c**, Isobaric entropy changes (ΔS_{ib}) during heating and cooling at 1 and 150 bar for single-crystal samples of (**a**) $(DA)_2MnCl_4$ and (**c**) $(NA)_2CuBr_4$. The entropy curves were calculated from the isobaric HP-DSC data shown in Figures 3a and 3d. Transition onset temperatures are marked with orange dots for the 1-bar heating curves and purple dots for the 150-bar cooling curves. The temperature range over which reversible isothermal entropy changes can be achieved is indicated by the shaded area. **b**, **d**, Reversible isothermal entropy changes ($\Delta S_{it,rev}$) at a 150-bar operating pressure calculated from the single-crystal HP-DSC data for (**b**) $(DA)_2MnCl_4$ and (**d**) $(NA)_2CuBr_4$, with the purple shaded area and the brown dashed line obtained from the overlap method and from the subtraction method, respectively. **e**, **f**, Maximum reversible isothermal entropy changes ($\Delta S_{it,rev,max}$) are shown as a function of pressure for single-crystal samples of (**e**) $(DA)_2MnCl_4$ and (**f**) $(NA)_2CuBr_4$. **g**, Reversible adiabatic temperature changes ($\Delta T_{ad,rev}$) at 150 bar as a function of starting temperature for a single-crystal sample of $(NA)_2CuBr_4$.

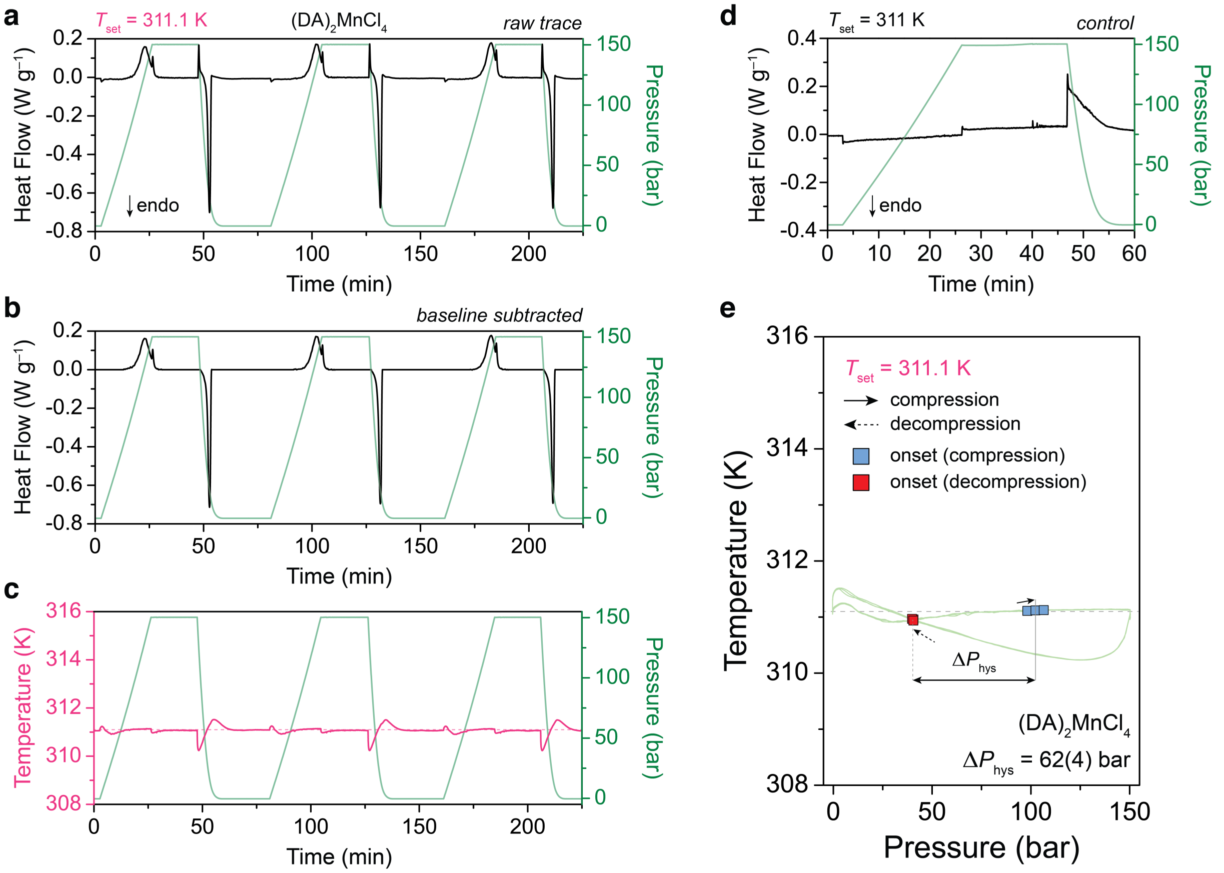

Supplementary Fig. 18 | Quasi-isothermal HP-DSC experiments for (DA)$_2$MnCl$_4$. Direct evaluation of pressure hysteresis, ΔP_{hys}, through quasi-isothermal DSC experiments. **a**–**c**, Heat flow signals and temperature were measured as a function of time during three cycles of applying and removing a hydrostatic pressure of 150 bar at 311.1 K. Note that the pressure was linearly increased at a rate of 6 bar min^{-1} and asymptotically decreased at an average rate of 13 bar min^{-1}. In addition to the pressure-induced phase transition, the compression and decompression of the He pressure transmitting medium is also associated with thermal changes that lead to DSC heat signals. While compression led to only small changes in the baseline due to the slow compression rate, the fast decompression from 150 bar induced a sharp exothermic peak as heat flows from the sample into the cooling pressure-transmitting medium. **d**, To distinguish the sample heat flows from those associated with He, experiments with a control sample ($(C_{12}H_{25}NH_3)_2MnCl_4$), which does not undergo any phase transitions until above 330 K, were carried out at 311 K. From this control experiment, the baseline features associated with He compression and decompression were modeled and subtracted from the raw DSC traces. The panels correspond to (**a**) the raw heat flow traces, (**b**) the baseline-subtracted heat flow signals, and (**c**) temperature as a function of time and pressure. **e**, The pressure and temperature of the sample during the pressure cycling experiment is plotted. Onset pressures and temperatures for compression-induced exotherms and decompression-induced endotherms are shown as square and diamond symbols. Note that the difference in temperatures at the onset pressures for the compression- and decompression-induced transitions is smaller than 0.2 K. The measured ΔP_{hys} of 62(4) agrees well with the ΔP_{hys} values of 70 bar predicted from isobaric experiments. The results are summarized in Supplementary Table 11.

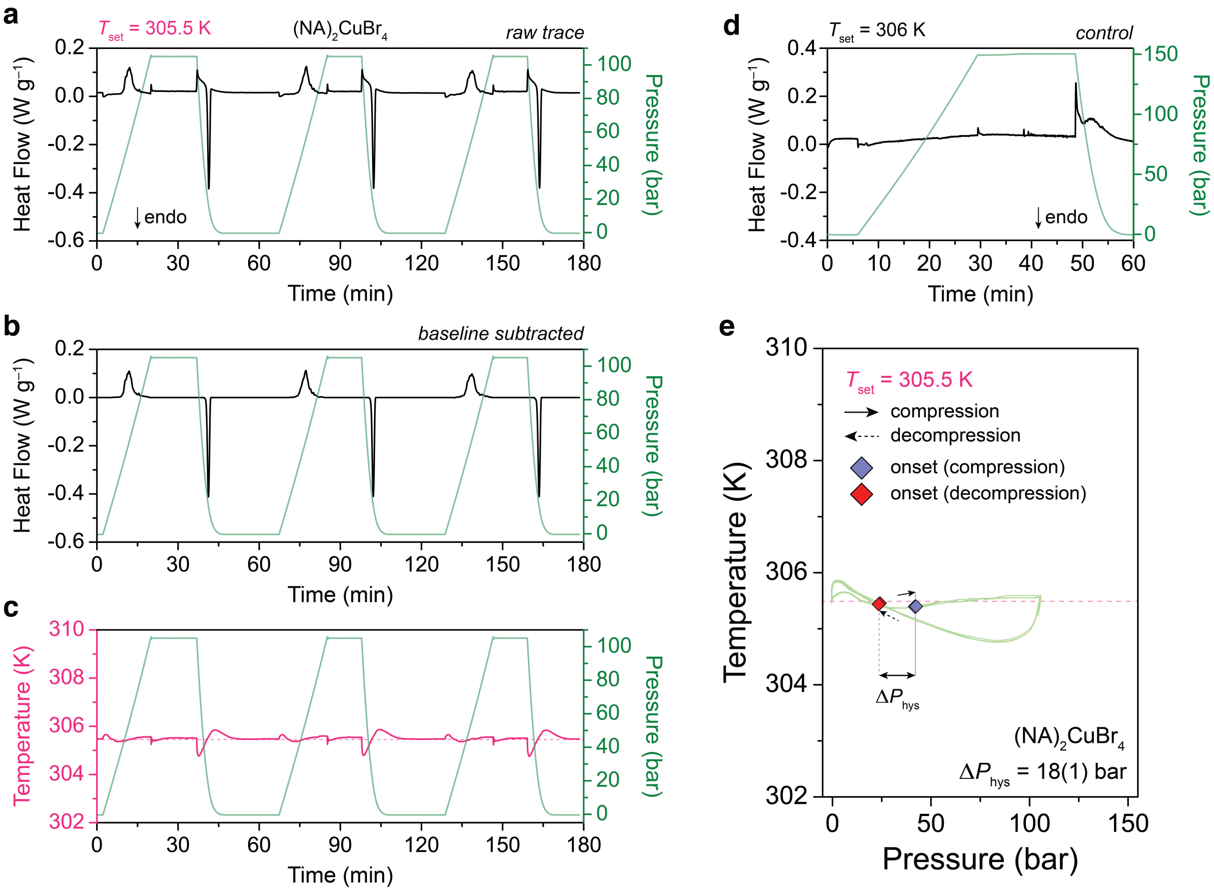

Supplementary Fig. 19 | Quasi-isothermal HP-DSC experiments for (NA)$_2$CuBr$_4$. Direct evaluation of ΔP_{hys} through quasi-isothermal DSC experiments. **a**–**c**, Heat flow signals and temperature were measured as a function of time during three cycles of applying and removing a hydrostatic pressure of 105 bar at 305.5 K. Note that the pressure was linearly increased at a rate of 6 bar min^{-1} and asymptotically decreased at an average rate of 13 bar min^{-1}. In addition to the pressure-induced phase transition, the compression and decompression of the He pressure transmitting medium is also associated with thermal changes that lead to DSC heat signals. While compression led to only small changes in the baseline due to the slow compression rate, the fast decompression from 150 bar induced a sharp exothermic peak as heat flows from the sample into the cooling pressure-transmitting medium. **d**, To distinguish the sample heat flows from those associated with He, experiments with a control sample (C$_{12}$H$_{25}$NH$_3$)$_2$MnCl$_4$, which does not undergo any phase transitions until above 330 K, were carried out at 306 K. From these control experiments, the baseline features associated with He compression and decompression were modeled and subtracted from the raw DSC traces. The panels correspond to (**a**) the raw heat flow traces, (**b**) the baseline-subtracted heat flow signals, and (**c**) temperature as a function of time and pressure. **e**, The pressure and temperature of the sample during the pressure cycling experiment is plotted. Onset pressures and temperatures for compression-induced exotherms and decompression-induced endotherms are shown as square and diamond symbols. Note that the difference in temperatures at the onset pressures for the compression- and decompression-induced transitions is smaller than 0.1 K. The measured ΔP_{hys} of 18(1) bar agree well with the ΔP_{hys} values of 17 bar predicted from isobaric experiments. The results are summarized in Supplementary Table 11.

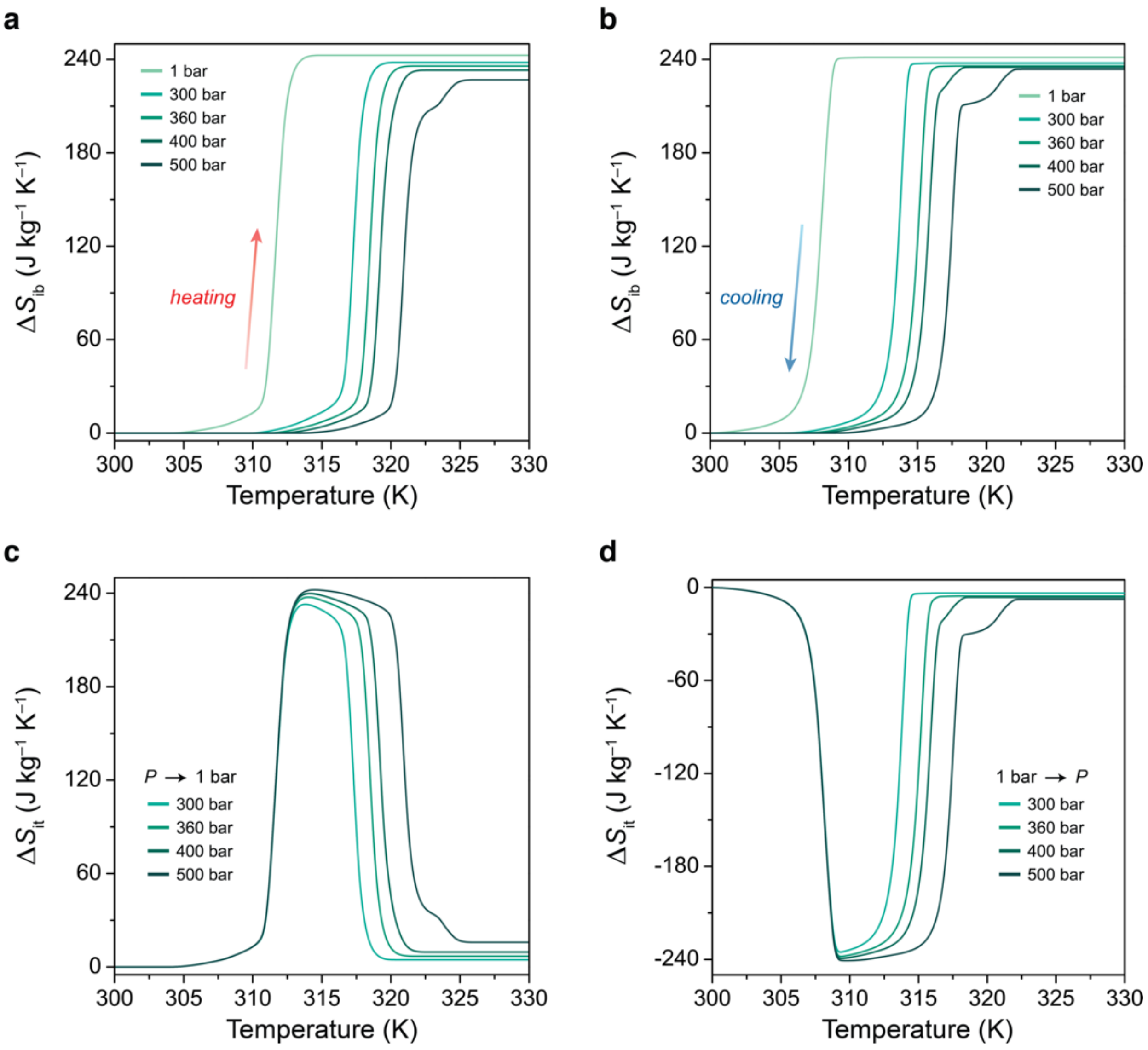

Supplementary Fig. 20 | a, **b**, Isobaric entropy changes (ΔS_{ib}) associated with the phase transition of a powder sample of $(DA)_2MnCl_4$ as a function of temperature in the pressure range of 1 bar to 500 bar during (**a**) heating and (**b**) cooling. **c**, **d**, Isothermal entropy changes (ΔS_{it}), calculated by the quasi-direct method, for (**c**) decompression to ambient pressure and (**d**) compression from ambient pressure, which are obtained from heating and cooling data, respectively. The integration ranges were set to include the minor transitions that occur at pressures above 400 bar.

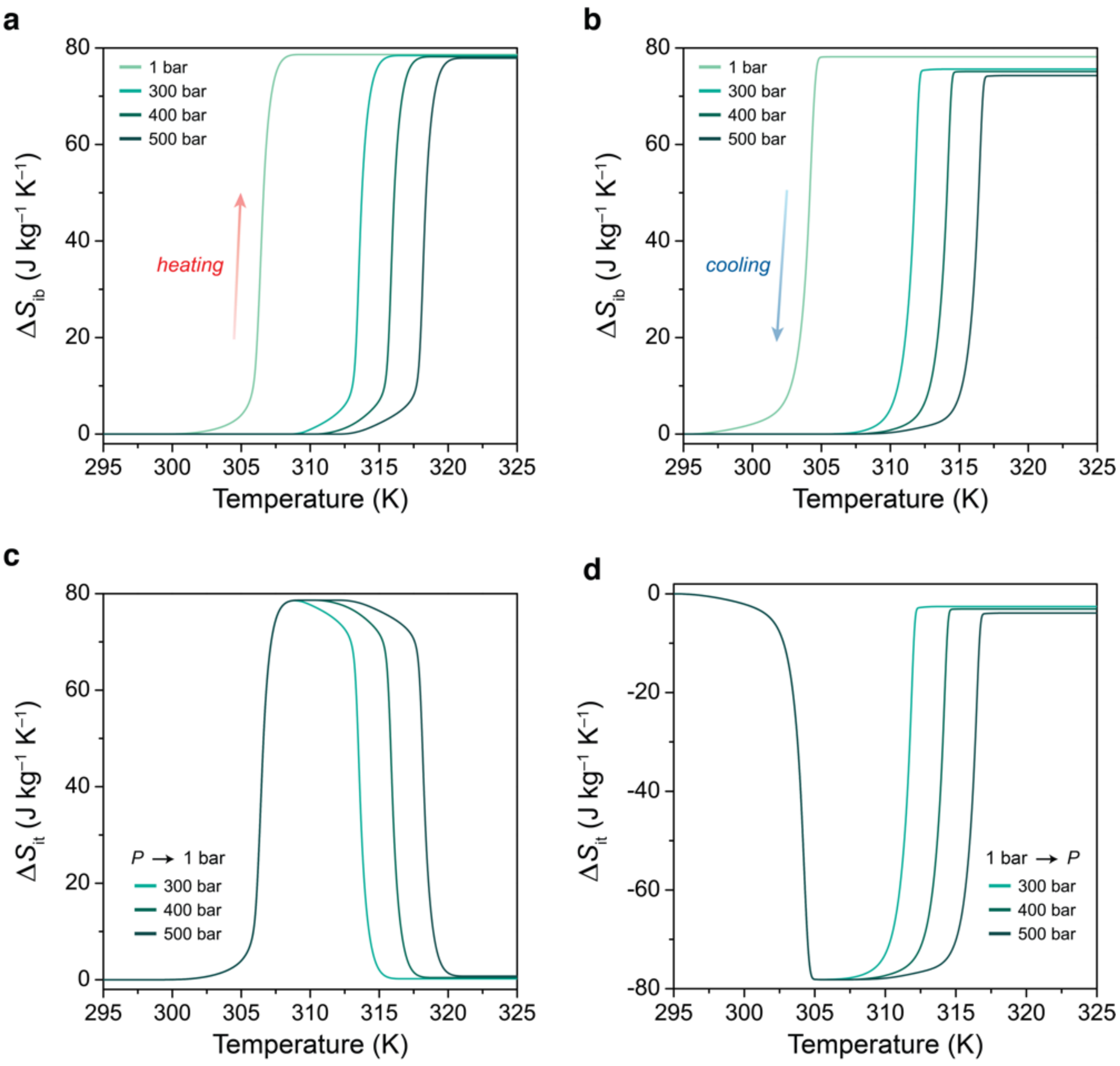

Supplementary Fig. 21 | a, **b**, Isobaric entropy changes (ΔS_{ib}) associated with the phase transition of a powder sample of $(NA)_2CuBr_4$ as a function of temperature in the pressure range of 1 bar to 500 bar during (**a**) heating and (**b**) cooling. **c**, **d**, Isothermal entropy changes (ΔS_{it}), calculated by the quasi-direct method, for (**c**) decompression to ambient pressure and (**d**) compression from ambient pressure, which are obtained from heating and cooling data, respectively.

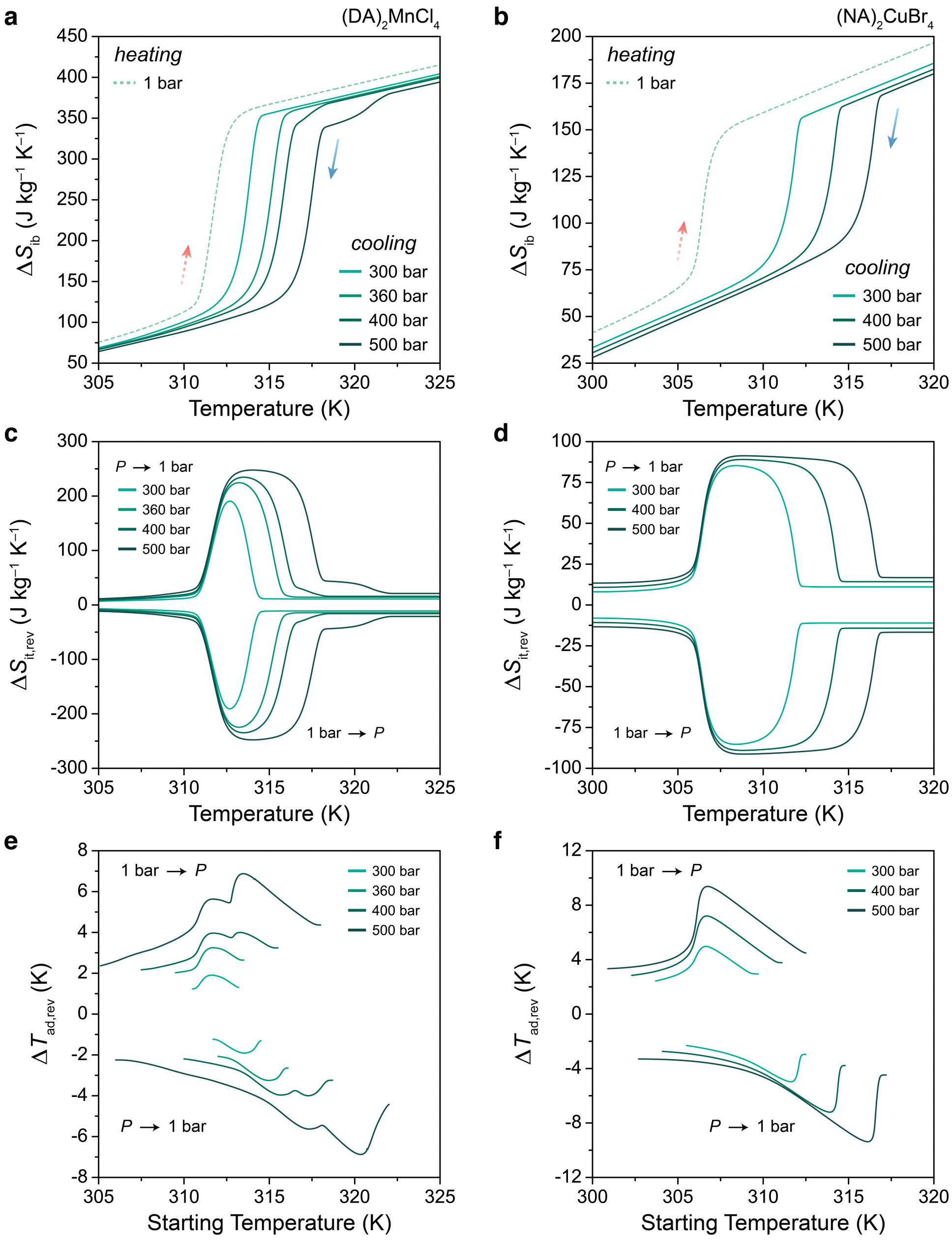

Supplementary Fig. 22 | Barocaloric effects at 300–500 bar. **a**, **b**, Isobaric entropy changes (ΔS_{ib}) during heating at ambient pressure (dashed line) and cooling at applied pressures of up to 500 bar (solid lines) for powder samples of (**a**) $(DA)_2MnCl_4$ and (**b**) $(NA)_2CuBr_4$. Note that the ΔS_{ib} curves include contributions from the heat capacity. **c**, **d**, Reversible isothermal entropy changes ($\Delta S_{it,rev}$) calculated from subtracting the $\Delta S_{ib}(T, P)_{cooling}$ from $\Delta S_{ib}(T, 1\text{ bar})_{heating}$ for (**c**) $(DA)_2MnCl_4$ and (**d**) $(NA)_2CuBr_4$. **e**, **f**, Reversible adiabatic temperature changes ($\Delta T_{ad,rev}$) are plotted as a function of temperature and pressure for (**e**) $(DA)_2MnCl_4$ and (**f**) $(NA)_2CuBr_4$. Note that starting temperatures were taken from 1-bar heating curves and high-pressure cooling curves for compression and decompression, respectively.

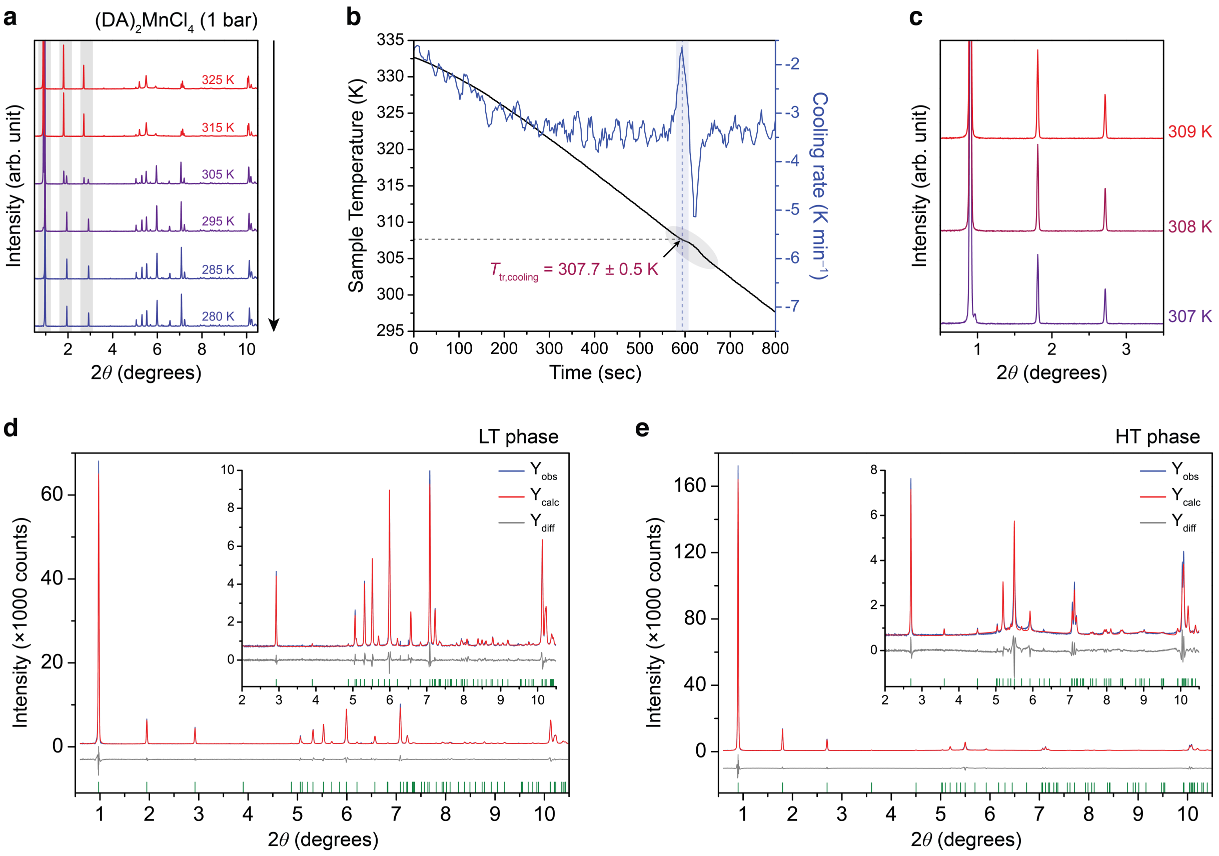

Supplementary Fig. 23 | Variable-temperature PXRD for $(DA)_2MnCl_4$ at 1 bar of He, while cooling from 335 K to 275 K, with an X-ray wavelength of 0.45237 Å. **a**, The PXRD patterns are shown at variable temperatures (as indicated). The red and blue patterns correspond to the high-temperature (HT) and low-temperature (LT) phases, respectively, with purple indicating patterns in which both phases are present during the transition from the HT to LT phase. **b**, A sample temperature trace recorded from a thermocouple embedded in the sample capillary is shown, along with the recorded cooling rate. Note that the thermocouple temperature trace features a peak, which is attributed to the latent heat of the exothermic transition. The transition temperature, T_{tr}, was determined using the first maximum of the cooling rate curve, and the uncertainty of the measurement was estimated as the full width at half maximum of the cooling rate peak. **c**, The phase transition from the HT to LT phase at T_{tr} was identified by monitoring the high-intensity (00*l*) peaks in the low-angle region, where the emergence of a higher-angle shoulder on the right side of each peak is associated with the emergence of the smaller unit cell of the LT phase. **d**, **e**, Le Bail refinement are shown for the (**d**) HT phase (330 K), R_{wp} = 7.25%, R_p = 5.16% and (**e**) LT phase (280 K), R_{wp} = 5.76%, R_p = 3.87%. Blue and red lines correspond to the observed and calculated diffraction patterns, respectively. The grey line represents the difference between observed and calculated patterns, and the green tick marks indicate calculated Bragg peak positions based on the refined unit cell. Unit cell parameters are listed in Supplementary Table 14.

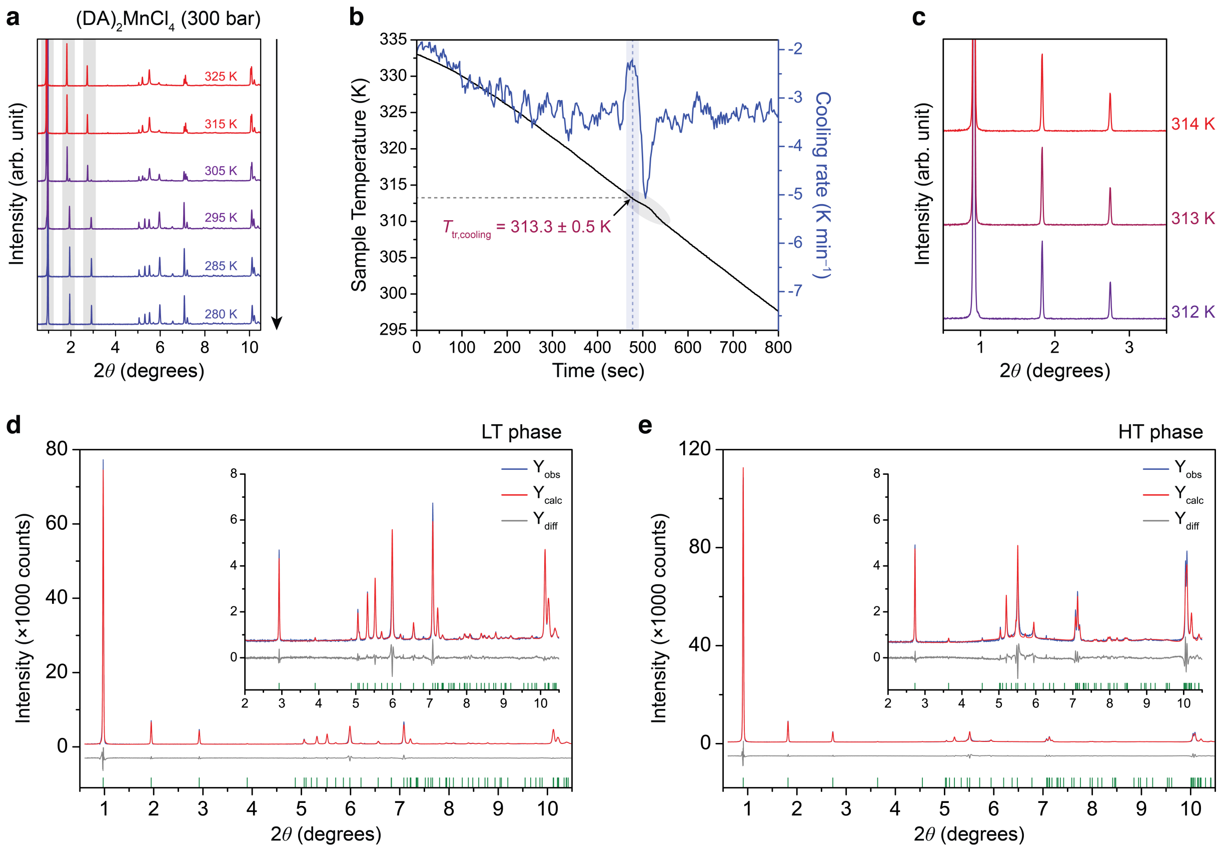

Supplementary Fig. 24 | Variable-temperature PXRD for $(DA)_2MnCl_4$ at 300 bar of He, while cooling from 335 K to 275 K, with an X-ray wavelength of 0.45237 Å. **a**, The PXRD patterns are shown at variable temperatures (as indicated). The red and blue patterns correspond to the HT and LT phases, respectively, with purple indicating patterns in which both phases are present during the transition from the HT to LT phase. **b**, A sample temperature trace recorded from a thermocouple embedded in the sample capillary is shown, along with the recorded cooling rate. Note that the thermocouple temperature trace features a peak, which is attributed to the latent heat of the exothermic transition. The transition temperature, T_{tr}, was determined using the first maximum of the cooling rate curve, and the uncertainty of the measurement was estimated as the full width at half maximum of the cooling rate peak. **c**, The phase transition from the HT to LT phase at T_{tr} was identified by monitoring the high-intensity (00*l*) peaks in the low-angle region, where the emergence of a higher-angle shoulder on the right side of each peak is associated with the emergence of the smaller unit cell of the LT phase. **d**, **e**, Le Bail refinement are shown for the (**d**) HT phase (330 K), R_{wp} = 6.75%, R_p = 4.84% and (**e**) LT phase (280 K), R_{wp} = 6.03%, R_p = 3.78%. Blue and red lines correspond to the observed and calculated diffraction patterns, respectively. The grey line represents the difference between observed and calculated patterns, and the green tick marks indicate calculated Bragg peak positions based on the refined unit cell. Unit cell parameters are listed in Supplementary Table 14.

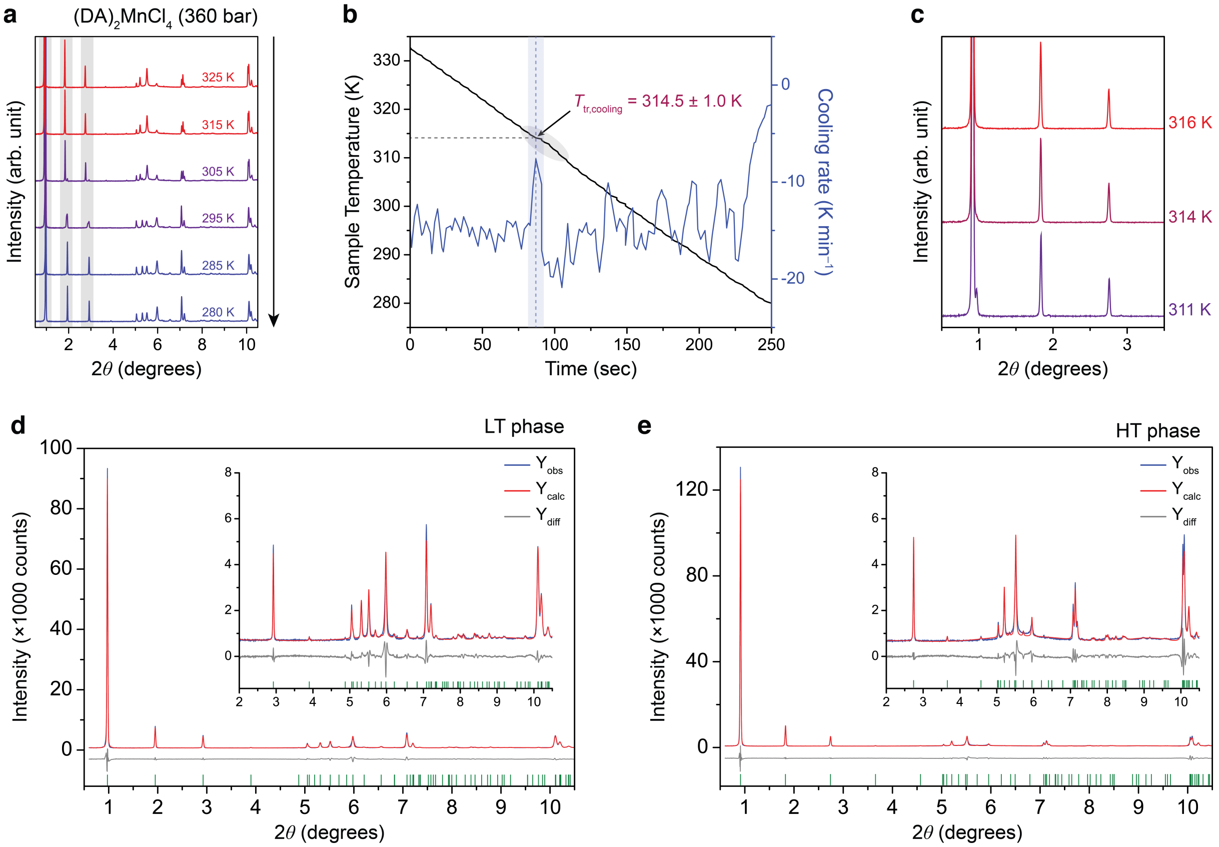

Supplementary Fig. 25 | Variable-temperature PXRD for $(DA)_2MnCl_4$ at 360 bar of He, while cooling from 335 K to 275 K, with an X-ray wavelength of 0.45237 Å. **a**, The PXRD patterns are shown at variable temperatures (as indicated). The red and blue patterns correspond to the high-temperature (HT) and low-temperature (LT) phases, respectively, with purple indicating patterns in which both phases are present during the transition from the HT to LT phase. **b**, A sample temperature trace recorded from a thermocouple embedded in the sample capillary is shown, along with the recorded cooling rate. Note that the thermocouple temperature trace features a peak, which is attributed to the latent heat of the exothermic transition. The transition temperature, T_{tr}, was determined using the first maximum of the cooling rate curve, and the uncertainty of the measurement was estimated as the full width at half maximum of the cooling rate peak. **c**, The phase transition from the HT to LT phase at T_{tr} was identified by monitoring the high-intensity (00*l*) peaks in the low-angle region, where the emergence of a higher-angle shoulder on the right side of each peak is associated with the emergence of the smaller unit cell of the LT phase. **d**, **e**, Le Bail refinements are shown for the (**d**) HT phase (330 K), R_{wp} = 7.30%, R_p = 5.23% and (**e**) LT phase (280 K), R_{wp} = 7.62%, R_p = 4.91%. Blue and red lines correspond to the observed and calculated diffraction patterns, respectively. The grey line represents the difference between observed and calculated patterns, and the green tick marks indicate calculated Bragg peak positions based on the refined unit cell. Unit cell parameters are listed in Supplementary Table 14.

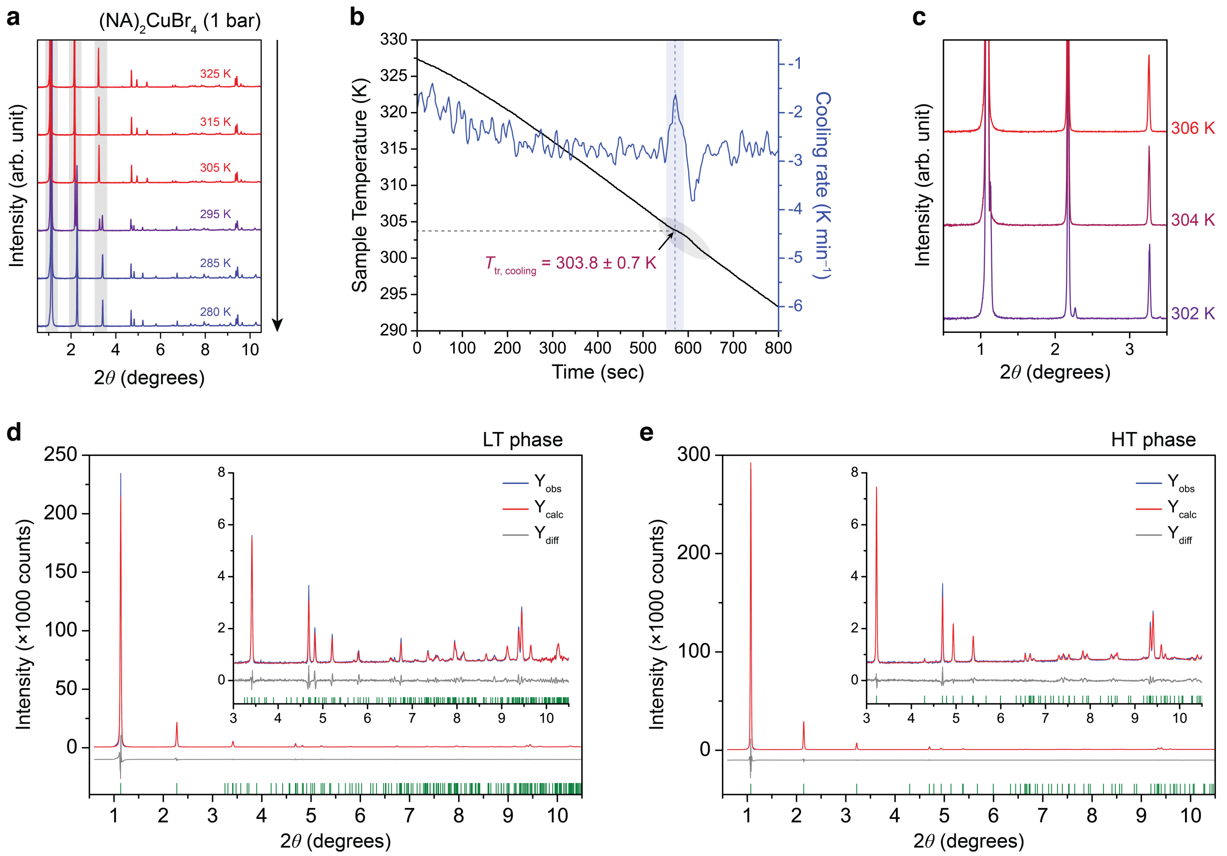

Supplementary Fig. 26 | Variable-temperature PXRD for $(NA)_2CuBr_4$ at 1 bar of He, while cooling from 335 K to 275 K, with an X-ray wavelength of 0.45237 Å. **a**, The PXRD patterns are shown at variable temperatures (as indicated). The red and blue patterns correspond to the HT and LT phases, respectively, with purple indicating patterns in which both phases are present during the transition from the HT to LT phase. **b**, A sample temperature trace recorded from a thermocouple embedded in the sample capillary is shown, along with the recorded cooling rate. Note that the thermocouple temperature trace features a peak, which is attributed to the latent heat of the exothermic transition. The transition temperature, T_{tr}, was determined using the first maximum of the cooling rate curve, and the uncertainty of the measurement was estimated as the full width at half maximum of the cooling rate peak. **c**, The phase transition from the HT to LT phase at T_{tr} was identified by monitoring the high-intensity (00*l*) peaks in the low-angle region, where the emergence of a higher-angle shoulder on the right side of each peak is associated with the emergence of the smaller unit cell of the LT phase. **d**, **e**, Le Bail refinements are shown for the (**d**) HT phase (330 K), R_{wp} = 6.70%, R_p = 4.29% and (**e**) LT phase (280 K), R_{wp} = 10.4%, R_p = 6.54%. Blue and red lines correspond to the observed and calculated diffraction patterns, respectively. The grey line represents the difference between observed and calculated patterns, and the green tick marks indicate calculated Bragg peak positions based on the refined unit cell. Unit cell parameters are listed in Supplementary Table 15.

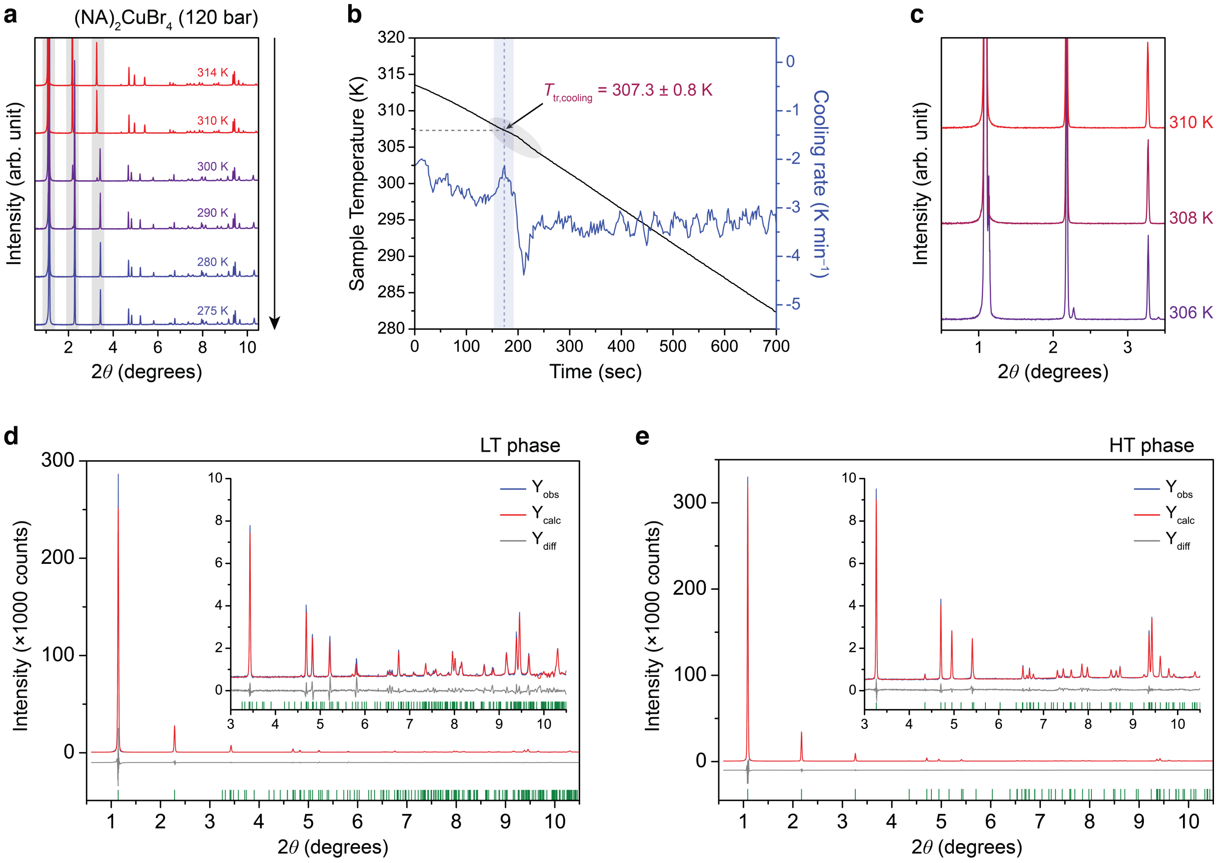

Supplementary Fig. 27 | Variable-temperature PXRD for $(NA)_2CuBr_4$ at 120 bar of He, while cooling from 315 K to 275 K, with an X-ray wavelength of 0.45237 Å. **a**, The PXRD patterns are shown at variable temperatures (as indicated). The red and blue patterns correspond to the HT and LT phases, respectively, with purple indicating patterns in which both phases are present during the transition from the HT to LT phase. **b**, A sample temperature trace recorded from a thermocouple embedded in the sample capillary is shown, along with the recorded cooling rate. Note that the thermocouple temperature trace features a peak, which is attributed to the latent heat of the exothermic transition. The transition temperature, T_{tr}, was determined using the first maximum of the cooling rate curve, and the uncertainty of the measurement was estimated as the full width at half maximum of the cooling rate peak. **c**, The phase transition from the HT to LT phase at T_{tr} was identified by monitoring the high-intensity (00*l*) peaks in the low-angle region, where the emergence of a higher-angle shoulder on the right side of each peak is associated with the emergence of the smaller unit cell of the LT phase. **d**, **e**, Le Bail refinements are shown for the (**d**) HT phase (314 K), R_{wp} = 6.64%, R_p = 4.58% and (**e**) LT phase (280 K), R_{wp} = 9.81%, R_p = 7.16%. Blue and red lines correspond to the observed and calculated diffraction patterns, respectively. The grey line represents the difference between observed and calculated patterns, and the green tick marks indicate calculated Bragg peak positions based on the refined unit cell. Unit cell parameters are listed in Supplementary Table 15.

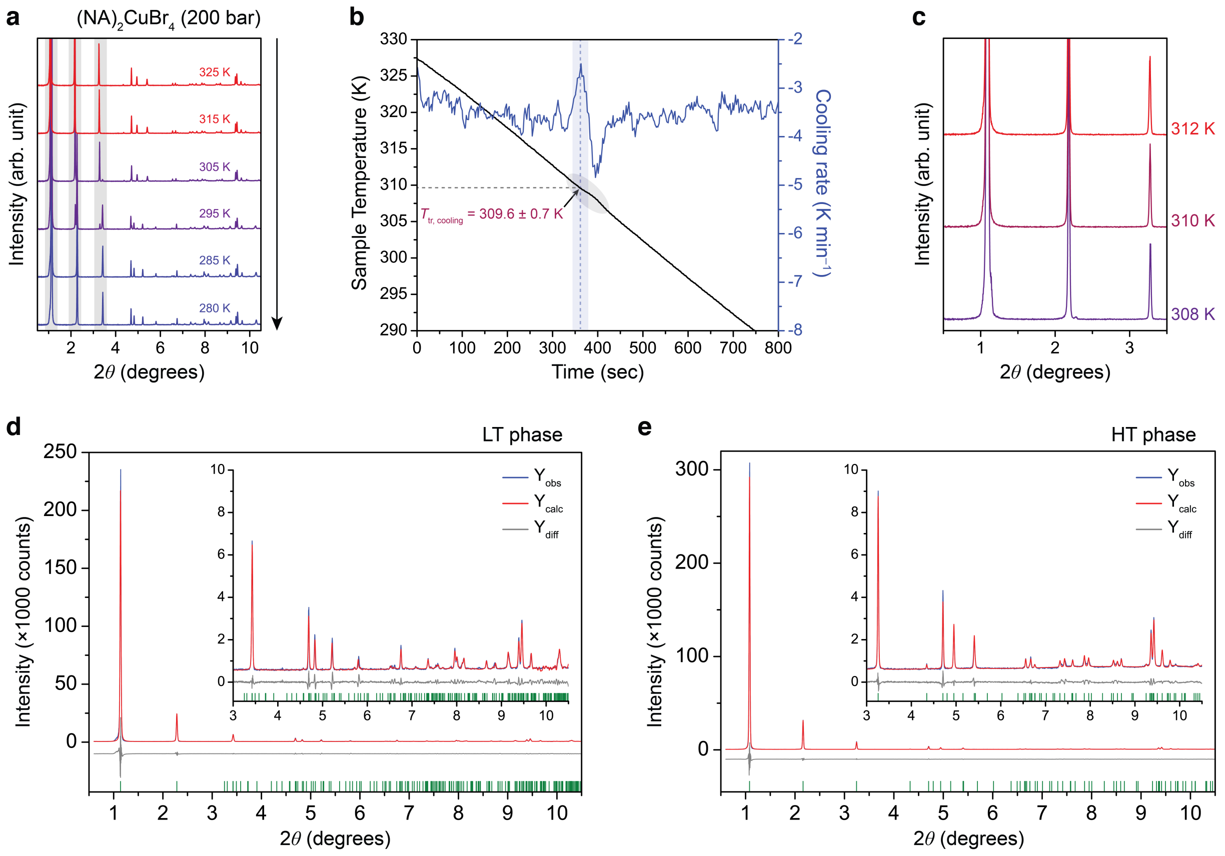

Supplementary Fig. 28 | Variable-temperature PXRD for $(NA)_2CuBr_4$ at 200 bar of He, while cooling from 335 K to 275 K, with an X-ray wavelength of 0.45237 Å. **a**, The PXRD patterns are shown at variable temperatures (as indicated). The red and blue patterns correspond to the HT and LT phases, respectively, with purple indicating patterns in which both phases are present during the transition from the HT to LT phase. **b**, A sample temperature trace recorded from a thermocouple embedded in the sample capillary is shown, along with the recorded cooling rate. Note that the thermocouple temperature trace features a peak, which is attributed to the latent heat of the exothermic transition. The transition temperature, T_{tr}, was determined using the first maximum of the cooling rate curve, and the uncertainty of the measurement was estimated as the full width at half maximum of the cooling rate peak. **c**, The phase transition from the HT to LT phase at T_{tr} was identified by monitoring the high-intensity (00*l*) peaks in the low-angle region, where the emergence of a higher-angle shoulder on the right side of each peak is associated with the emergence of the smaller unit cell of the LT phase. **d**, **e**, Le Bail refinements are shown for the (**d**) HT phase (314 K), R_{wp} = 7.03%, R_p = 4.82% and (**e**) LT phase (280 K), R_{wp} = 12.3%, R_p = 7.98%. Blue and red lines correspond to the observed and calculated diffraction patterns, respectively. The grey line represents the difference between observed and calculated patterns, and the green tick marks indicate calculated Bragg peak positions based on the refined unit cell. Unit cell parameters are listed in Supplementary Table 15.

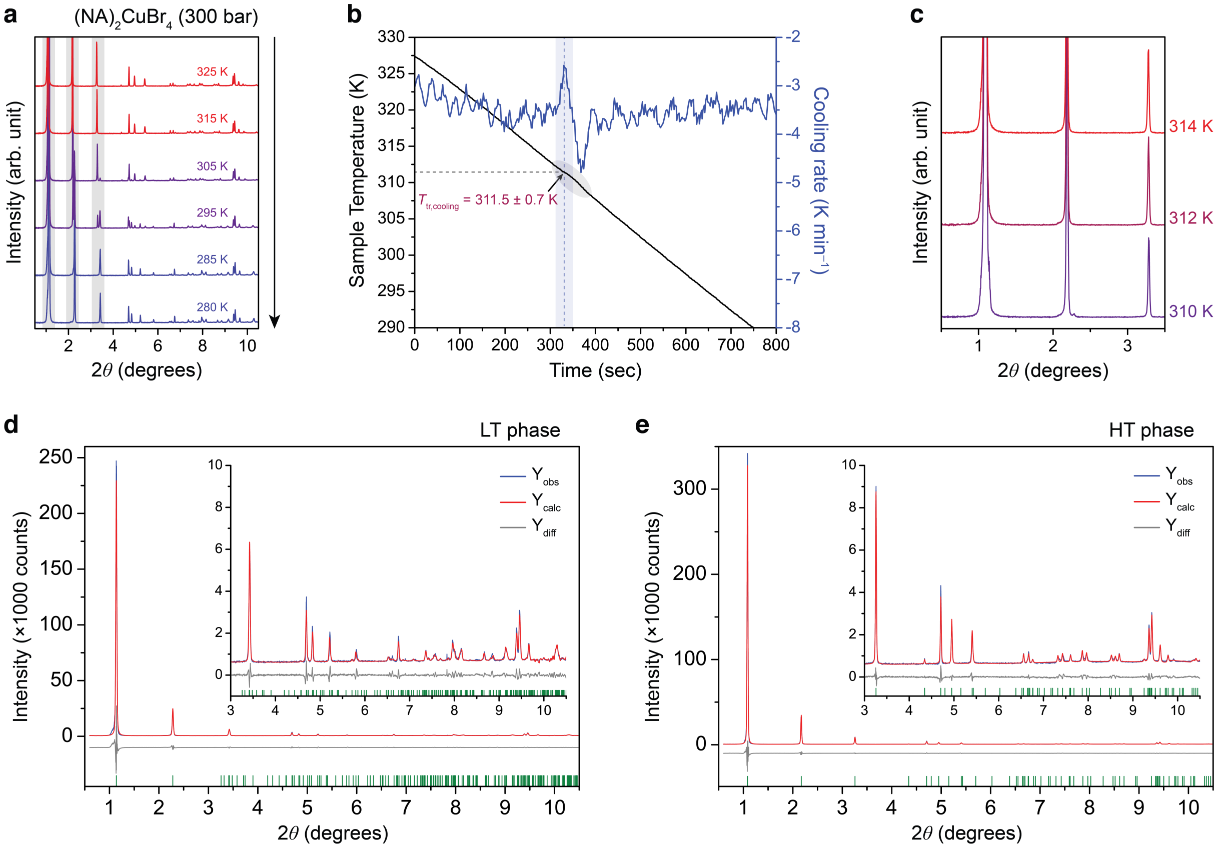

Supplementary Fig. 29 | Variable-temperature PXRD for $(NA)_2CuBr_4$ at 300 bar of He, while cooling from 335 K to 275 K, with an X-ray wavelength of 0.45237 Å. **a**, The PXRD patterns are shown at variable temperatures (as indicated). The red and blue patterns correspond to the HT and LT phases, respectively, with purple indicating patterns in which both phases are present during the transition from the HT to LT phase. **b**, A sample temperature trace recorded from a thermocouple embedded in the sample capillary is shown, along with the recorded cooling rate. Note that the thermocouple temperature trace features a peak, which is attributed to the latent heat of the exothermic transition. The transition temperature, T_{tr}, was determined using the first maximum of the cooling rate curve, and the uncertainty of the measurement was estimated as the full width at half maximum of the cooling rate peak. **c**, The phase transition from the HT to LT phase at T_{tr} was identified by monitoring the high-intensity (00*l*) peaks in the low-angle region, where the emergence of a higher-angle shoulder on the right side of each peak is associated with the emergence of the smaller unit cell of the LT phase. **d**, **e**, Le Bail refinements are shown for the (**d**) HT phase (314 K), R_{wp} = 8.33%, R_p = 5.38% and (**e**) LT phase (280 K), R_{wp} = 14.7%, R_p = 9.29%. Blue and red lines correspond to the observed and calculated diffraction patterns, respectively. The grey line represents the difference between observed and calculated patterns, and the green tick marks indicate calculated Bragg peak positions based on the refined unit cell. Unit cell parameters are listed in Supplementary Table 15.

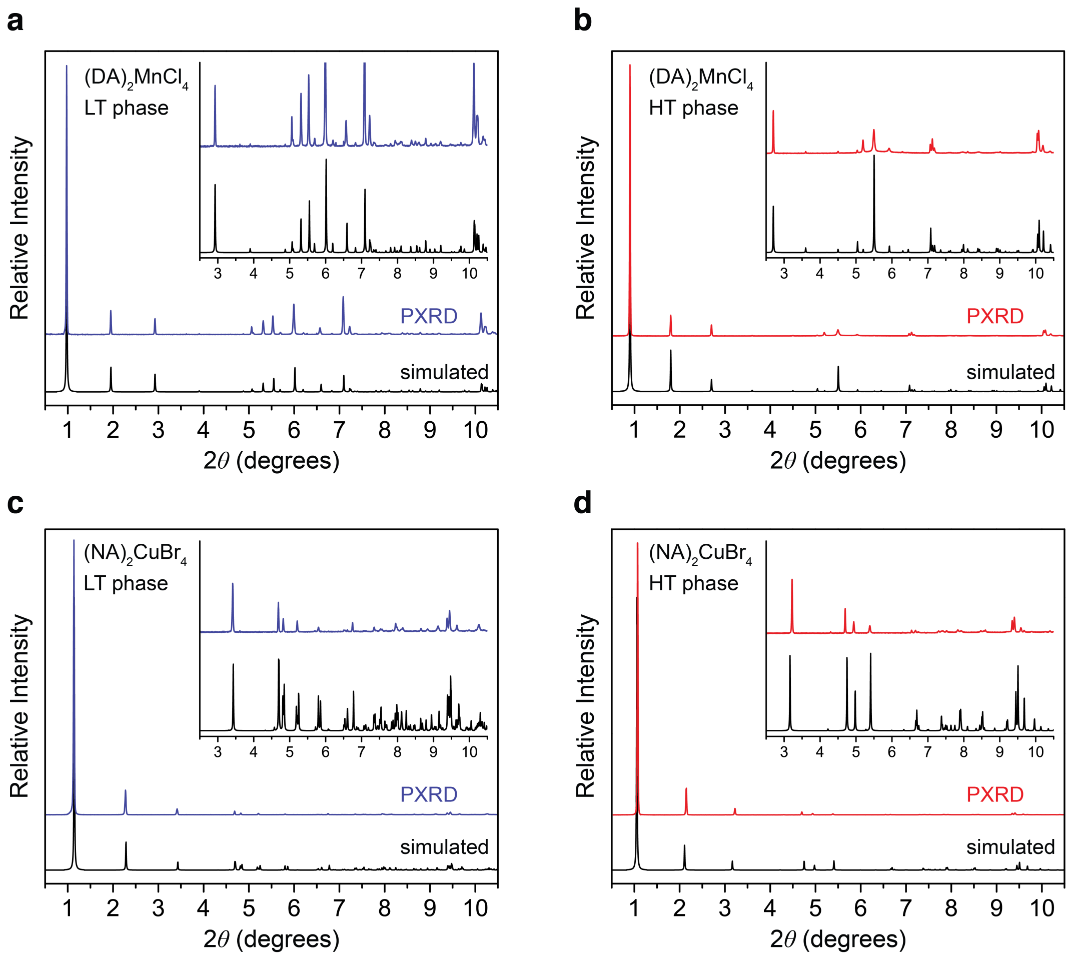

Supplementary Fig. 30 | Comparison of PXRD patterns to the calculated diffraction patterns from single crystal structures for (**a**–**b**) $(DA)_2MnCl_4$ and (**c**–**d**) $(NA)_2CuBr_4$ at ambient pressure. Note that the powder patterns were taken at 280 K and 330 K for the LT and HT phases, respectively. The LT phase crystal structures were determined at 270 K for both compounds, while the HT phase structures were determined at 330 K for $(DA)_2MnCl_4$ and 335 K for $(NA)_2CuBr_4$.

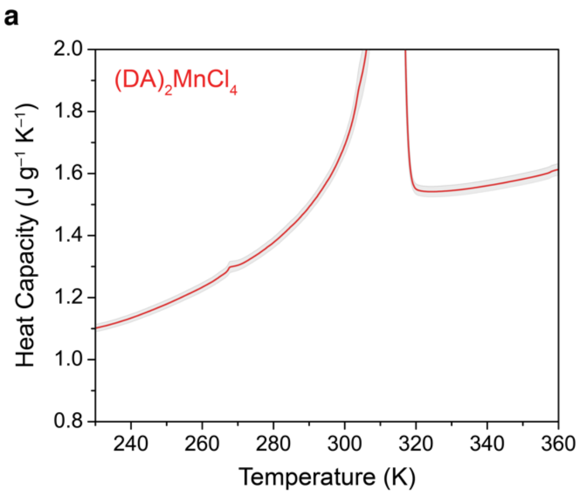

Supplementary Fig. 31 | a, **b**, Temperature dependence of the specific heat capacity, c_p, for (**a**) $(DA)_2MnCl_4$ and (**b**) $(NA)_2CuBr_4$ obtained while heating with a scan rate of 10 K min^{-1}. Errors, which were calculated as 2.8 times the standard deviation, are indicated by the grey shaded area. The heat capacity curves were calculated from 8 separate heat capacity datasets for each compound.

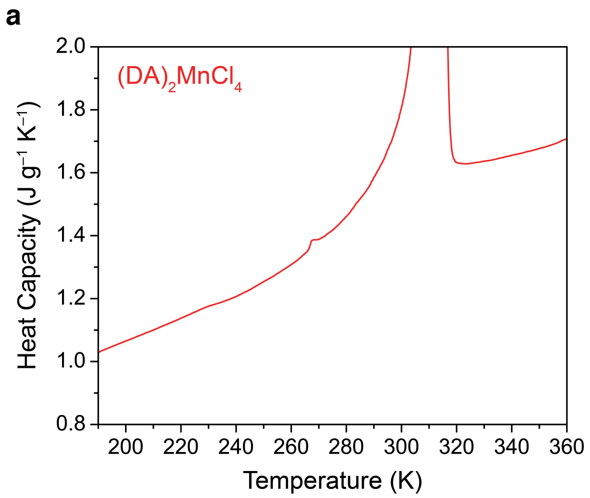

Supplementary Fig. 32 | a, **b**, Temperature dependence of the specific heat capacity for (**a**) $(DA)_2MnCl_4$ and (**b**) $(NA)_2CuBr_4$ obtained from heating with a scan rate of 10 K min^{-1}. The data shown represents one three-run ASTM heat capacity set, with no averaging. Unlike $(DA)_2MnCl_4$, $(NA)_2CuBr_4$ displays a broad feature from 220–250 K that is consistent with a gradual activation of conformational degrees of freedom prior to the hydrocarbon order-disorder transition.

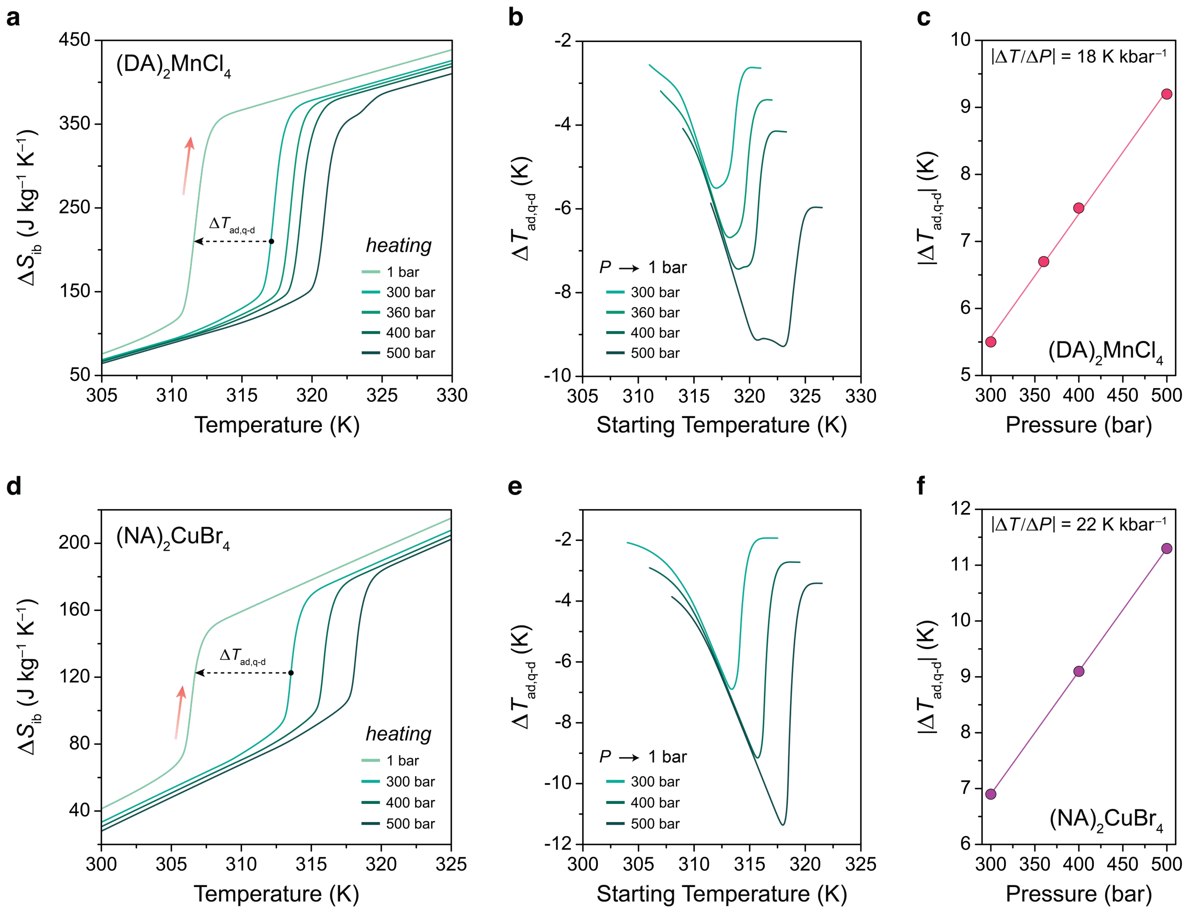

Supplementary Fig. 33 | Calculations of adiabatic temperature changes via the quasi-direct method for (**a-c**) $(DA)_2MnCl_4$ and (**d-f**) $(NA)_2CuBr_4$. To calculate decompression-induced temperature changes, $\Delta T_{ad,q\text{-}d}$, (shown in **b** and **e**), the temperature difference at a horizontal (adiabatic) line—defined between isobaric entropy curves at ambient pressure and an applied pressure during heating (shown in **a** and **d**)—is plotted as a function of starting temperature from the high-pressure curves. Note that the $\Delta T_{ad,q\text{-}d}$ values shown here correspond to the irreversible temperature decrease upon first decompression. (**e, f**) The peak values for $\Delta T_{ad,q\text{-}d}$ curves are plotted as a function of operating pressure. The dependence of irreversible $\Delta T_{ad,q\text{-}d}$ values to operating pressure, $\Delta T/\Delta P$, coincides with the pressure sensitivity of the materials as expected.

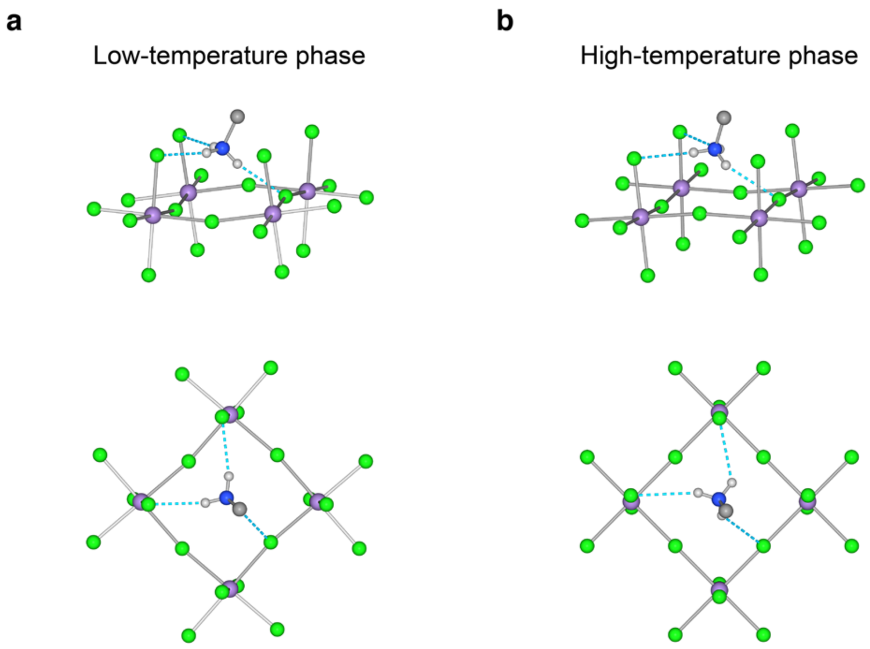

Supplementary Fig. 34 | a, **b**, Hydrogen bonding interactions (N–H···Cl) of decylammonium (DA) chains within the Mn–Cl pocket of the (**a**) LT and (**b**) HT phases of $(DA)_2MnCl_4$. Purple, green, grey, blue, and white spheres represent Mn, Cl, C, N, and H atoms, respectively. Only N1 and C1 atoms are shown for the DA chains, and H atoms on C atoms are omitted for clarity. Donor–acceptor (N···Cl) distances and bond angles for the hydrogen bonds are summarized in Supplementary Table 20.

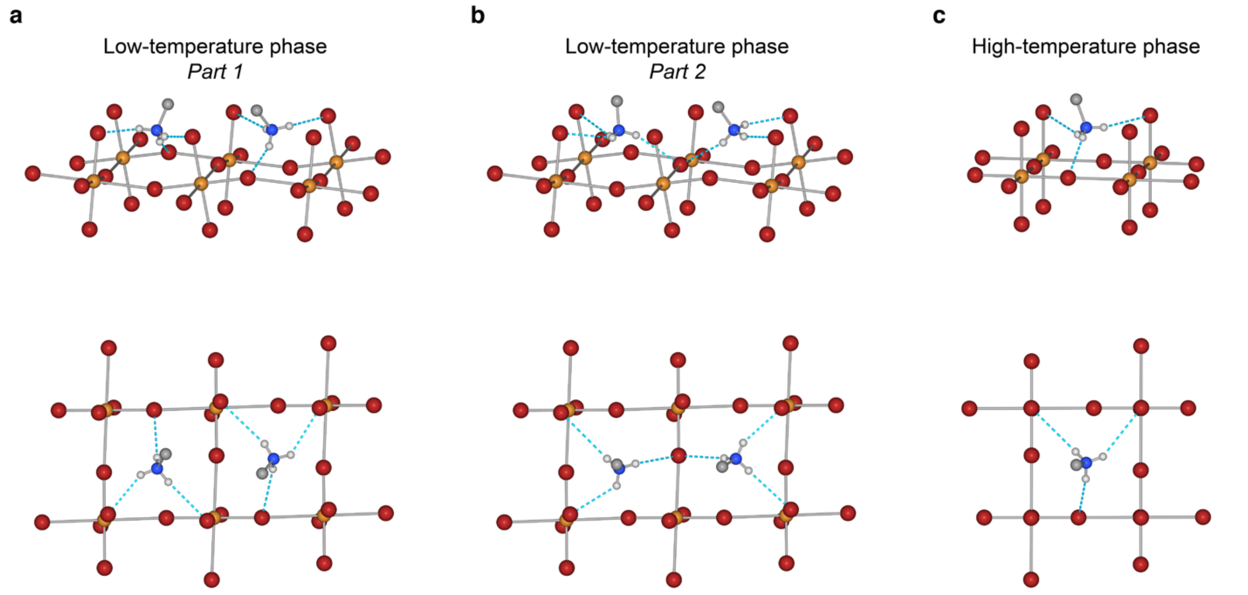

Supplementary Fig. 35 | a–c, Hydrogen bonding interactions (N–H···Br) of nonylammonium (NA) chains within the Cu–Br pocket of the (**a** and **b**) LT and (**c**) HT phases of $(NA)_2CuBr_4$. Orange, brown, grey, blue, and white spheres represent Cu, Br, C, N, and H atoms, respectively. NA chains in the LT phase display two conformations: chain B (C2–C3 gauche bond), which is shown on the left, and chain A (C1–C2 gauche bond), which is shown on the right. Note that each chain in the LT phase is modeled with two-part disorder. Only N1 and C1 atoms are shown for the NA chains, and H atoms on C atoms are omitted for clarity. Donor–acceptor (N···Br) distances and bond angles for the hydrogen bonds are summarized in Supplementary Table 20.

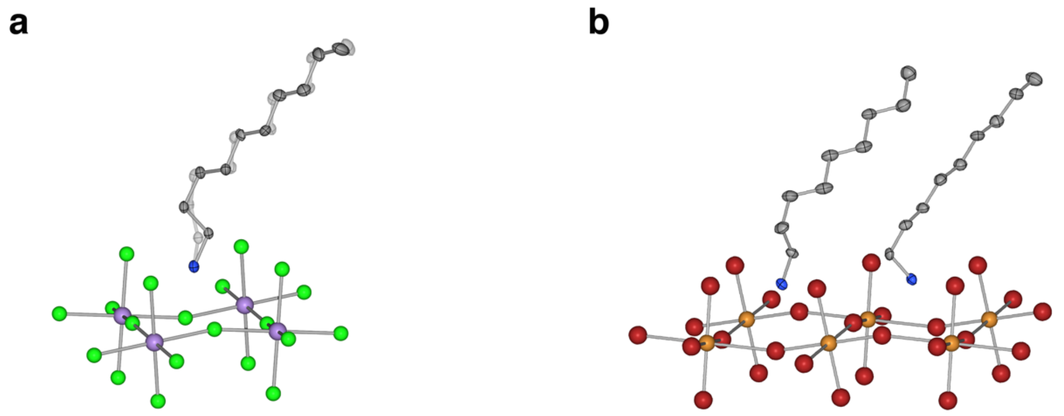

Supplementary Fig. 36 | a, **b**, Conformations of the alkylammonium chains for (**a**) $(DA)_2MnCl_4$ and (**b**) $(NA)_2CuBr_4$ at 100 K, with atomic displacement parameters shown at 50% probability for the C and N atoms of the alkylammonium chains. Purple, orange, green, brown, grey, and blue spheres represent Mn, Cu, Cl, Br, C, and N atoms, respectively. H atoms are omitted for clarity. Note that DA chains are modeled with two-part disorder.

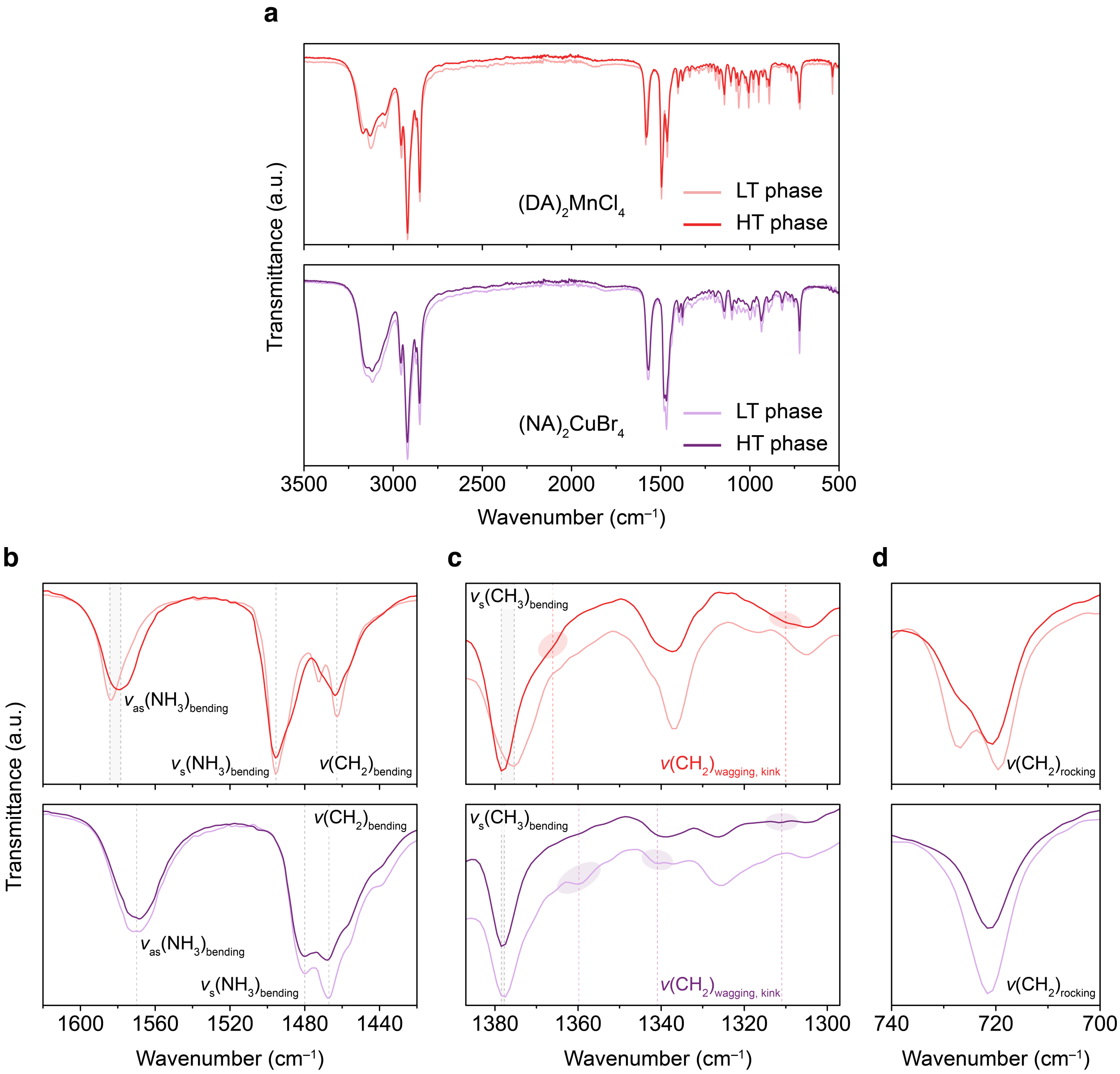

Supplementary Fig. 37 | Variable-temperature infrared spectra**. a**, The low-temperature (LT) and high-temperature (HT) phase infrared spectra were collected 5 K below and 5 K above the phase transition temperature, respectively, for $(DA)_2MnCl_4$ (top) and $(NA)_2CuBr_4$ (bottom). **b**–**d**, Zoomed in views of three different regions with bands that correspond to (**b**) NH_3 bending and CH_2 bending, (**c**) CH_3 bending and CH_2 wagging, and (**d**) CH_2 rocking bands. Key shifts in peak positions are indicated by vertical grey bars, and CH_2 wagging bands associated with conformational defects are highlighted with red and purple dashed lines for $(DA)_2MnCl_4$ and $(NA)_2CuBr_4$, respectively. The IR bands used for conformational analysis are summarized in Supplementary Table 13.

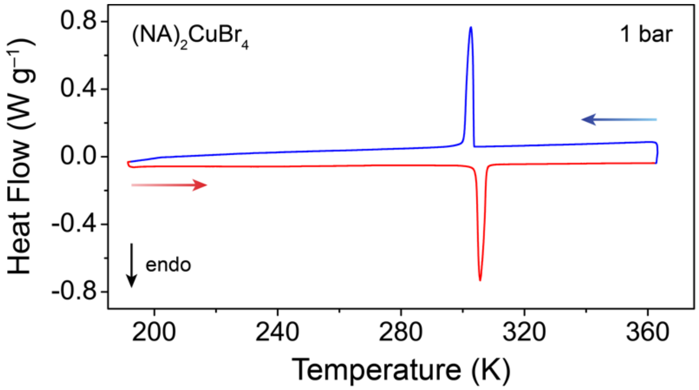

Supplementary Fig. 38 | DSC trace at ambient pressure for $(NA)_2CuBr_4$ from 190 to 360 K with heating and cooling rates of 4 K min^{-1}. Note that no heat flow signals indicative of a minor phase transition are observed over this temperature range.

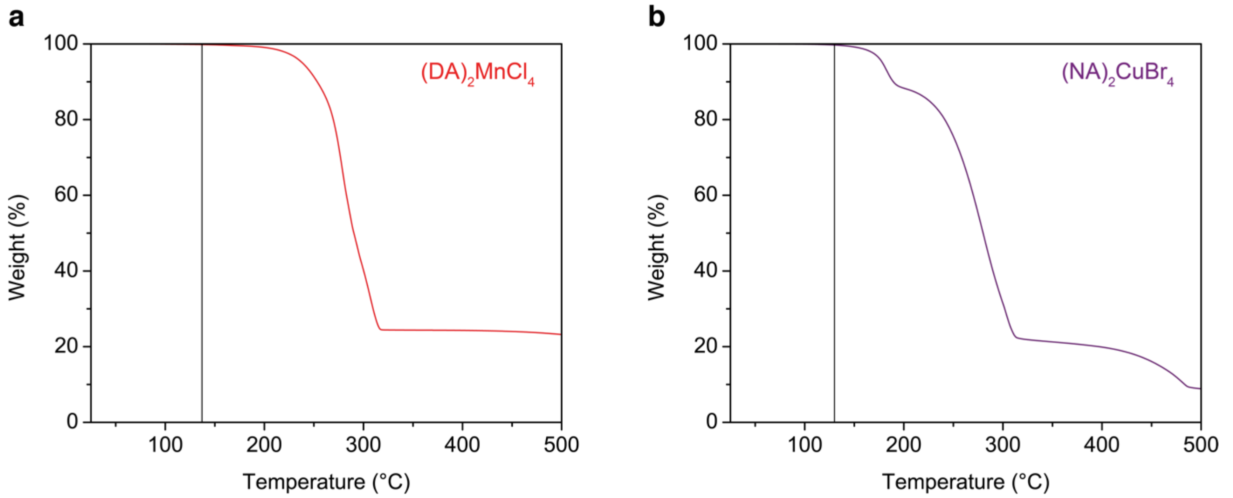

Supplementary Fig. 39 | a, **b**, Thermogravimetric analysis of (**a**) $(DA)_2MnCl_4$ and (**b**) $(NA)_2CuBr_4$ at a heating rate of 4 K min^{-1}. A decrease of less than 0.4 wt % was observed from ambient temperature to 100 °C above the transition temperature (vertical line).

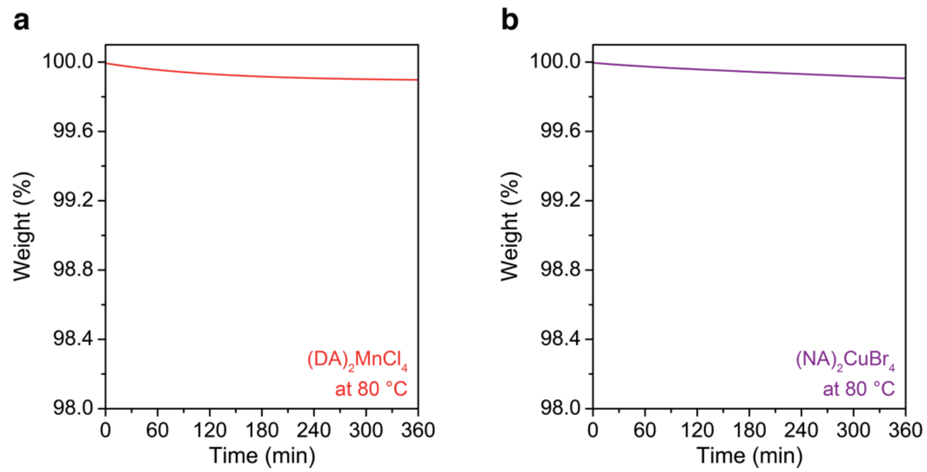

Supplementary Fig. 40 | a, **b**, Thermal stability at 80 °C evaluated by TGA under flowing N_2 for (**a**) $(DA)_2MnCl_4$ and (**b**) $(NA)_2CuBr_4$. Both compounds remain thermally stable with negligible weight change (< 0.1 %).

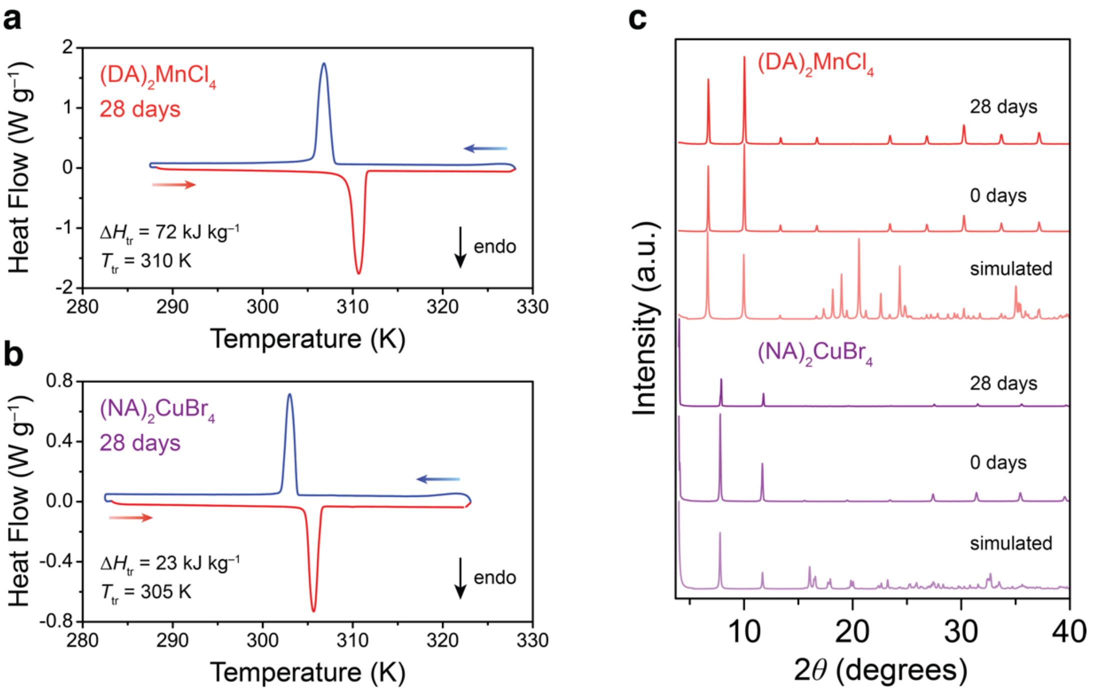

Supplementary Fig. 41 | Chemical stability of $(DA)_2MnCl_4$ and $(NA)_2CuBr_4$ evaluated by performing DSC and PXRD experiments after 28 days under ambient conditions (50% relative humidity).

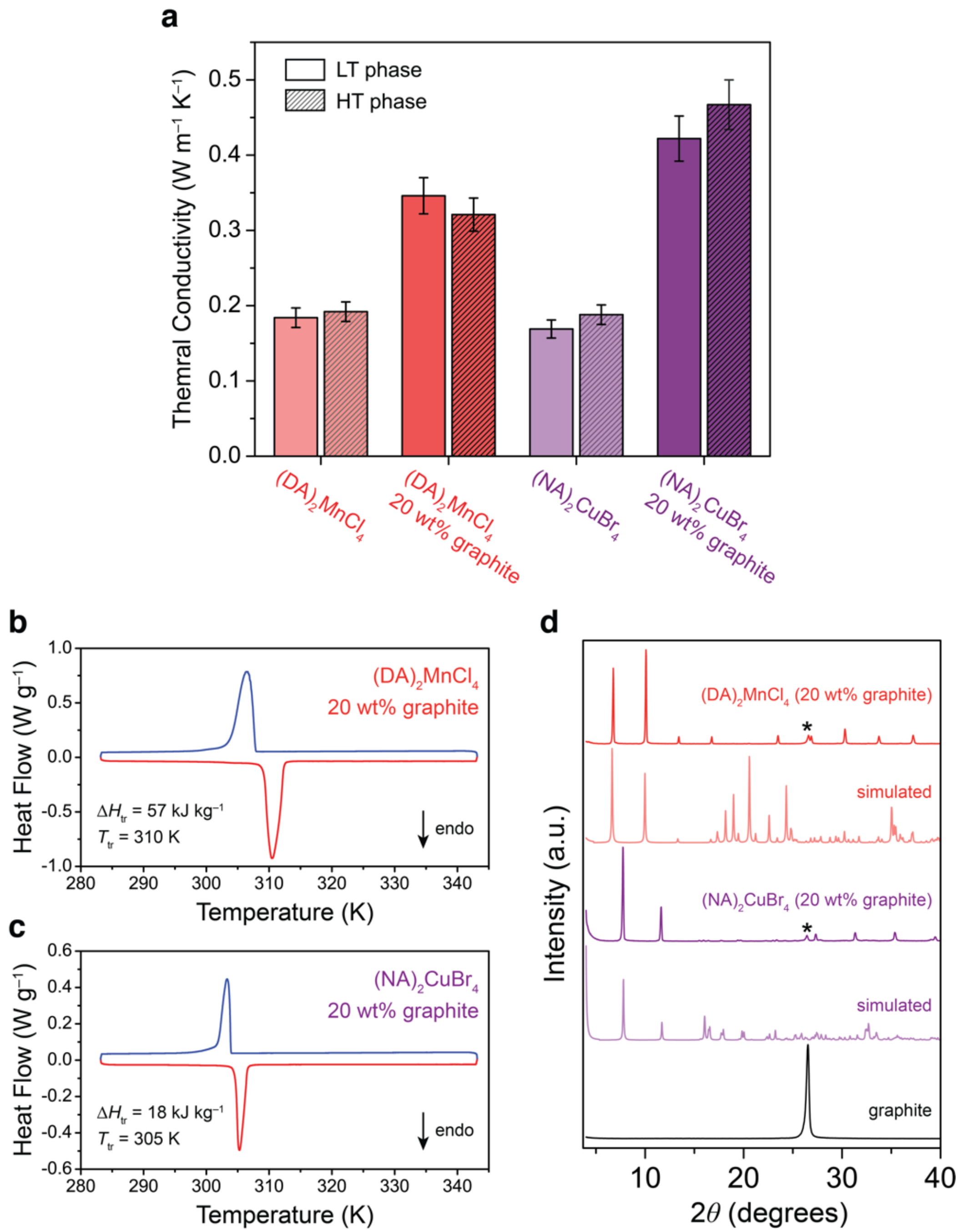

Supplementary Fig. 42 | Thermal conductivity measurements. **a,** Thermal conductivities of pressed pellets of $(DA)_2MnCl_4$ and $(NA)_2CuBr_4$ with and without 20 wt % graphite were measured through thermal diffusivity measurements using the laser flash analysis technique. The measurements were carried out at 10 °C below and 15 °C above the transition temperature for the LT and HT phases, respectively, of $(DA)_2MnCl_4$, and at 10 °C below and above the transition temperature for the LT and HT phases, respectively, of $(NA)_2CuBr_4$. **b**, **c**, DSC traces for (**b**) $(DA)_2MnCl_4$ and (**c**) $(NA)_2CuBr_4$ mixed with 20 wt % graphite. **d**, PXRD patterns of the perovskite samples mixed with 20 wt % graphite. Asterisks denote diffraction peaks from graphite.

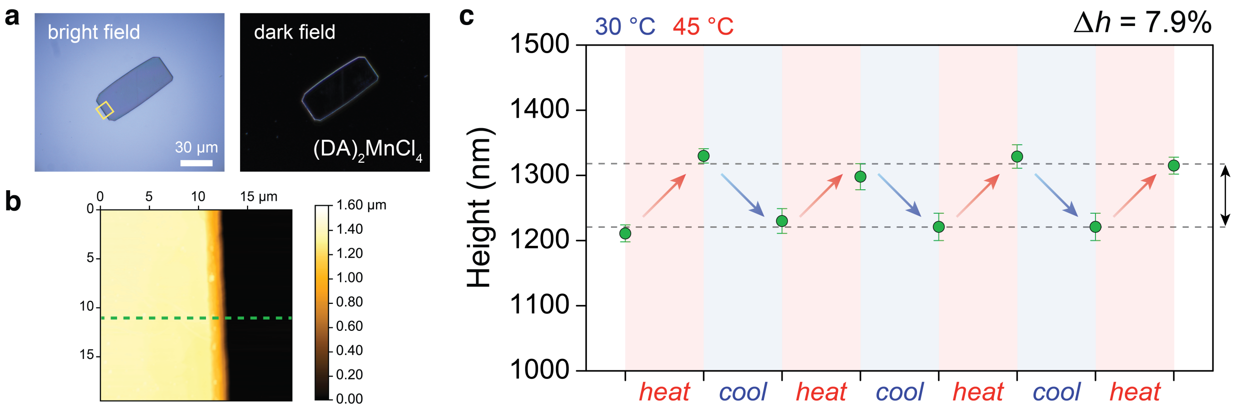

Supplementary Fig. 43 | Thermal cycling of a micron-thick single crystal of $(DA)_2MnCl_4$ between 30 °C and 45 °C, with (**a**) optical images taken at room temperature, (**b**) an AFM image taken at 45 °C, and (**c**) thickness as a function of temperature. The thickness was measured for the area specified by the yellow box in **a** through a line profile (green dashed line) in **b**. The average thickness at each phase is marked using the dashed line. Isobaric HP-DSC experiments for single crystals on a Si substrate are shown in Supplementary Fig. 44.

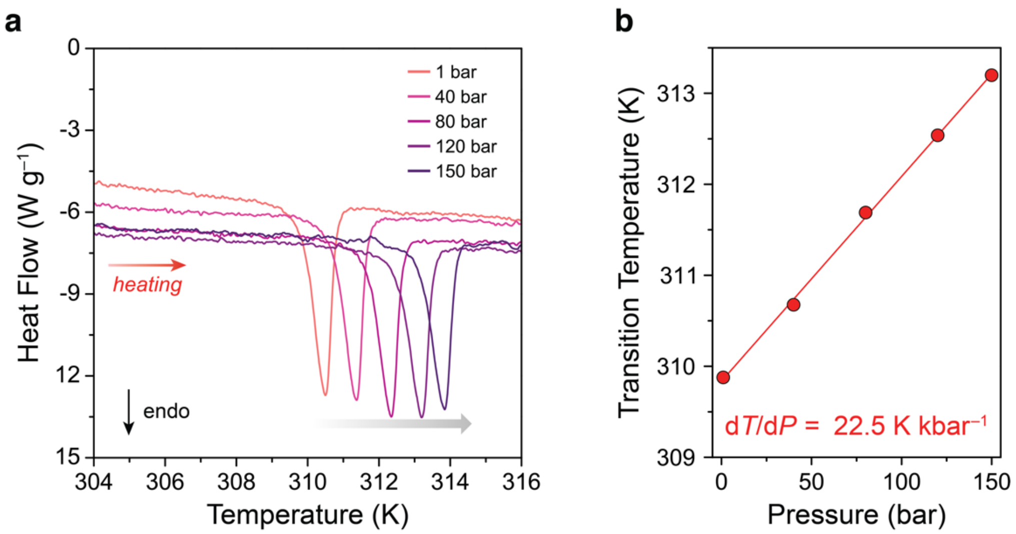

Supplementary Fig. 44 | a, DSC measurements under applied hydrostatic pressure for single crystals of $(DA)_2MnCl_4$ directly grown on a Si wafer during heating, with scan rates of 4 K min^{-1}. Due to the small sample mass (of ~0.04 mg) and noisy baseline, the peaks were not integrated. **b**, Pressure dependence of transition temperatures d*T*/d*P* obtained from the isobaric HP-DSC experiment.

Supplementary Table 1. Summary of the thermodynamics of order-disorder transitions in two-dimensional $(C_nH_{2n+1}NH_3)_2MnCl_4$ perovskites used in Fig. 1b.

Chemical Formula[a]	Type[b]	T_{tr}[c] (K)	ΔH (kJ mol^{-1})	ΔH (kJ kg^{-1})	ΔS (J mol^{-1} K^{-1})	ΔS (J kg^{-1} K^{-1})	Ref.
$(C_6)_2MnCl_4$	*minor*	206	5	13	26	64	[32]
	major	291	10	25	37	93	
	total		15	38	63	157	
$(C_7)_2MnCl_4$	*major*	250	17	39	68	159	[33]
	minor	314	10	24	33	76	
	total		27	63	101	235	
$(C_9)_2MnCl_4$[d]	*major*	291	26	53	89	183	[34]
	minor	294	2	5	8	16	
	total		28	58	97	199	
$(C_{10})_2MnCl_4$		308	35	68	113	221	[34]
		310	37	71	118	230	*this work*
$(C_{11})_2MnCl_4$	*major*	317	40	74	126	234	[34]
	minor	321	4	8	13	25	
	total		44	82	140	258	
$(C_{12})_2MnCl_4$	*major*	331	48	84	145	254	[35]
	minor	335	6	10	18	31	
	total		54	94	162	285	
$(C_{13})_2MnCl_4$	*major*	331	52	87	158	264	[34]
	minor	343	8	13	22	37	
	total		60	100	180	301	
$(C_{14})_2MnCl_4$	*major*	345	58	92	167	268	[35]
	minor	357	9	15	26	41	
	total		67	107	193	309	
$(C_{15})_2MnCl_4$	*major*	340	63	96	184	282	[34]
	minor	362	10	16	28	44	
	total		73	112	213	325	
$(C_{16})_2MnCl_4$	*major*	346	60	88	172	253	[34]
	minor	364	12	17	32	46	
	total		71	104	204	299	

[a]$C_n = C_nH_{2n+1}NH_3$. [b]When a compound displays multiple transitions, the transition with the highest ΔS was labeled as a major transition. [c]Measured during the first heating scan. [d]Closely spaced transitions were not resolved in the original report, with only one transition at 287 K. Here, we synthesized this compound according to a reported procedure[34,36], and characterized the compound by DSC at a slow scan rate (0.5 K min^{-1}) to resolve the major and minor transitions. [e]The difference between the previously reported values and those reported in this work are within experimental uncertainties; however, the higher T_{tr} reported here may be the result of higher sample purity.

Supplementary Table 2. Summary of the thermodynamics of order-disorder transitions in two-dimensional $(C_nH_{2n+1}NH_3)_2CuX_4$ perovskites (X = Cl, Br) used in Fig. 1b.

Chemical Formula[a]	Type[b]	T_{tr}[c] (K)	ΔH (kJ mol⁻¹)	ΔH (kJ kg⁻¹)	ΔS (J mol⁻¹ K⁻¹)	ΔS (J kg⁻¹ K⁻¹)	Ref.
$(C_8)_2CuCl_4$	*major*	269	18	39	68	146	[25]
	minor	303	5	11	16	35	
	total		23	50	84	181	
$(C_9)_2CuCl_4$	*major*	294	23	46	78	158	[25]
	minor	303	5	9	15	31	
	total		28	56	93	189	
$(C_{10})_2CuCl_4$	*major*	309	35	67	113	216	[25]
	minor	312	5	9	15	28	
	total		39	75	128	244	
$(C_{12})_2CuCl_4$	*major*	328	40	69	121	210	[25]
	minor	334	8	14	25	43	
	total		48	83	146	253	
$(C_{13})_2CuCl_4$	*major*	333	48	80	145	239	[34]
	minor	344	10	16	29	48	
	total		58	96	174	287	
$(C_{14})_2CuCl_4$	*major*	334	50	61	150	237	[35]
	minor	356	11	83	31	48	
	total		61	144	181	285	
$(C_{15})_2CuCl_4$	*major*	343	54	82	158	238	[34]
	minor	358	12	18	33	50	
	total		66	100	191	288	
$(C_{16})_2CuCl_4$	*major*	345	36	52	103	150	[34]
	minor	354	8	12	23	33	
	minor	360	14	20	39	56	
	total		58	83	165	239	
$(C_9)_2CuBr_4$		305	15	23	51	76	*this work*
$(C_{11})_2CuBr_4$	*major*	328	19	26	59	80	[29]
	minor	340	1	1	1	2	
	total		20	27	60	82	
$(C_{12})_2CuBr_4$		337	22	29	64	85	
$(C_{13})_2CuBr_4$		344	30	38	86	109	
$(C_{14})_2CuBr_4$		348	28	34	80	98	
$(C_{15})_2CuBr_4$		354	39	46	110	131	
$(C_{16})_2CuBr_4$	*major*	343	2	2	4	5	
	minor	357	37	43	105	120	
	total		39	45	109	125	

[a]$C_n = C_nH_{2n+1}NH_3$. [b]When a compound displays multiple transitions, the transition with the highest ΔS was labeled as a major transition. [c]Measured during the first heating scan.

Supplementary Table 3. Summary of the thermodynamics of order-disorder transitions in two-dimensional $(C_nH_{2n+1}NH_3)_2CdCl_4$ and $(C_nH_{2n+1}NH_3)_2PbI_4$ perovskites used in Fig. 1b.

Chemical Formula[a]	Type[b]	T_{tr}[c] (K)	ΔH (kJ mol^{-1})	ΔH (kJ kg^{-1})	ΔS (J mol^{-1} K^{-1})	ΔS (J kg^{-1} K^{-1})	Ref.
$(C_7)_2CdCl_4$	*major*	250	18	36	71	147	[33]
	minor	317	5	10	16	33	
	total		23	47	87	180	
$(C_8)_2CdCl_4$	*major*	269	15	28	54	105	[37]
	minor	308	5	10	17	32	
	total		20	38	71	138	
$(C_{10})_2CdCl_4$	*minor*	308	8	14	25	44	[5]
	major	313	30	52	95	166	
	total		38	66	120	210	
$(C_{12})_2CdCl_4$	*minor*	332	11	17	33	52	[35]
	major	334	44	69	130	208	
	total		54	87	163	260	
$(C_{14})_2CdCl_4$	*major*	345	40	58	115	168	[35]
	minor	351	23	34	66	96	
	total		63	92	181	264	
$(C_{16})_2CdCl_4$	*major*	345	40	55	117	158	[38]
	minor	352	8	10	22	30	
	minor	356	32	43	88	120	
	total		80	108	227	308	
$(C_7)_2PbI_4$	*minor*	271	7	7	25	26	[39]
	major	286	8	9	29	31	
	minor	310	3	3	9	10	
	total		18	19	63	67	
$(C_8)_2PbI_4$	*minor*	252	15	15	58	59	[39]
	major	311	21	22	68	70	
	total		36	37	126	129	
$(C_9)_2PbI_4$	*minor*	252	7	7	28	28	[39]
	major	314	24	24	75	75	
	total		31	31	104	103	
$(C_{10})_2PbI_4$	*minor*	259	10	10	39	38	[39]
	minor	284	8	8	30	29	
	major	337	32	31	96	93	
	total		51	49	165	160	
$(C_{12})_2PbI_4$	*minor*	315	11	10	35	32	[40]
	major	350	44	41	126	116	
	total		55	51	161	148	
$(C_{14})_2PbI_4$	*minor*	329	11	9	32	28	[40]
	major	360	55	48	152	133	
	total		65	57	184	161	
$(C_{16})_2PbI_4$	*minor*	340	12	10	34	28	[40]
	major	369	63	52	170	142	
	total		74	62	204	170	

[a]$C_n = C_nH_{2n+1}NH_3$. [b]When a compound displays multiple transitions, the transition with the highest ΔS was labeled as a major transition. [c]Measured during the first heating scan. Note that the organic bilayers of 2-D Pb–I perovskites are partially interdigitated.

Supplementary Table 4. Prediction of barocaloric coefficients of representative two-dimensional $(C_nH_{2n+1}NH_3)_2MCl_4$ perovskites (M = Mn, Cu).

**Chemical Formula**[a]	**Type**	T_{tr} **(K)**	**Δ*d* (Å)**	**Estimated** ΔV_{tr}[b] **(cm³ kg⁻¹)**	ΔS_{tr} **(J K⁻¹ kg⁻¹)**	**Estimated** dT/dP[c] **(K kbar⁻¹)**	**Ref.**
$(C_{12})_2MnCl_4$	*major*	331	29.7 to 31.9; 2.2 (7.4%)	61.7	254	24.3	[35]
	minor	335	31.9 to 32.2; 0.3 (0.9%)	8.4	31	27.1	
$(C_{14})_2MnCl_4$	*major*	345	33.3 to 35.5; 2.2 (6.6%)	56.1	268	20.9	[35]
	minor	357	35.5 to 36.4; 0.9 (2.5%)	23.0	41	56.0	
$(C_{10})_2CuCl_4$	*major*	309	25.2 to 27.0; 1.8 (7.1%)	57.1	216	26.4	[41]
	minor	312	27.0 to 27.6; 0.6 (2.2%)	19.0	28	68.0	
$(C_{12})_2CuCl_4$	*major*	328	28.8 to 30.3; 1.5 (5.2%)	43.0	210	20.5	[35]
	minor	334	30.3 to 31.1; 0.8 (2.6%)	22.9	43	53.3	
$(C_{14})_2CuCl_4$	*major*	334	32.9 to 34.5; 1.6 (4.9%)	41.8	237	17.6	[35]
	minor	356	34.5 to 35.5; 1.0 (2.9%)	26.1	48	54.4	

[a] $C_n = C_nH_{2n+1}NH_3$. [b] Note that the volume change of the phase transition was estimated using $\Delta V_{tr} = (\Delta d \cdot A) \times N_A / M_w$, where A is the area of metal–chloride sheet per chain, Δd is the change in interlayer distance, M_w is the molecular weight, and N_A is Avogadro's number. This estimation was used because the unit cell parameters of intermediate phases were often not available. Note that A is 26.5 Å² and 27.5 Å² for Mn–Cl perovskites and Cu–Cl perovskites[29]. [c] Barocaloric coefficients were calculated using the Clausius–Clapeyron equation ($dT/dP = \Delta V_{tr}/\Delta S_{tr}$). Here, the order-disorder phase transitions of these compounds are predicted to be highly sensitive to pressure, with minor transitions particularly more sensitive than major transitions owing to their much lower entropy changes.

Supplementary Table 5. Phase-change properties and barocaloric effects of representative barocaloric materials shown in Fig. 4.

Type	Chemical Formula[a]	T_{tr}[b] (K)	ΔS_{tr}[c] (J kg^{-1} K^{-1})	dT/dP *heating* (K kbar^{-1})	dT/dP *cooling* (K kbar^{-1})	ΔT_{hys}[d] (K)	P_{rev}[e] (bar)	$\Delta S_{it,rev}$[f] (J kg^{-1} K^{-1})	ΔP[f] (bar)	Ref.
2-D perovskites	$(DA)_2MnCl_4$[g]	310	230	22.5 (22.1)[h]	20.2 (20.6)	1.4 *onset*	92 (66)	190 (75)	150	*this work*
	$(NA)_2CuBr_4$[g]	305	76	26.7 (26.9)[h]	26.7 (26.5)	0.4 *onset*	38 (16)	78 (68)	150	
3-D hybrid perovskites	$[(CH_3)_4N][Mn(N_3)_3]$	305	80	12[i]		7 *peak*	583	70	900	[42]
	[TPrA][Mn(dca)$_3$]	330	43	23.1[i]		0.9 *peak*	39	31	70	[43]
	[TPrA][Cd(dca)$_3$]	386	16	38.2[i]		1.4 *peak*	37	11.5	70	[44]
Organic plastic crystals	$(CH_3)_2C(CH_2OH)_2$	314	389	11.3	9.3	14–25[j] *peak*	1505	445	2000	[10, 45,46]
	$(CH_3)C(CH_2OH)_3$	354	485	7.9	9.4	3.7 *onset*	394	490	2400	[10]
	$(CH_3)_3C(CH_2OH)$	232	204	22	11.9	20.3 *onset*	1706	290	2600	[10]
	C_{60}	259	27	16.7	17.2	3 *peak*	174	32 [42]	1000 [4100]	[47]
Inorganic salts	$(NH_4)_2SO_4$	222	65	–5.7	–4.5	1 *onset*	175	60	1000	[48]
	AgI	420	64	–14	–12.8	25 *peak*	1786	60	1000	[49]
	$Ni_{0.85}Fe_{0.15}S$[k]	303	53	–7.5		11.5 *peak*	1533			[50]
Alloys	$Fe_{49}Rh_{51}$	310	13	5.4	6.4	10 *peak*	1563	13	2500	[51]
	$Ni_{50}Mn_{31.5}Ti_{18.5}$	255	85	1.9	3.2	12 *peak*	3750	35	3800	[52]
Spin-crossover complexes	$Fe_3(bntrz)_6(tcnset)_6$	318	80	25	25	2 *peak*	80	80 [120]	550 [2600]	[53]
	$[FeL_2](BF_4)_2$[k]	262	86	10[l]	10[l]	4 *peak*	400			[54]

[a]DA = decylammonium; NA = nonylammonium; TPrA = tetrapropylammonium; bntrz = 4-(benzyl)-1,2,4-triazole; tcnset = 1,1,3,3-tetracyano-2-thioethylepropenide; L = 2,6-di(pyrazol-1-yl)pyridine. [b]Transition temperatures measured during heating are tabulated here. [c]Entropies of transitions measured at ambient pressure are tabulated here. [d]$\Delta T_{hys} = T_{tr,\,heating} - T_{tr,\,cooling}$ at ambient pressure. The transition temperatures were identified as transition onset or peak values. [e]Calculated using $P_{rev} = \Delta T_{hys}$ / |dT/dP|, with dT/dP values for exothermic and endothermic transitions used for normal and inverse barocaloric materials, respectively. For ΔT_{hys} determined from peak values, the P_{rev} values calculated here may be overestimated, but the magnitude of this overestimation should be minimal for compounds with sharp DSC peaks or large hysteresis values. Note that inverse barocaloric materials refer to compounds with dT/dP < 0. [f]Reversible isothermal entropy changes, $\Delta S_{it,\,rev}$, at the driving pressure ΔP are tabulated here. Note that these values were derived from quasi-direct measurements[11,55]. At high pressures (typically above 1 kbar), the additional entropy changes outside of the transition, ΔS_{+}, contribute to ΔS_{it} values, leading to ΔS_{it} values that can exceed ΔS_{tr}. [g]The values determined from both powder and single crystal samples are tabulated, with the values for the powder samples in parentheses. [h]Barocaloric coefficients calculated from the Clausius–Clapeyron equation (dT/d$P = \Delta V_{tr}/\Delta S_{tr}$) are predicted to be 29 and 33 K kbar^{-1} for $(DA)_2MnCl_4$ and $(NA)_2CuBr_4$, respectively (Supplementary Table 10). [i]dT/dP values were averaged from heating and cooling data. [j]ΔT_{hys} depends on scan rates. The smaller value of 14 K was used for the calculation of P_{rev}. [k]Only an irreversible ΔS_{it} value is reported ($Ni_{0.85}Fe_{0.15}S$, 53 J kg^{-1} K^{-1} at 1000 bar; $[FeL_2](BF_4)_2$, 68 J kg^{-1} K^{-1} at 430 bar). [l]dT/dP values obtained at a pressure range < 2 kbar values are shown, with (dT/dP)$_{heating}$ and (dT/dP)$_{cooling}$ obtained from calorimetry and SQUID magnetometry, respectively.

Supplementary Table 6. Barocaloric effects and predicted thermodynamic efficiencies for selected barocaloric materials.

Chemical Formula[a]	T_{tr} [c] (K)	$\Delta S_{it,rev}$ / ΔP [d] (J kg^{-1} K^{-1} kbar^{-1})	$\Delta T_{ad,max}$ [e] (K)	ΔT_{hys} / $\Delta T_{ad,max}$ [f] (%)	η [g] (%)	Ref.
$(DA)_2MnCl_4$[b]	310	1267	45[h]	5.3	82	*this work*
$(NA)_2CuBr_4$[b]	305	884	21[h]	6.7	79	
[TPrA][Mn(dca)$_3$]	330	443	5[i]	15.8	61	[43]
$(CH_3)_2C(CH_2OH)_2$	314	225	45[j]	31.1	45	[10, 45,46]
C_{60}	259	32	20[k]	15.0	63	[47]
$(NH_4)_2SO_4$	222	60	8[l]	12.5	67	[48]
$Ni_{0.85}Fe_{0.15}S$	303		30[m]	38.3	40	[50]
Fe_3(bntrz)$_6$(tcnset)$_6$	318	267	35[n]	5.7	81	[53]

[a]DA = decylammonium; NA = nonylammonium; TPrA = tetrapropylammonium; bntrz = 4-(benzyl)-1,2,4-triazole; tcnset = 1,1,3,3-tetracyano-2-thioethylepropenide. [b]The values obtained from single-crystal samples are tabulated here. [c]Transition temperatures measured during heating are tabulated here. [d]The reversible isothermal entropy change $\Delta S_{it,rev}$ normalized by the driving pressure, often referred to as barocaloric strength[55], are tabulated here. For consistency and due to limitations of available data from previous reports, the barocaloric strength values for all compounds were determined by choosing the smallest ΔP values that capture the maximum entropy of the transition. For instance, barocaloric strength values for $(DA)_2MnCl_4$ and $(NA)_2CuBr_4$ were determined at pressures near $P_{rev,ad}$ using $\Delta S_{it,rev}$ of 190 J kg^{-1} K^{-1} at a driving pressure of 150 bar and $\Delta S_{it,rev}$ of 71 J kg^{-1} K^{-1} at a driving pressure of 80 bar, respectively. [e]Maximum adiabatic temperature changes tabulated here were calculated using the indirect method, with $\Delta T_{ad,max} = -T\Delta S_{it}/c_p$, or, by quasi-direct methods[55]. [f]For consistency with the previously reported model for calculating an idealized thermodynamic efficiency based on dissipative heat losses due to hysteresis in a Carnot-like cycle[56], ΔT_{hys} values calculated as the difference between heating and cooling peak temperatures were used for consistency. For 2-D perovskites, ΔT_{hys} values determined from the transition peaks are shown in Supplementary Table 7. [g]The second-law efficiency η, which corresponds to the coefficient of performance (COP) of material with hysteresis normalized by COP for a Carnot cycle (COP_{Carnot}), is estimated using the equation $\eta = COP/COP_{Carnot} = \frac{1}{1+4\frac{\Delta T_{hys}}{\Delta T_{ad,max}}}$ (ref. 56). Note that, in this model, the thermal hysteresis is defined as the difference between heating and cooling transition peak temperatures for c_P/T curves, which corresponds to the width of thermal hysteresis loop. [h]Estimated using the $\Delta T_{ad} = -T\Delta S_{it}/c_P$. For $(DA)_2MnCl_4$, ΔS_{it} of 230 J kg^{-1} K^{-1} at 300 bar, with T = 312 K and c_p = 1550 J kg^{-1} K^{-1} are used (Supplementary Fig. 31a). For $(NA)_2CuBr_4$, ΔS_{it} of 79 J kg^{-1} K^{-1} at 300 bar, with T = 309 K and c_p = 1180 J kg^{-1} K^{-1} are used (Supplementary Fig. 31b). From the quasi-direct analyses, $(DA)_2MnCl_4$ DA)$_2MnCl_4$ and $(NA)_2CuBr_4$ are predicted to reach the maximum ΔT_{ad} of ~45 K and ~21 K at ~2,500 bar and ~920 bar, respectively (Supplementary Fig. 33). Note that reversible $\Delta T_{ad,rev}$ of 2.3 K was achieved from the single-crystal $(NA)_2CuBr_4$ from the quasi-direct method (Supplementary Fig. 17g). [i]Estimated using the indirect method, with $\Delta S_{it,rev}$ of 35 J kg^{-1} K^{-1} from a pressure change of 69 bar with T = 332 K and c_p = 2450 J kg^{-1} K^{-1}. [j]Quasi-direct measurements at a driving pressure of 5700 bar (ref. 46). [k]Quasi-direct measurements at a driving pressure of 5900 bar. [l]Estimated using the indirect method, with $\Delta S_{it,rev}$ of 60 J kg^{-1} K^{-1} at a driving pressure of 1000 bar with c_p = 1700 J kg^{-1} K^{-1}. [m]Estimated using the indirect method, with the irreversible ΔS_{it} value of 53 J kg^{-1} K^{-1} at a driving pressure of 1000 bar. [n]Quasi-direct measurements at a driving pressure of 2600 bar.

Supplementary Table 7. Summary of low-pressure HP-DSC experiments for single-crystal and powder samples. The values obtained during cooling are shown in parentheses after values obtained during heating.

	**Sample**	**ΔT_{width} (K)**	**ΔT_{hys} (K)**	**dT/dP (K kbar^{-1})**	**P_{rev}[c] (bar)**	**$P_{rev,ad}$[d] (bar)**	**$\Delta S_{it,rev,max}$[e] (J kg^{-1} K^{-1})**	**RC_{rev}[f] (J kg^{-1})**
$(DA)_2MnCl_4$	*crystals*	0.9 (0.7)	1.7[a] [2.4][b]	22.5 (20.2)	92	178	190	103
	powder	2.3 (2.0)	1.4[a] [3.5][b]	22.1 (20.6)	66	265	75	51
$(NA)_2CuBr_4$	*crystals*	0.5 (0.4)	1.0[a] [1.4][b]	26.7 (26.7)	38	73	78	188
	powder	1.7 (1.8)	0.4[a] [2.2][b]	26.9 (26.5)	16	148	68	124

[a]calculated as the difference between heating and cooling transition onset temperatures, with $\Delta T_{hys} = T_{tr,heating} - T_{tr,cooling}$. [b]calculated as the difference between heating and cooling peak temperatures, with $\Delta T_{hys} = T_{peak,heating} - T_{peak,cooling}$. [c]pressure required to capture a non-zero isothermal entropy change, which is calculated as $\Delta T_{hys}/(\mathrm{d}T/\mathrm{d}P)_{cooling}$. [d]pressure required to capture the full phase-change entropy reversibly and induce a non-zero adiabatic temperature change, which is calculated as $(\Delta T_{width,heating} + \Delta T_{width,heating} + \Delta T_{hys})/(\mathrm{d}T/\mathrm{d}P)_{cooling}$. [e]maximum reversible entropy change at an operating pressure of 150 bar. [f]reversible refrigeration capacity (RC_{rev}), which corresponds to the area under reversible isothermal entropy peak and is calculated as $\Delta S_{it,rev,max} \times \Delta T_{FWHM}$.

Supplementary Table 8. Barocaloric coefficients measured from HP-DSC and *in situ* PXRD experiments for $(DA)_2MnCl_4$ and $(NA)_2CuBr_4$.

	Sample	Method	**d*T*/d*P* (K kbar^{-1})** *heating*	**d*T*/d*P* (K kbar^{-1})** *cooling*	**Fitting range** (bar)
$(DA)_2MnCl_4$	*crystals*	HP-DSC	22.5 ± 0.3 (R^2 = 0.999)	20.2 ± 1.1 (R^2 = 0.988)	1–150
	powder	HP-DSC	22.1 ± 0.6 (R^2 = 0.996)	20.6 ± 0.8 (R^2 = 0.994)	1–150
		HP-DSC[a]	19.1 ± 0.2 (R^2 = 0.999)	18.4 ± 0.4 (R^2 = 0.998)	1–500
		PXRD		18.8 ± 2.1 (R^2 = 0.999)	1–360
		combined[a] *linear*	18.7 ± 0.3 (R^2 = 0.996)	18.1 ± 0.3 (R^2 = 0.997)	1–500
		combined[a] *quadratic*	(21.3 ± 0.9) + (–11.0 ± 4.0)P (R^2 = 0.998)	(20.1 ± 1.1) + (–8.4 ± 4.6)P (R^2 = 0.998)	
$(NA)_2CuBr_4$	*crystals*	HP-DSC	26.7 ± 0.4 (R^2 = 0.998)	26.7 ± 0.4 (R^2 = 0.998)	1–150
	powder	HP-DSC	26.9 ± 0.4 (R^2 = 0.998)	26.5 ± 0.5 (R^2 = 0.998)	1–150
		HP-DSC	24.0 ± 0.2 (R^2 = 0.999)	24.2 ± 0.3 (R^2 = 0.999)	1–500
		PXRD		25.5 ± 3.2 (R^2 = 0.983)	1–300
		combined *linear*	23.7 ± 0.3 (R^2 = 0.998)	24.1 ± 0.2 (R^2 = 0.998)	1–500
		combined *quadratic*	(26.6 ± 0.8) + (–12.2 ± 3.4)P (R^2 = 0.999)	(26.7 ± 0.5) + (–11.0 ± 2.2)P (R^2 = 0.999)	

[a]Only the onset temperatures for major transitions were used for fitting. For the pressure range between 1 and 150 bar, the barocaloric coefficient of $(NA)_2CuBr_4$ is 22% and 29% larger than that of $(DA)_2MnCl_4$, for heating and cooling, respectively.

Supplementary Table 9. Summary of isothermal compressibility, thermal expansion coefficient, ΔS_+, and ΔS_V for $(DA)_2MnCl_4$ and $(NA)_2CuBr_4$.

**Compound**	**Phase**	**κ (×10^{-4} MPa^{-1})[a]**	**α (×10^{-4} K^{-1})[b]**	**ΔS_+[c] (J kg^{-1} K^{-1})**	**ΔS_V[e] (J kg^{-1} K^{-1})** *HT phase*	**ΔS_V[e] (J kg^{-1} K^{-1})** *averaged*
$(DA)_2MnCl_4$	LT	1.6(4)	2.7(3)	−3 (150 bar)<br>−11 (500 bar)	92 ± 24 (40%)	98 ± 18 (42%)
	HT	3.2(5)	4.5(9)	−6 (150 bar)<br>−21 (500 bar)		
$(NA)_2CuBr_4$	LT	1.6(4)[d]	4(1)	−4 (150 bar)<br>−13 (500 bar)	37 ± 6 (48%)	44 ± 8 (58%)
	HT	4.2(7)	6.2(3)	−6 (150 bar)<br>−20 (500 bar)		

[a]Isothermal compressibility was calculated as $\kappa = -[V^{-1}(\partial V/\partial P)_T]$, using the average pressure dependence of the specific volume shown in Supplementary Fig. 5. [b]Thermal expansion coefficients were calculated as $\alpha = [V^{-1}(\partial V/\partial T)_P]$, using the temperature dependence of the specific volume shown in Supplementary Fig. 4. Note that all thermal expansion coefficients were determined at ambient pressure. [c]Additional entropy changes, ΔS_+, outside of the first-order phase transition were estimated using $\Delta S_+ = -(V{\cdot}\alpha){\cdot}\Delta P$, where V, ΔP, and α denote the specific volume, driving pressure, and thermal expansion coefficient at ambient pressure, respectively. [d]Calculated using isobaric data. [e]Volumetric entropy changes (ΔS_V) were estimated using $\Delta S_V = (\alpha/\kappa){\cdot}\Delta V_{tr}$, where α, κ, and ΔV_{tr} denote the thermal expansion coefficient at ambient pressure, isothermal compressibility, and the specific volume change of the phase transition at ambient pressure. Note that this formula is derived from the Maxwell relation $(\partial S/\partial V)_P = (\partial P/\partial T)_V$. ΔS_V values are calculated through two methods: 1) using α and κ values from the HT phase only, and 2) using α and κ values averaged across the phase transition. The relative contribution of ΔS_V to the total transition entropy ΔS_{tr} is shown in parentheses. A discussion of the calculation of ΔS_V is provided in Supplementary Note 3.

Supplementary Table 10. Comparison of predicted and experimentally determined barocaloric coefficients (dT/dP). Note that predicted barocaloric coefficients were calculated using the Clausius–Clapeyron equation (dT/dP = $\Delta V_{tr}/\Delta S_{tr}$), with ΔV_{tr} determined through either powder X-ray diffraction or He pycnometry, and ΔS_{tr} measured at ambient pressure.

Compound	PXRD[a] ΔV_{tr} (cm^3 kg^{-1})	PXRD[a] dT/dP (K kbar^{-1}) *predicted*		He pycnometry[b] ΔV_{tr} (cm^3 kg^{-1}) *heating*	He pycnometry[b] ΔV_{tr} (cm^3 kg^{-1}) *cooling*	He pycnometry[b] dT/dP (K kbar^{-1}) *predicted* *heating*	He pycnometry[b] dT/dP (K kbar^{-1}) *predicted* *cooling*	HP-DSC dT/dP (K kbar^{-1}) *experimental* *heating*	HP-DSC dT/dP (K kbar^{-1}) *experimental* *cooling*
$(DA)_2MnCl_4$	65.9 (7.97%)	28.7	linear fit	54.7 (6.6%)	53.1 (6.4%)	23.8	23.1	22.1 ± 0.7	20.6 ± 0.8
			nearest points	49.3 ± 3.4 (5.9%)	47.5 ± 5.8 (5.7%)	21.4 ± 1.5	20.7 ± 2.5		
$(NA)_2CuBr_4$	25.3 (4.01%)	33.3	linear fit	19.3 (3.1%)	19.3 (3.1%)	25.4	25.3	26.9 ± 0.4	26.5 ± 0.5
			nearest points	20.0 ± 1.9 (3.2%)	19.7 ± 2.4 (3.1%)	26.3 ± 2.5	26.0 ± 3.1		

[a]The volume at T_{tr} for the LT and HT phases was extrapolated from unit cells determined below and above T_{tr}, respectively, using thermal expansion coefficients for each phase determined at ambient pressure. [b]The volume at T_{tr} for the LT and HT phases was either extrapolated using the thermal expansion coefficients of each phase ("linear fit") or calculated choosing two nearest points before and after the onset transition temperature.

Supplementary Table 11. Summary of thermodynamic data from quasi-isothermal HP-DSC experiments.

**Sample**	$\boldsymbol{T_{set}}$ **(K)**	$\boldsymbol{\Delta P_{hys}}$ **(bar)**	$\boldsymbol{q_{comp}}$ **(J g^{-1})**	$\boldsymbol{q_{decomp}}$ **(J g^{-1})**	$\boldsymbol{\Delta S_{it,comp}}$ **(J kg^{-1} K^{-1})**	$\boldsymbol{\Delta S_{it,decomp}}$ **(J kg^{-1} K^{-1})**
$(DA)_2MnCl_4$	311.1	62(4)	58.0(5)	63(2)	186(2)	203(7)
$(NA)_2CuBr_4$	305.5	18(1)	19(2)	21.4(4)	61(7)	60(1)

Note that the q_{comp} and q_{decomp} correspond to the enthalpy changes measured during quasi-isothermal calorimetry experiments for compression/pressurization and decompression/depressurization, respectively. Similarly, $\Delta S_{it,comp}$ and $\Delta S_{it,decomp}$ correspond to the entropy changes measured during quasi-isothermal calorimetry experiments for compression/pressurization and decompression/depressurization, respectively. Although direct comparisons are challenging (mostly due to the challenges in maintaining perfect isothermality after the phase transition is induced), the isothermal entropy changes obtained from quasi-isothermal experiments are in good agreement with the values calculated from isobaric experiments using the quasi-direct method (Supplementary Table 7), with $\Delta S_{it,comp}$ and $\Delta S_{it,decomp}$ between the single-crystal (upper bound) and powder (lower bound) values.

Supplementary Table 12. Summary of volume changes (ΔV_{tr}) during the order-disorder transition determined by isobaric powder X-ray diffraction.

**Compound**	**Pressure (bar)**	$V_{LT,tr}$ **(cm^3 kg^{-1})**	$V_{HT,tr}$ **(cm^3 kg^{-1})**	ΔV_{tr} **(cm^3 kg^{-1})**
$(DA)_2MnCl_4$	1	827.2	893.1	65.9 (7.97%)
	200	825.4	888.7	63.3 (7.67%)
	300	824.5	886.4	61.9 (7.51%)
	360	823.9	885.1	61.2 (7.42%)
$(NA)_2CuBr_4$	1	630.5	655.8	25.3 (4.01%)
	200[a]	630.5	652.8	22.3 (3.52%)
	250[a]	630.3	651.7	21.4 (3.39%)
	300	630.3	650.9	20.6 (3.27%)

[a]estimated using isothermal compressibility

Supplementary Table 13. Summary of characteristic infrared bands for $(DA)_2MnCl_4$ and $(NA)_2CuBr_4$ in the low-temperature (LT) and high-temperature (HT) phases.

**Structure**	**Mode**[a] (cm^{-1})	**$(DA)_2MnCl_4$** LT band (cm^{-1})	**$(DA)_2MnCl_4$** HT band (cm^{-1})	**$(NA)_2CuBr_4$** LT band (cm^{-1})	**$(NA)_2CuBr_4$** HT band (cm^{-1})
**Alkyl chain** ($\mathbf{C_nH_{2n+1}}NH_3^+$)	ν_{as}(C–H)	2919	2919	2919	2920
	ν_s(C–H)	2850	2852	2851	2853
	$\nu(CH_2)_{rocking}$	727–720	721	722	722
	$\nu(CH_2)_{bending}$	1472–1463	1464	1467	1468
	$\nu(CH_2)_{wagging}$ *two units, in-plane* 1310 (*kink*)		1310	1306	1306 (*broad*); 1312
	$\nu(CH_2)_{wagging}$ *two units, out-of-plane* 1360 (*kink*)		1367	1360	
	$\nu(CH_2)_{wagging}$ *single unit* 1350 (*gg*)				
	$(CH_2)_{wagging}$ *single unit* 1340 (*tg*, chain end)			1340	
	$\nu_s(CH_3)_{bending}$	1375	1379	1378	1379
**Polar headgroup** ($C_nH_{2n+1}\mathbf{NH_3^+}$)	$\nu_s(NH_3)_{bending}$	1496	1496 (*broad*)	1480	1480
	$\nu_{as}(NH_3)_{bending}$	1585	1579 (*broad*)	1569–1572	1569

[a] ν_s and ν_{as} refer to symmetric and anti-symmetric modes, respectively.
[b] previous reports on Cd–Cl and Mn–Cl analogs revealed that the peak near 1337 cm^{-1} does not depend on chain conformation[3,22].

Supplementary Table 14. Unit cell parameters for $(DA)_2MnCl_4$ from Le Bail refinements.

T **(K)**	***P*** **(bar)**	***a*** **(Å)**	***b*** **(Å)**	***c*** **(Å)**	***α*** **(°)**	***β*** **(°)**	***γ*** **(°)**	***V*** **(Å³)**	**Space Group**
280	1	7.1974(2)	7.3217(1)	26.658(1)	90	94.698(3)	90	1400.14(8)	*C*2/*m*
330	1	7.2800(5)	7.3374(4)	57.561(3)	90	90	90	3074.7(3)	*Cccm*
280	300	7.1973(2)	7.3198(2)	26.649(2)	90	94.5516(4)	90	1399.5(1)	*C*2/*m*
330	300	7.2758(4)	7.3329(4)	56.957(4)	90	90	90	3038.8(3)	*Cccm*
280	360	7.2094(4)	7.3234(3)	26.653(2)	90	94.504(7)	90	1402.8(2)	*C*2/*m*
330	360	7.2722(5)	7.3304(4)	56.686(4)	90	90	90	3021.8(3)	*Cccm*

Supplementary Table 15. Unit cell parameters for $(NA)_2CuBr_4$ from Le Bail refinements.

T **(K)**	***P*** **(bar)**	***a*** **(Å)**	***b*** **(Å)**	***c*** **(Å)**	***α*** **(°)**	***β*** **(°)**	***γ*** **(°)**	***V*** **(Å^3)**	**Space Group**
280	1	7.6362(7)	8.0165(8)	23.027(1)	83.06(1)	84.042(6)	89.633(6)	1391.7(2)	$P\bar{1}$
330	1	7.917(1)	7.786(1)	48.241(2)	90	90	90	2973.7(5)	*Cmca*
280	120	7.6363(5)	8.0185(6)	22.959(1)	82.947(7)	83.959(5)	89.640(4)	1387.4(1)	$P\bar{1}$
314	120	7.9280(5)	7.7545(4)	47.647(1)	90	90	90	2929.2(2)	*Cmca*
280	200	7.6307(7)	8.0191(8)	22.982(1)	82.97(1)	83.980(7)	89.648(7)	1388.0(2)	$P\bar{1}$
330	200	7.9125(7)	7.7758(7)	47.896(1)	90	90	90	2946.9(4)	*Cmca*
280	300	7.6264(9)	8.0142(1)	22.979(2)	83.01(1)	83.98(1)	89.65(1)	1386.3(3)	$P\bar{1}$
330	300	7.9058(9)	7.7712(8)	47.711(2)	90	90	90	2931.3(4)	*Cmca*

Supplementary Table 16. Selected geometric parameters from the single crystal structures of $(DA)_2MnCl_4$ at 100 K, 270 K, and 330 K.

Temperature (K)	100 K	270 K	330 K
Space Group	$P\bar{1}$	*C*2/*m*	*Cccm*
V (Å^3)	672.07(14)	1396.0 (2)	3069.5 (9)
Nearest Mn–Mn (Å)	5.0548(6)	5.1252 (3)	5.1619 (3)
Nearest N–N (Å)	5.0548(6)	4.9369 (3)	5.0726 (3)
Interlayer distance (Å)	26.419(3)	26.685 (2)	28.800 (1)
Distance between N atoms and $[MnCl_4]^{2-}$ (Å)[a]	2.325(4)	2.305 (1)	2.262 (1)
Chain tilt angle relative to $[MnCl_4]^{2-}$ sheet normal (°) *best line* (C1 to C10) [disordered position]	42.8(10) [42.8(10)]	41.7 (1)	25.8 (1)
Chain tilt angle relative to $[MnCl_4]^{2-}$ sheet normal (°) *mean plane* (C2 to C10) [disordered position]	65.1(10) [65.4(10)]	70.9 (1)	69.5 (1)
Cross-sectional area per chain (Å^2)[b]	25.439(6)	26.157(4)	26.635(8)
Cross-sectional area per chain (Å^2)[c]	25.551(4)	26.268(2)	26.645(2)

[a]calculated as the distance between the mean plane of four N atoms and the mean plane of the Mn–Cl layer.
[b]calculated from $V / (Z \cdot d)$, where V is the unit cell volume, Z is the number of molecules in the unit cell, and d is the interlayer distance.
[c]stimated as d_M^2, where d_M is the nearest Mn–Mn distance.

Supplementary Table 17. Selected geometric parameters from the single crystal structures of $(NA)_2CuBr_4$ at 100 K, 270 K, and 335 K.

Temperature (K)	100 K	270 K	335 K
Space Group	$P\bar{1}$	$P\bar{1}$	*Cmca*
V (Å^3)	1330.3(2)	1382.70(7)	2966.0(3)
Nearest Cu–Cu (Å)	5.4102(4)	5.5173(1)	5.4927(3)
Nearest N–N (Å)	5.117(7)	5.1667(1)	5.4927(3)
Interlayer distance (Å)	23.054(2)	23.254(1)	24.578(2)
Distance between N atoms and $[CuBr_4]^{2-}$ (Å)[a]	2.201(4)	2.150(1)	2.138(1)
Chain tilt angle relative to $[CuBr_4]^{2-}$ sheet normal (°) *best line* (C1 to C9) [disordered position]	46.9(2)	46.67(1) [43.37(1)]	35.4 (1)
Chain tilt angle relative to $[CuBr_4]^{2-}$ sheet normal (°) *best line* (C11 to C19) [disordered position]	48.7(2)	48.49(1) [47.00(1)]	
Chain tilt angle relative to $[CuBr_4]^{2-}$ sheet normal (°) *mean plane* (C2 to C9) [disordered position]	58.9(3)	64.60(1) [72.16(1)]	68.4 (1)
Chain tilt angle relative to $[CuBr_4]^{2-}$ sheet normal (°) *mean plane* (C12 to C19) [disordered position]	54.8(3)	52.36 (1) [54.72 (1)]	
Cross-sectional area per chain (Å^2)[b]	28.852(5)	29.730(2)	30.169(4)
Cross-sectional area per chain (Å^2)[c]	29.270(3)	30.440(7)	30.169(2)

[a]calculated as the distance between the mean plane of four N atoms and the mean plane of the Cu–Br layer.
[b]calculated from $V / (Z \cdot d)$, where V is the unit cell volume, Z is the number of molecules in the unit cell, and d is the interlayer distance.
[c]stimated as d_M^2, where d_M is the nearest Cu–Cu distance.

Supplementary Table 18. Dihedral angles, φ, of alkylammonium chains at 100 K, 270 K, and 330 K for $(DA)_2MnCl_4$.

T (K)	**Atoms** *Part 1*[a]	φ (°) *Part 1*	**Occupancy** *Part 1*	**Atoms** *Part 2*[a]	φ (°) *Part 2*	**Occupancy** *Part 2*
100	N1–C1–C2–C3	179.6 (10)	0.504(13)	N1A–C1A–C2A–C3A	178.2 (10)	0.496(13)
	C1–C2–C3–C4	–68 (2)	0.504(13)	C1A–C2A–C3A–C4A	70 (2)	0.496(13)
	C2–C3–C4–C5	–178.2 (13)	0.504(13)	C2A–C3A–C4A–C5A	177.5 (14)	0.496(13)
	C3–C4–C5–C6	178.0 (14)	0.504(13)	C3A–C4A–C5A–C6A	–178.4 (15)	0.496(13)
	C4–C5–C6–C7	–179.7 (15)	0.504(13)	C4A–C5A–C6A–C7A	–179.3 (16)	0.496(13)
	C5–C6–C7–C8	178.6 (14)	0.504(13)	C5A–C6A–C7A–C8A	–179.3 (15)	0.496(13)
	C6–C7–C8–C9	178.4 (15)	0.504(13)	C6A–C7A–C8A–C9A	177.7 (16)	0.496(13)
	C7–C8–C9–C10	175.9 (17)	0.504(13)	C7A–C8A–C9A–C10A	179.3 (17)	0.496(13)
270	N1–C1–C2–C3	174 (3)				
	C1–C2–C3–C4	–65 (5)				
	C2–C3–C4–C5	179 (3)				
	C3–C4–C5–C6	177 (4)				
	C4–C5–C6–C7	–175 (5)				
	C5–C6–C7–C8	180 (5)				
	C6–C7–C8–C9	–174 (5)				
	C7–C8–C9–C10	–169 (8)				
330	N1–C1–C2–C3	174 (3)				
	C1–C2–C3–C4	–166 (3)				
	C2–C3–C4–C5	–180 (3)				
	C3–C4–C5–C6	163 (4)				
	C4–C5–C6–C7	154 (4)				
	C5–C6–C7–C8	160 (4)				
	C6–C7–C8–C9	165 (5)				
	C7–C8–C9–C10	–151 (5)				

[a]Part 1 and part 2 refer to the sets of disordered positions of the decylammonium chains.

Supplementary Table 19. Dihedral angles, *φ*, of alkylammonium chains at 100 K, 270 K, and 335 K for $(NA)_2CuBr_4$.

T **(K)**	**Atoms** *Part 1*[a]	***φ*** **(°)** *Part 1*	**Occupancy** *Part 1*	**Atoms** *Part 2*[a]	***φ*** **(°)** *Part 2*	**Occupancy** *Part 2*
100	N1–C1–C2–C3	–175.0 (6)				
	C1–C2–C3–C4	–65.7 (9)				
	C2–C3–C4–C5	–177.4 (7)				
	C3–C4–C5–C6	178.8 (7)				
	C4–C5–C6–C7	179.9 (6)				
	C5–C6–C7–C8	179.0 (7)				
	C6–C7–C8–C9	177.9 (7)				
	N2–C11–C12–C13	70.0 (7)				
	C11–C12–C13–C14	–177.6 (6)				
	C12–C13–C14–C15	174.1 (6)				
	C13–C14–C15–C16	178.6 (6)				
	C14–C15–C16–C17	–179.5 (6)				
	C15–C16–C17–C18	–175.1 (6)				
	C16–C17–C18–C19	–177.3 (7)				
270	N1–C1–C2–C3	–173.0 (17)	0.528(9)	N1A–C1A–C2A–C3A	172 (2)	0.472(9)
	C1–C2–C3–C4	–39 (3)	0.528(9)	C1A–C2A–C3A–C4A	–178 (3)	0.472(9)
	C2–C3–C4–C5	–176 (3)	0.528(9)	C2A–C3A–C4A–C5A	174 (3)	0.472(9)
	C3–C4–C5–C6	178 (3)	0.528(9)	C3A–C4A–C5A–C6A	180 (4)	0.472(9)
	C4–C5–C6–C7	178 (4)	0.528(9)	C4A–C5A–C6A–C7A	173 (4)	0.472(9)
	C5–C6–C7–C8	168 (4)	0.528(9)	C5A–C6A–C7A–C8A	–174 (4)	0.472(9)
	C6–C7–C8–C9	164 (4)	0.528(9)	C6A–C7A–C8A–C9A	–164 (4)	0.472(9)
	N2–C11–C12–C13	–67 (5)	0.350(15)	N2A–C11A–C12A–C13A	77 (2)	0.650(15)
	C11–C12–C13–C14	173 (4)	0.350(15)	C11A–C12A–C13A–C14A	–176.9 (19)	0.650(15)
	C12–C13–C14–C15	–159 (5)	0.350(15)	C12A–C13A–C14A–C15A	169 (2)	0.650(15)
	C13–C14–C15–C16	–175 (5)	0.350(15)	C13A–C14A–C15A–C16A	–180 (2)	0.650(15)
	C14–C15–C16–C17	–173 (6)	0.350(15)	C14A–C15A–C16A–C17A	175 (3)	0.650(15)
	C15–C16–C17–C18	–177 (6)	0.350(15)	C15A–C16A–C17A–C18A	176 (3)	0.650(15)
	C16–C17–C18–C19	159 (6)	0.350(15)	C16A–C17A–C18A–C19A	–170 (3)	0.650(15)
335	N1–C1–C2–C3	156 (3)				
	C1–C2–C3–C4	44 (7)				
	C2–C3–C4–C5	156 (4)				
	C3–C4–C5–C6	–128 (8)				
	C4–C5–C6–C7	160 (7)				
	C5–C6–C7–C8	174 (6)				
	C6–C7–C8–C9	–142 (10)				

[a]Part 1 and part 2 refer to the two sets of disordered positions of the nonylammonium chains.

Supplementary Table 20. Donor–acceptor (N···X) distances and bond angles for the LT and HT phases of $(DA)_2MnCl_4$ and $(NA)_2CuBr_4$

Compound	*T* (K)	N–H···X	H···A (Å)	D···A (Å)	∠(DHA) (°)	Occupancy	Tilt of NH_3 group (°)[a]
$(DA)_2MnCl_4$	270	*Equatorial*	2.4213(34)	3.291(37)	166.12(44)		61.4(16)
		Axial 1	2.4668(14)	3.3310(75)	164.12(42)		
		Axial 2	2.3544(15)	3.226(66)	165.2(26)		
	330	*Equatorial*	2.6051(17)	3.384(20)	146.53(74)		67.66(92)
		Axial 1	2.7753(4)	3.622(29)	158.8(17)		
		Axial 2	2.7215(22)	3.3332(84)	127.02(52)		
$(NA)_2CuBr_4$	270	*Equatorial*	2.7385(5)	3.5262(45)	148.22(31)	Chain A *Part 1* (0.35)	50.682(60)
		Axial 1	3.0803(7)	3.9226(54)	158.73(35)		
		Axial 2	2.7546(6)	3.4350(48)	134.20(31)		
		Equatorial	2.5836(6)	3.431(5)	159.52(31)	Chain A *Part 2* (0.65)	57.37(39)
		Axial 1	2.6022(6)	3.4350(48)	156.18(31)		
		Axial 2	2.9046(7)	3.7516(55)	159.55(35)		
		Equatorial	2.6289(6)	3.4424(55)	152.25(31)	Chain B *Part 1* (0.53)	54.76(41)
		Axial 1	2.5648(6)	3.3696(49)	150.76(31)		
		Axial 2	2.7882(7)	3.6367(55)	159.92(35)		
		Equatorial	2.6929(6)	3.5496(50)	161.86(31)	Chain B *Part 2* (0.47)	81.09(88)
		Axial 1	2.7716(6)	3.3696(49)	125.71(31)		
		Axial 2	3.3243(7)	4.0423(54)	139.38(35)		
	335	*Equatorial*	2.6419(12)	3.506(21)	162.449(3)		70.7(15)
		Axial 1	3.1158(5)	3.9023(25)	147.952(4)		
		Axial 2	3.1905(10)	3.8858(25)	136.106(1)		

[a]The NH_3 tilt angle is defined as the angle between a line connecting the atoms N and C1 and a plane through the metal atoms of the inorganic layers.

Supplementary Table 21. Crystallographic data for $(DA)_2MnCl_4$ collected at 100 K, 270 K, and 330 K.

	**$(DA)_2MnCl_4$**	**$(DA)_2MnCl_4$**	**$(DA)_2MnCl_4$**
Formula	$C_{20}H_{48}Cl_4MnN_2$	$C_{20}H_{48}Cl_4MnN_2$	$C_{20}H_{48}Cl_4MnN_2$
Temperature (K)	100(2)	270(2)	330(2)
Crystal System	Triclinic	Monoclinic	Orthorhombic
Space Group	$P\overline{1}$	$C2/m$	$Cccm$
a (Å)	5.0548 (6)	7.1857 (6)	7.3264 (5)
b (Å)	5.0626 (6)	7.3100 (6)	57.601 (15)
c (Å)	26.419 (3)	26.685 (2)	7.2735 (6)
α (°)	95.516 (4)	90	90
β (°)	92.683 (4)	95.150 (1)	90
γ (°)	90.904 (4)	90	90
V (Å^3)	672.07 (14)	1396.0 (2)	3069.5 (9)
Z	1	2	4
Radiation, λ (Å)	MoKα, 0.71073	MoKα, 0.71073	MoKα, 0.71073
μ (mm^{-1})	0.90	0.86	0.79
Crystal Size (mm)	0.18 × 0.12 × 0.08	0.18 × 0.12 × 0.08	0.18 × 0.12 × 0.08
Max. and min. transmission	0.767 and 0.635	0.767 and 0.492	0.801 and 0.478
Completeness to 2Θ	98.6% (2Θ = 25.123°)	98.5% (2Θ = 25.191°)	97.1% (2Θ = 25.607°)
No. of measured, independent and observed [$I > 2\sigma(I)$] reflections	8898, 2353, 1872	11186, 1345, 1062	18017, 1533, 928
R_{int}	0.055	0.061	0.100
$(\sin\theta/\lambda)_{max}$ (Å^{-1})	0.597	0.599	0.608
Data / Restraints / Parameters	2353 / 134 / 238	1345 / 0 / 124	1533 / 170 / 116
Goodness of Fit on F^2	1.10	1.05	1.08
R_1[a], wR_2[b] [$I > 2\sigma(I)$]	0.066, 0.158	0.061, 0.170	0.097, 0.231
R_1[a], wR_2[b] (all data)	0.086, 0.171	0.074, 0.181	0.143, 0.259
Largest Diff. Peak and Hole (e Å^{-3})	1.32 and –0.65	1.64 and –0.77	0.78 and –0.41

[a] $R_1 = \sum||F_o| - |F_c||/\sum|F_o|$. [b] $wR_2 = \{\sum[w(F_o^2 - F_c^2)^2]/\sum[w(F_o^2)^2]\}^{1/2}$.

Supplementary Table 22. Crystallographic data for $(NA)_2CuBr_4$ collected at 100 K, 270 K, and 335 K.

	**$(NA)_2CuBr_4$**	**$(NA)_2CuBr_4$**	**$(NA)_2CuBr_4$**
Formula	$C_{18}H_{44}Br_4CuN_2$	$C_{18}H_{44}Br_4CuN_2$	$C_{18}H_{44}Br_4CuN_2$
Temperature (K)	100(2)	270(2)	335(2)
Crystal System	Triclinic	Triclinic	Orthorhombic
Space Group	$P\bar{1}$	$P\bar{1}$	*Cmca*
a (Å)	7.4107 (7)	7.6549 (2)	49.155 (3)
b (Å)	7.9092 (8)	7.9674 (2)	7.7844 (5)
c (Å)	23.054 (2)	23.2541 (7)	7.7512 (5)
α (°)	82.835 (2)	80.4332 (9)	90
β (°)	82.901 (3)	81.4664 (9)	90
γ (°)	89.808 (2)	89.8532 (8)	90
V (Å^3)	1330.3 (2)	1382.70 (7)	2966.0 (3)
Z	2	2	4
Radiation, λ (Å)	MoKα, 0.71073	MoKα, 0.71073	MoKα, 0.71073
μ (mm^{-1})	6.83	6.58	6.13
Crystal Size (mm)	0.24 × 0.12 × 0.06	0.24 × 0.12 × 0.06	0.24 × 0.12 × 0.06
Max. and min. transmission	0.646 and 0.428	0.801 and 0.658	0.694 and 0.290
Completeness to 2Θ	99.3% (2Θ = 25.092°)	99.4% (2Θ = 25.026°)	96.8% (2Θ = 25.004°)
No. of measured, independent and observed [$I > 2\sigma(I)$] reflections	20830, 4693, 3569	15136, 4857, 3313	17040, 1291, 790
R_{int}	0.065	0.042	0.112
$(\sin\theta/\lambda)_{max}$ (Å^{-1})	0.597	0.595	0.595
Data / Restraints / Parameters	4693 / 0 /233	4857 / 988 / 399	1291 / 151 / 103
Goodness of Fit on F^2	1.05	1.03	1.09
R_1^{a}, wR_2^{b} [$I > 2\sigma(I)$]	0.040, 0.094	0.042, 0.085	0.104, 0.234
R_1^{a}, wR_2^{b} (all data)	0.063, 0.104	0.079, 0.099	0.148, 0.261
Largest Diff. Peak and Hole (e Å^{-3})	1.21 and –0.78	0.58 and –0.61	0.46 and –0.47

$^aR_1 = \sum||F_o| - |F_c||/\sum|F_o|$. $^bwR_2 = \{\sum[w(F_o^2 - F_c^2)^2]/\sum[w(F_o^2)^2]\}^{1/2}$.

Supplementary References

1. Chan, L., Morris, G. M. & Hutchison, G. R. Understanding conformational entropy in small molecules. *J. Chem. Theory Comput.* **17**, 2099–2106 (2021).
2. Dannenfelser, R.-M. & Yalkowsky, S. H. Estimation of entropy of melting from molecular structure: A non-group contribution method. *Ind. Eng. Chem. Res.* **35**, 1483–1486 (1996).
3. Ricard, L., Rey-Lafon, M. & Biran, C. Vibrational study of the dynamics of *n*-decylammonium chains in the perovskite-type layer compound decylammonium tetrachlorocadmate $(C_{10}H_{21}NH_3)_2CdCl_4$. *J. Phys. Chem.* **88**, 5614–5620 (1984).
4. Guillaume, F. *et al.* Molecular motions of decylammonium chains in the perovskite type layered compound $(C_{10}H_{21}NH_3)_2MnCl_4$. *Mol. Phys.* **67**, 665–679 (1989).
5. Kind, R. *et al.* Dynamics of the *n*-decylammonium chains in the perovskite-type layer structure compound $(C_{10}H_{21}NH_3)_2CdCl_4$. *J. Chem. Phys.* **71**, 2118–2130 (1979).
6. Turturro, A. & Bianchi, U. Conformational contribution to the entropy of melting. I. Linear chain hydrocarbons. *J. Chem. Phys.* **62**, 1668–1673 (1975).
7. Nagle, J. F. & Goldstein, M. Decomposition of entropy and enthalpy for the melting transition of polyethylene. *Macromolecules* **18**, 2643–2652 (1985).
8. Karasz, F. E., Couchman, P. R. & Klempner, D. On the entropy of fusion and its separation into configurational and volume-change contributions. *Macromolecules* **10**, 88–89 (1977).
9. Jenau, M., Reuter, J., Tamarit, J. L. & Würflinger, A. Crystal and *pVT* data and thermodynamics of the phase transitions of 2-methyl-2-nitropropane. *J. Chem. Soc., Faraday Trans.* **92**, 1899–1904 (1996).
10. Aznar, A. *et al.* Reversible and irreversible colossal barocaloric effects in plastic crystals. *J. Mater. Chem. A* **8**, 639–647 (2019).
11. Moya, X. & Mathur, N. D. Caloric materials for cooling and heating. *Science* **370**, 797–803 (2020).
12. Emre, B. *et al.* Large reversible entropy change at the inverse magnetocaloric effect in Ni-Co-Mn-Ga-In magnetic shape memory alloys. *J. Appl. Phys.* **113**, 213905 (2013).
13. Qian, S. *et al.* A review of elastocaloric cooling: Materials, cycles and system integrations. *Int. J. Refrig.* **64**, 1–19 (2016).
14. Gabbott, P. A Practical Introduction to Differential Scanning Calorimetry. in *Principles and Applications of Thermal Analysis* 1–50 (Wiley, 2008).
15. Höhne, G., Hemminger, W. F. & Flammersheim, H.-J. *Differential Scanning Calorimetry*. (Springer, 2003).
16. Gutfleisch, O. *et al.* Mastering hysteresis in magnetocaloric materials. *Philos. Trans. R. Soc. A* **374**, 20150308 (2016).
17. Moore, J. D., Morrison, K., Sandeman, K. G., Katter, M. & Cohen, L. F. Reducing extrinsic hysteresis in first-order $La(Fe,Co,Si)_{13}$ magnetocaloric systems. *Appl. Phys. Lett.* **95**, 252504 (2009).
18. Hou, X. *et al.* Effect of grain size on the electrocaloric properties of polycrystalline ferroelectrics. *Phys. Rev. Applied* **15**, 054019 (2021).
19. Zhang, Y., Billman, J. & Shamberger, P. J. Size effects in the martensitic transformation hysteresis in Ni–Mn–Sn Heusler alloy films. *Acta Mater.* **180**, 116–125 (2019).
20. Casal, H. L., Cameron, D. G. & Mantsch, H. H. A vibrational spectroscopic characterization of the solid-phase behavior of *n*-decylammonium chloride (n-$C_{10}H_{21}NH_3Cl$) and bis(*n*-decylammonium) tetrachlorocadmate [(n-$C_{10}H_{21}NH_3)_2CdCl_4$]. *J. Phys. Chem.* **89**, 5557–5565 (1985).

21. Ning, G. Characterization of the solid-phase behavior of *n*-nonylammonium tetrachlorocuprate by Fourier transform infrared spectroscopy. *J. Solid State Chem.* **117**, 97–102 (1995).

22. Almirante, C., Minoni, G. & Zerbi, G. Mechanism of solid to liquidlike phase transition of alkyl chains in bilayer systems. An infrared spectroscopic study of tetradecylammonium tetrachloromanganate ($[CH_3(CH_2)_{13}NH_3]_2MnCl_4$) and tetradecylammonium tetrachlorozincate $[CH_3(CH_2)_{13}NH_3]_2ZnCl_4$. *J. Phys. Chem.* **90**, 852–859 (1986).

23. Snyder, R. G. Vibrational spectra of crystalline *n*-paraffins: II. Intermolecular effects. *J. Mol. Spectrosc.* **7**, 116–144 (1961).

24. Snyder, R. G. Vibrational spectra of crystalline *n*-paraffins: Part I. Methylene rocking and wagging modes. *J. Mol. Spectrosc.* **4**, 411–434 (1960).

25. Kang, J.-K., Choy, J.-H. & Rey-Lafon, M. Phase transition behavior in the perovskite-type layer compound (n-$C_{12}H_{25}NH_3)_2CuCl_4$. *J. Phys. Chem. Solids* **54**, 1567–1577 (1993).

26. Zerbi, G. *et al.* Molecular mechanics for phase transition and melting of *n*-alkanes: A spectroscopic study of molecular mobility of solid *n*-nonadecane. *J. Chem. Phys.* **75**, 3175–3194 (1981).

27. Li, C. *et al.* Conformational disorder of organic cations tunes the charge carrier mobility in two-dimensional organic-inorganic perovskites. *Nat. Commun.* **11**, 5481 (2020).

28. Wang, Z. *et al.* Revealing molecular conformation–induced stress at embedded interfaces of organic optoelectronic devices by sum frequency generation spectroscopy. *Sci. Adv.* **7**, eabf8555 (2021).

29. Vacatello, M., Girolamo, M. de & Busico, V. Relationships between structure and properties in long-chain bis(*n*-alkylammonium)tetrabromocuprates(II) and bis(*n*-alkylammonium)tetrabromomanganates(II). *J. Chem. Soc., Faraday Trans. 1* **77**, 2367–2375 (1981).

30. Dupont, J. L., Domanski, P., Lebrun, P. & Ziegler, F. *The Role of Refrigeration in the Global Economy*. 38th Note on Refrigeration Technologies (International Institute of Refrigeration). (2019).

31. Coulomb, D., Dupont, J.-L. & Morlet, V. *The Impact of the Refrigeration Sector on Climate Change*. 35th Note on Refrigeration Technologies (International Institute of Refrigeration). (2017).

32. Oort, M. J. M. V. & White, M. A. General trends in phase transitions in a homologous series. Polymorphism in (n-$C_6H_{13}NH_3)_2MnCl_4$ as determined by adiabatic calorimetry from 18 K to 313 K. *J. Chem. Soc., Faraday Trans. 1* **81**, 3059–3065 (1985).

33. White, M. A., Davies, N. J. & Staveley, L. A. K. Polymorphism in (n-$C_7H_{15}NH_3)_2MnCl_4$ and (n-$C_7H_{15}NH_3)_2CdCl_4$ as determined by heat-capacity measurements from 10 to 325 K. *J. Chem. Soc., Faraday Trans. 2* **79**, 1653–1661 (1983).

34. Busico, V., Carfagna, C., Salerno, V., Vacatello, M. & Fittipaldi, F. The layer perovskites as thermal energy storage systems. *Sol. Energy* **24**, 575–579 (1980).

35. Needham, G. F., Willett, R. D. & Franzen, H. F. Phase transitions in crystalline models of bilayers. 1. Differential scanning calorimetric and X-ray studies of $(C_{12}H_{25}NH_3)_2MCl_4$ and $(NH_3C_{14}H_{29}NH_3)_2MCl_4$ salts (M = Mn^{2+}, Cd^{2+}, Cu^{2+}). *J. Phys. Chem.* **88**, 674–680 (1984).

36. Vacatello, M. & Corradini, P. Relationships between structure and properties of compounds of the type $(RNH_3)_2MX_4$. II. Compounds with M = Mn, X = Cl and R = n-C_9H_{19}, n-$C_{11}H_{23}$, n-$C_{13}H_{27}$, n-$C_{15}H_{31}$, and n-$C_{17}H_{35}$. *Gazz. Chim. Ital.* **104**, 773–780 (1974).

37. Chanh, N. B. *et al.* Polymorphism in organo-metallic bidimensional structures: The bis(*n*-octylammonium) tetrachlorocadmate. *J. Phys. Chem. Solids* **44**, 589–594 (1983).

38. Chanh, N. B., Housty, J. R., Meresse, A., Ricard, L. & Rey-Lafon, M. Polymorphism in the bidimensional compound (n-$C_{16}H_{33}NH_3)_2CdCl_4$. *J. Phys. Chem. Solids* **50**, 829–838 (1989).

39. Lemmerer, A. & Billing, D. G. Synthesis, characterization and phase transitions of the inorganic–organic layered perovskite-type hybrids $[(C_nH_{2n+1}NH_3)_2PbI_4]$, n = 7, 8, 9 and 10. *Dalton Trans.* **41**, 1146–1157 (2012).

40. Billing, D. G. & Lemmerer, A. Synthesis, characterization and phase transitions of the inorganic–organic layered perovskite-type hybrids $[(C_nH_{2n+1}NH_3)_2PbI_4]$ (n = 12, 14, 16 and 18). *New J. Chem.* **32**, 1736–1746 (2008).

41. Chanh, N. B. *et al.* Structural and Vibrational Study of the Phase Transitions in Crystalline bis(n-decylammonium)tetrachlorocuprate. *Mol. Cryst. Liq. Cryst. Sci. Technol., Sect. A* **238**, 93–108 (1994).

42. Salgado-Beceiro, J. *et al.* Near-room-temperature reversible giant barocaloric effects in $[(CH_3)_4N]Mn[N_3]_3$ hybrid perovskite. *Mater. Adv.* **1**, 3167–3170 (2020).

43. Bermúdez-García, J. M. *et al.* Giant barocaloric effect in the ferroic organic-inorganic hybrid [TPrA][Mn(dca)$_3$] perovskite under easily accessible pressures. *Nat. Commun.* **8**, 15715 (2017).

44. Bermúdez-García, J. M. *et al.* Giant barocaloric tunability in $[(CH_3CH_2CH_2)_4N]Cd[N(CN)_2]_3$ hybrid perovskite. *J. Mater. Chem. C* **6**, 9867–9874 (2018).

45. Li, B. *et al.* Colossal barocaloric effects in plastic crystals. *Nature* **567**, 506–510 (2019).

46. Lloveras, P. *et al.* Colossal barocaloric effects near room temperature in plastic crystals of neopentylglycol. *Nat. Commun.* **10**, 1803 (2019).

47. Li, J. *et al.* Reversible barocaloric effects over a large temperature span in fullerite C_{60}. *J. Mater. Chem. A* **8**, 20354–20362 (2020).

48. Lloveras, P. *et al.* Giant barocaloric effects at low pressure in ferrielectric ammonium sulphate. *Nat. Commun.* **6**, 1–6 (2015).

49. Aznar, A. *et al.* Giant barocaloric effects over a wide temperature range in superionic conductor AgI. *Nat. Commun.* **8**, 1–6 (2017).

50. Lin, J. *et al.* Giant room-temperature barocaloric effect at the electronic phase transition in $Ni_{1-x}Fe_xS$. *Mater. Horiz.* **7**, 2690–2695 (2020).

51. Stern-Taulats, E. *et al.* Barocaloric and magnetocaloric effects in $Fe_{49}Rh_{51}$. *Phys. Rev. B* **89**, 214105 (2014).

52. Aznar, A. *et al.* Giant barocaloric effect in all-d-metal Heusler shape memory alloys. *Phys. Rev. Mater.* **3**, 044406 (2019).

53. Romanini, M. *et al.* Giant and reversible barocaloric effect in trinuclear spin-crossover complex Fe_3(bntrz)$_6$(tcnset)$_6$. *Adv. Mat.* **33**, 2008076 (2021).

54. Vallone, S. P. *et al.* Giant barocaloric effect at the spin crossover transition of a molecular crystal. *Adv. Mater.* **31**, 1807334 (2019).

55. Moya, X., Kar-Narayan, S. & Mathur, N. D. Caloric materials near ferroic phase transitions. *Nat. Mater.* **13**, 439–450 (2014).

56. Hess, T. *et al.* Thermal hysteresis and its impact on the efficiency of first-order caloric materials. *J. Appl. Phys.* **127**, 075103 (2020).
